# Cellular processes involved in lung cancer cells exposed to direct current electric field

**DOI:** 10.1038/s41598-020-62332-0

**Published:** 2020-03-24

**Authors:** Huijuan Li, Shibin Liu, Xue Yang, Yongqian Du, Jiezhang Luo, Jie Tan, Yulong Sun

**Affiliations:** 1 School of Electronics and Information, Northwestern Polytechnical University, Xi’an, 710072 China; 2 Key Laboratory for Space Biosciences & Biotechnology, School of Life Sciences, Northwestern Polytechnical University, Xi’an, 710072 China

**Keywords:** Gene ontology, Non-small-cell lung cancer

## Abstract

With the rapid breakthrough of electrochemical treatment of tumors, electric field (EF)-sensitive genes, previously rarely exploited, have become an emerging field recently. Here, we reported our work for the identification of EF-sensitive genes in lung cancer cells. The gene expression profile (GSE33845), in which the human lung cancer CL1-0 cells were treated with a direct current electric field (dcEF) (300 mV/mm) for 2 h, was retrieved from GEO database. Differentially expressed genes (DEGs) were acquired, followed by Gene Ontology (GO), Kyoto Encyclopedia of Genes and Genomes pathway (KEGG) and protein-protein interaction (PPI) analysis. Hub genes were acquired and analyzed by various tools including the Human Protein Atlas, Kaplan-Meier analysis, Cytoscape, FunRich, Oncomine and cBioPortal. Subsequently, three-dimensional protein models of hub genes were modeled by Modeller 9.20 and Rosetta 3.9. Finally, a 100 ns molecular dynamics simulation for each hub protein was performed with GROMACS 2018.2. A total of 257 DEGs were acquired and analyzed by GO, KEGG and PPI. Then, 10 hub genes were obtained, and the signal pathway analysis showed that two inflammatory pathways were activated: the FoxO signaling pathway and the AGE-RAGE signaling pathway. The molecular dynamic analysis including RMSD and the radius of gyration hinted that the 3D structures of hub proteins were built. Overall, our work identified EF-sensitive genes in lung cancer cells and identified that the inflammatory state of tumor cells may be involved in the feedback mechanism of lung cancer cells in response to electric field stimulation. In addition, qualified three-dimensional protein models of hub genes were also constructed, which will be helpful in understanding the complex effects of dcEF on human lung cancer CL1-0 cells.

## Introduction

Lung cancer is a malignant tumor of the lung characterized by the growth of cells out of control in the lung tissue. Among all tumor types, lung cancer is extremely harmful. In the United States, lung cancer is the most common cause of death among men (76,650, 24%) and women (66,020, 23%), and 25% of all cancer-caused deaths are due to lung cancer^1^. An important factor in the great harm caused by lung cancer is that lung cancer is prone to metastasis^2^. Because of the anatomical features of the lungs, namely the rich blood supply and lymphatic drainage system, lung cancer can be transferred through a variety of ways including blood transfer, lymphatic metastasis, direct spread and airway transfer^2^. Therefore, revealing the molecular mechanism of lung cancer metastasis has become one of the major challenges faced by researchers.

In recent years, research on the effects of electric fields on tumor cells has made a series of breakthroughs^3,4^. Research in this field can lay the foundation for the electrochemical treatment of tumors, and thus provide the experimental basis for the development of more efficient electrochemical therapy^5^. Therefore, it has become one of the most concerned focuses of researchers in the field of cancer research^6^.

Electric fields generally fall into two categories: direct current electric field (dcEF) and alternating current electric field (acEF). Up to now, there are many studies on the effects of acEF on tumor cells, and interesting research results have been obtained^7–9^. However, research on the biological effects of dcEF is still relatively rare.

Regarding the influence of dcEF on lung cancer cells, the predecessors have carried out interesting work. Ji-Yen Cheng *et al*. treat the human lung cancer cell line CL1–5 with dcEF (300 mV/mm) for 2 h and show that some genes can be regulated by dcEF, which may be involved in the electrotactic response of lung cancer cells to dcEF^10^. Subsequently, Mengsu Yang group stimulate the non-small cell lung cancer cell lines (H1299, H1975, H460 and HCC827) with dcEF (200–600 mV/mm) for 3 h, and find that these lung cancer cell lines exhibit cell-type dependent different response to applied dcEFs^11^. Very recently, Ji-Yen Cheng *et al*. investigate the electrotaxis of several non-small cell lung cancers (NSCLC) strains, including human lung squamous cell carcinoma NCI-H520 cells and human large cell lung carcinoma NCI-H460. After dcEF stimulation (300–375 mV/mm, 2–6 h), lung cancer cells (NCI-H520 and NCI-H460) move towards the cathode, while non-cancerous MRC-5 lung fibroblasts migrate towards the anode^6^. Also, HE Yong *et al*. evaluate the effects of dcEF on the migration and proliferation of lung cancer H1975 cells, and they show that the exposure of dcEF (100 mV/mm) for 1 h induces proliferation and migration of H1975 cells^12^.

However, most studies mainly focus on exploring changes in cell behavior such as migration, polarization and electrotaxis, whereas there are few studies on signaling pathways for lung cancer cell migration under dcEF^4,13^. In addition, according to our latest investigation, no research has yet been found focusing on the construction of qualified protein models of electric field-sensitive genes, which hinders the development of pharmacological antagonists or agonists against these sensitive proteins.

In the present study, by using multiple in silico methods, three levels of research have been integrated to investigate the molecular mechanism by which electric fields affect human lung cancer CL1–0 cells.

First of all, at the genetic level. After DEGs were obtained, GO (gene ontology) enrichment analysis was performed to classify differentially expressed genes according to their functions, and to annotate and classify these genes^14^. Next, considering that there were a large number of DEGs currently available and genes often played various physiological roles together in the form of a network^15^, the interaction between networks composed of DGEs was examined by KEGG analysis.

Secondly, at the protein level. As the main performer of various physiological functions in cells, the proteins corresponding to DEGs (referred to as “DEGs proteins”) were studied intensively. Due to a large number of DEGs proteins currently available and protein-protein interaction (PPI) is one of the effective methods to find the way of interaction between multiple proteins^16^, the STRING database and its plug-ins (clueGO + Cluepedia plug-in) were used to screen PPI networks formed by DEGs proteins, and the top 10 hub nodes with higher degrees were eventually acquired. Subsequently, with the help of 3D modeling and molecular dynamics simulation methods, the most stable conformations of the proteins of the 10 hub genes were obtained.

Finally, at the clinical significance level. In order to explore the significance of these DEGs, the expression of hub genes in human tumor tissues and their predictive significance in patient prognosis were investigated using a variety of online tumor databases (including cBioportal, Oncomine and KMplot).

Taken together, by using integrated bioinformatics and molecular dynamics methods, we identified EF-sensitive genes in lung cancer cells and constructed qualified three-dimensional protein models of these hub genes. Our work would not only help to understand the mechanism of dcEF-induced changes in tumor cells, but also be useful in pointing out that the inflammatory state of tumor cells may be involved in the feedback of the cells to the dcEF stimulation.

## Materials and Methods

### Research process

In this study, the electric field-sensitive genes of lung cancer cells were obtained by integrated bioinformatic analysis and were subsequently subjected to molecular dynamics simulation to acquire more structural information characteristics and to construct 3D protein models. This study consists of two main steps:

In the first step of the study, the electric fields-sensitive genes were acquired by using bioinformatics, followed by comprehensive bioinformatics analysis to exploit these hub gene’s characteristics and signaling mechanisms. Briefly, the series matrix file (s) and the platforms of GPL570 files (GSE33845) were originally retrieved from the Gene Expression Omnibus (GEO, http //:www.ncbi.nlm.nih.gov/geo/), and the samples were divided into control samples and experimental samples for further analysis. Then, the differentially expressed genes (DEGs) were screened using GEO2R tool, followed by analyzing with gene ontology (GO) and pathway enrichment study. Next, PPI networks were drawn by String (http://string-db.org/), Cytoscape and FunRich software, and 10 hub genes were finally acquired. At last, the cellular process of hub genes were investigated by using various tools, including the Human Protein Atlas, Kaplan Meier analyses, Oncomine and cBioPortal.

In the second step of the study, molecular dynamics simulations were used to study the movements of these electric field-sensitive proteins on a microscopic scale. Firstly, with the help of Modeller 9.20 and Rosetta 3.9, the three-dimensional structural models of these hub proteins were constructed. Subsequently, the 100 ns molecular dynamics simulation for each hub protein was performed by using the GROMACS 2018.2 software package. Finally, detailed structural features of these hub proteins were investigated by analyzing trajectories.

### Microarray data

**Figure 1:**
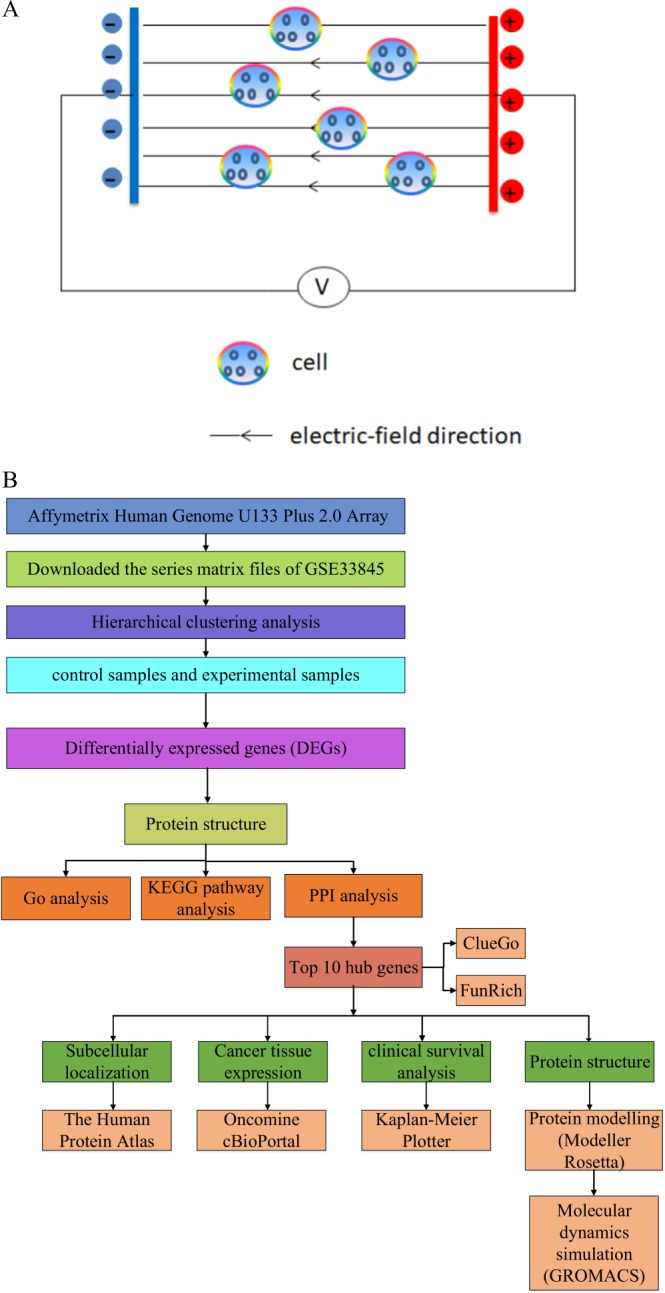
Schematic diagram. (**A**) Schematic diagram of human lung cancer cell CL1-0 in an external physical electric field. Cells were exposed to a dcEF (300 mV/mm) for 2 h, and then cells were subjected to microarray analysis. (**B**) Schematic overview of the work flow.

The gene expression profiles of GSE33845 were downloaded from GEO database. GSE33845 was submitted by Huang C *et al*., which was based on GPL570 platform (Affymetrix GeneChip Human Genome U133 Plus 2.0 Array)^10^. The lung cancer CL1-0 cells were divided into two groups: the experimental group was exposed to dcEF (300 mV/mm) for 2 h, and the other group was the control group (Fig. 1A).

### Identification of DEGs

The analysis was performed using GEO2R tool (https://www.ncbi.nlm.nih.gov/geo/geo2r). Hierarchical clustering analysis was applied to categorize the data into samples from the experimental group and the control group. A classical test to identify DEGs with a change ≥2.0 was chosen, and a *p*-value cutoff of <0.05 was defined to be statistically significant.

### Functional enrichment analysis for DEGs

Metascape is a web-based resource for gene annotation analysis (http://metascape.org/), which queries abundant databases including KEGG pathways, GO functional and Hallmark Gene Sets^17–19^. In order to identify the potential mechanism and predominant biological processes associated with DEGs, pathway enrichment analysis of genes was performed with Metascape online tool. Moreover, the main GO categories (molecular function (MF), cellular component (CC) and biological process (BP)) were analyzed by the PANTHER classification tool^20^.

KEGG is a collection of manually generated pathway database for investigating molecular interaction networks. In this study, we used the KEGG to analyze the functional enrichment of DEGs. In addition, a Cytoscape plug-in called KEGGParser was also employed to study the biological networks.

### Integration of Protein-Protein Interaction (PPI) network and module analysis

The STRING database (http://string-db.org) aims to provide a critical assessment and integration of protein-protein interactions (PPI), including direct (physical) as well as indirect (functional) associations^21^. STRING (version 10.0) covers 9,643,763 proteins from 2031 organisms (1,678 bacteria, 238 eukaryotes and 115 archaea) and 932,553,897 total interactions. In order to assess the interactive relationship among DEGs, DEGs were mapped to STRING. Only experimentally validated interactions with a combined score >0.4 were chosen as significant. Subsequently, the PPI network was constructed using the Cytoscape software. The plug-in Molecular Complex Detection (MCODE) should be opened, which was used to screen the modules of PPI network in Cytoscape. The criteria were set as follows: the number of nodes >4 and MCODE scores >3. Furthermore, enrichment analyses were performed for DEGs in the modules. Only *P* < 0.05 was considered to have a striking difference.

To further reveal the pathways that may be associated with the identified DEGs, pathway enrichment analyses were analyzed by Cytoscape software with the clueGO+Cluepedia plug-in. ClueGO plug-in (http://apps.cytoscape.org/apps/cluego) can explore the molecular mechanisms of large gene lists by identifying significant KEGG and gene ontology (GO) terms pathways^22^. The Cluepedia plug-in (http://apps.cytoscape.org/apps/cluepedia) has an expressive and intuitive visualization, which is used to search for pathway-associated markers^23^. In this study, KEGG pathway enrichment analysis was performed using CluePedia and ClueGO tool kits, and a *p-*value of <0.05 and a kappa coefficient of 0.4 were considered as threshold values.

### Exploring lung cancer genomics data by cBio Cancer Genomics Portal

The cBio Cancer Genomics Portal (http://cbioportal.org) is a user-friendly, open-access platform that provides a web resource for analyzing, visualizing and exploring multidimensional cancer genomics data^24^. Researchers can acquire the complex cancer genomics and clinical profiles through the query interface of the portal. In our study, the candidate genes were analyzed by cBio Cancer Genomics Portal to investigate their clinical information.

### The hub genes analysis by using the Oncomine database

In this study, the hub gene was analyzed using the Oncomine database for the following reason: using the Oncomine database for analysis could help understand the oncological genetic characteristics of the hub gene in lung cancer. The hub genes were considered to be key genes in many DEGs of human lung cancer tumor CL1-0 cells affected by the electric field. Therefore, various methods were used to comprehensively examine the characteristics of these hub genes. Considering the well-known characteristics of tumor cells-strong variability^25^, a database providing comprehensive gene expression characteristics for multiple tumor types was needed. The Oncomine (www.oncomine.org) database is an online collection of microarrays from many “multi-arrays” cancer-related and various sources, which enables multiple comparisons of gene expression (RNA or DNA) between various studies^26^. Hence, the Oncomine database was used to analyze the mRNA expression profiles of hub genes in lung cancer. The genes were queried in the database and the results were filtered by selecting lung cancer *vs*. normal analysis.

### Hub protein modeling

Hub protein sequences were obtained from NCBI, and the Blast module was used to align the hub protein sequences with PDB database^27^. For each hub protein, 3 templates (query cover >30%) were selected as templates of next homology modeling (Table S1). Three-dimensional homology models of hub proteins were built using Modeller (9v20)^28^. Multiple-templates modeling methods, including Salign, Align2d and Model modules were employed for the modeling process. For each hub protein, 100 candidate models were constructed and the best 1 model was selected based on scores calculated from discrete optimized protein energy (DOPE).

### De novo modeling of JUN

Considering that the blast result of JUN from NCBI (query cover <30%) is too poor to start multiple-template modeling using Modeller (9v20), we used the ROSETTA3.9 (*de novo modeling* script)^29^ to generate 3D protein models for JUN. A total of 850 candidate models were generated and the one with the lowest score was chosen as the best theoretical model for JUN.

### Molecular dynamics simulations: protein in water

MD simulation of the hub proteins was performed by using GROMACS2018.2 package^30^ in Linux environment. Different hub proteins were performed at the similar condition with various minor modifications. The protein was fully solvated in the system of an octahedron box with a size of 1.0 nm by SPC simple point charge water molecules to provide an aqueous environment. The system was neutralized by adding Cl^−^ or Na^+^ ions and periodic boundary conditions were employed in all directions. Energy minimization of the protein was conducted with the steepest descent for 50000 steps with the max force less than 100 KJ/mol. The system was set to the equilibration phases using NVT (50 ps, 300 K) and NPT (100 ps, 300 K, 1.0 bar) respectively. Molecular dynamics and simulation run was conducted for 100 ns to study the structural and energy situation.

The potential of trajectory acquired after MD simulation was investigated using GROMACS utilities to produce the RMSD and radius of gyration. Xmgrace tool was used to obtain various plots. Ramachandran plot analysis was performed with PROCHECK Ramachandran plots^31^ (http://www.ebi.ac.uk/thornton-srv/databases/pdbsum/Generate.html). The three-dimensional protein structures were produced by Pymol (www.pymol.org).

## Results

### Identification of DEGs

**Figure 2:**
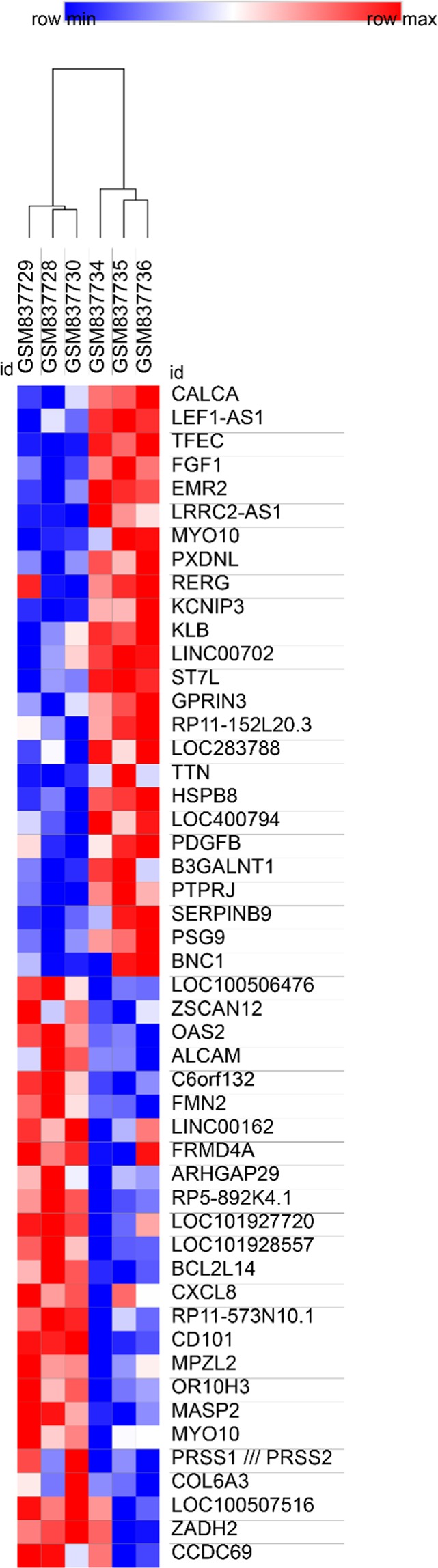
Heatmap of the top 50 differentially expressed genes (25 down-regulated genes and 25 up-regulated genes). Blue: down-regulation; red: up-regulation.

Our study workflow was shown in Fig. 1B. The differentially expressed genes (DEGs) were acquired by using GEO2R, where the criteria were set as follows: *P* < 0.05, fold control (FC) ≥ 2.0. A total of 257 genes were identified, which included 161 up-regulated genes and 96 down-regulated genes. In the analysis of different genes expressed by different experimental treatments, the heat map was used to show the changes in expression levels of multiple genes caused by dcEF treatment (300 mV/mm, 2 h) and untreated groups. As shown in Fig. 2, the heatmap of DEGs (top 25 down-regulated genes and 25 up-regulated genes) were obtained with the online software Morpheus (https://software.broadinstitute.org/morpheus/) (due to space limitations, not all genes were shown).

**Figure 3:**
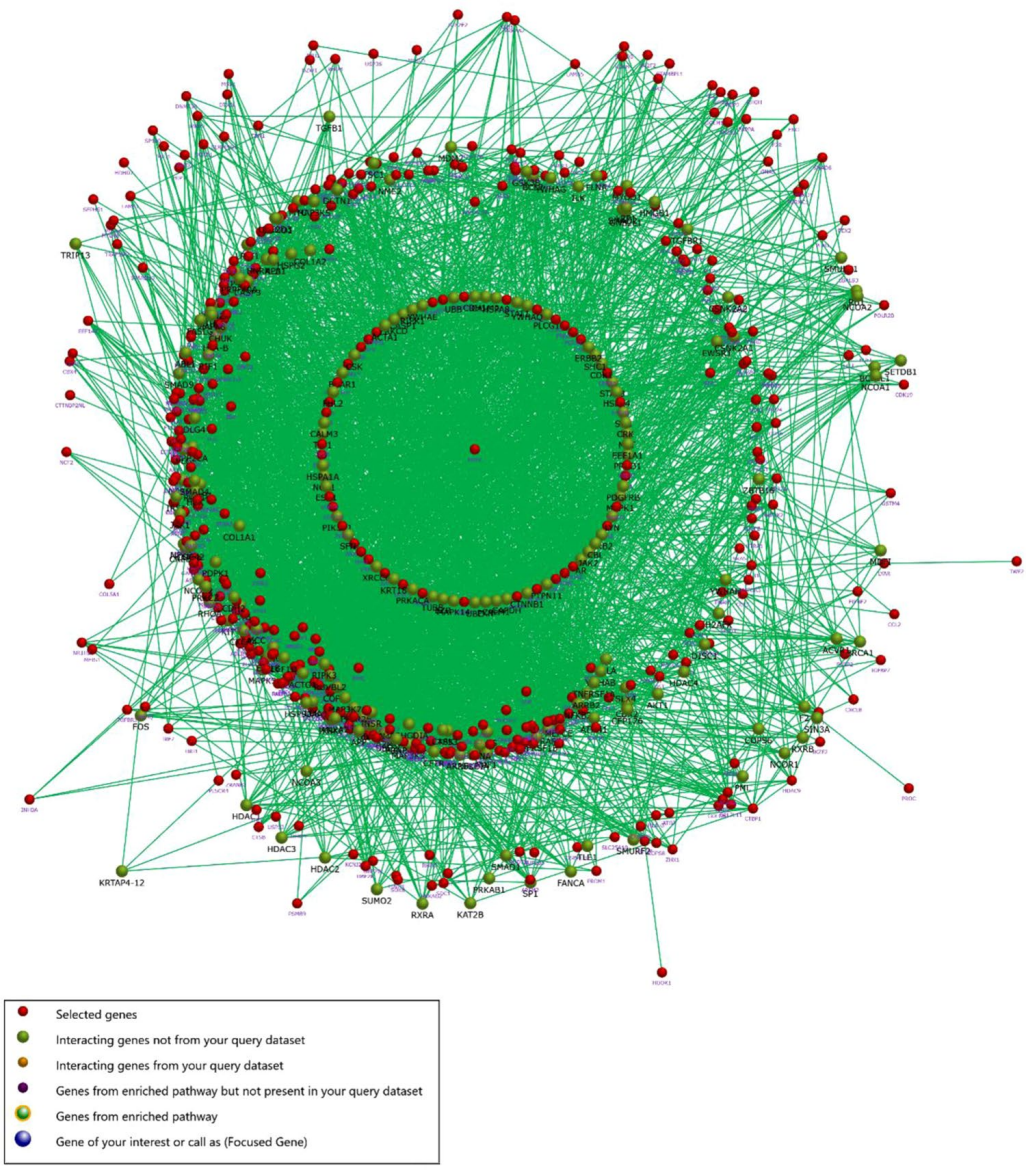
The interaction network analysis of differentially expressed genes.

Subsequently, the file of DEGs was imported directly into FunRich (http://www.funrich.org) tool for functional enrichment and interaction network analysis^32–34^, and those nodes less than 10 interactions were presented in Fig. 3.

### Functional and pathway enrichment analysis of identified modules associated with DEGs

**Figure 4:**
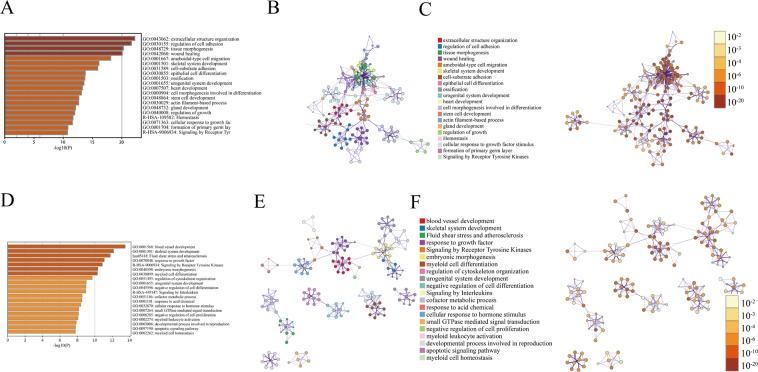
The enrichment analysis was performed by Metascape. (**A**) Bar graph of enriched terms of the up-regulated genes (colored by *p*-values). (**B**) The network of enriched terms (colored by cluster ID) of the up-regulated genes. (**C**) The network of enriched terms (colored by *p*-value) of the up-regulated genes. (**D**) Bar graph of enriched terms of the down-regulated genes (colored by *p*-values). (**E**) The network of enriched terms (colored by cluster ID) of the down-regulated genes. (**F**) The network of enriched terms (colored by *p*-value) of the down-regulated genes.

Functional enrichment analysis of the DEGs was performed using the Metascape^17–19^. As shown in Fig. 4, the significantly enriched pathways of DEGs contained extracellular structure organization, regulation of cell adhesion and tissue morphogenesis (Fig. 4A). In addition, the network was visualized by Cytoscape, where each node represented an enriched term and the node’s color indicated its *p-*values and cluster ID (Fig. 4B,C). For the down-regulate DEGs, the significantly enriched signaling pathways consisted of blood vessel development, skeletal system development, fluid shear stress and atherosclerosis (Fig. 4D). As demonstrated in Fig. 4E,F, each node in the network was highly clustered together.

**Figure 5:**
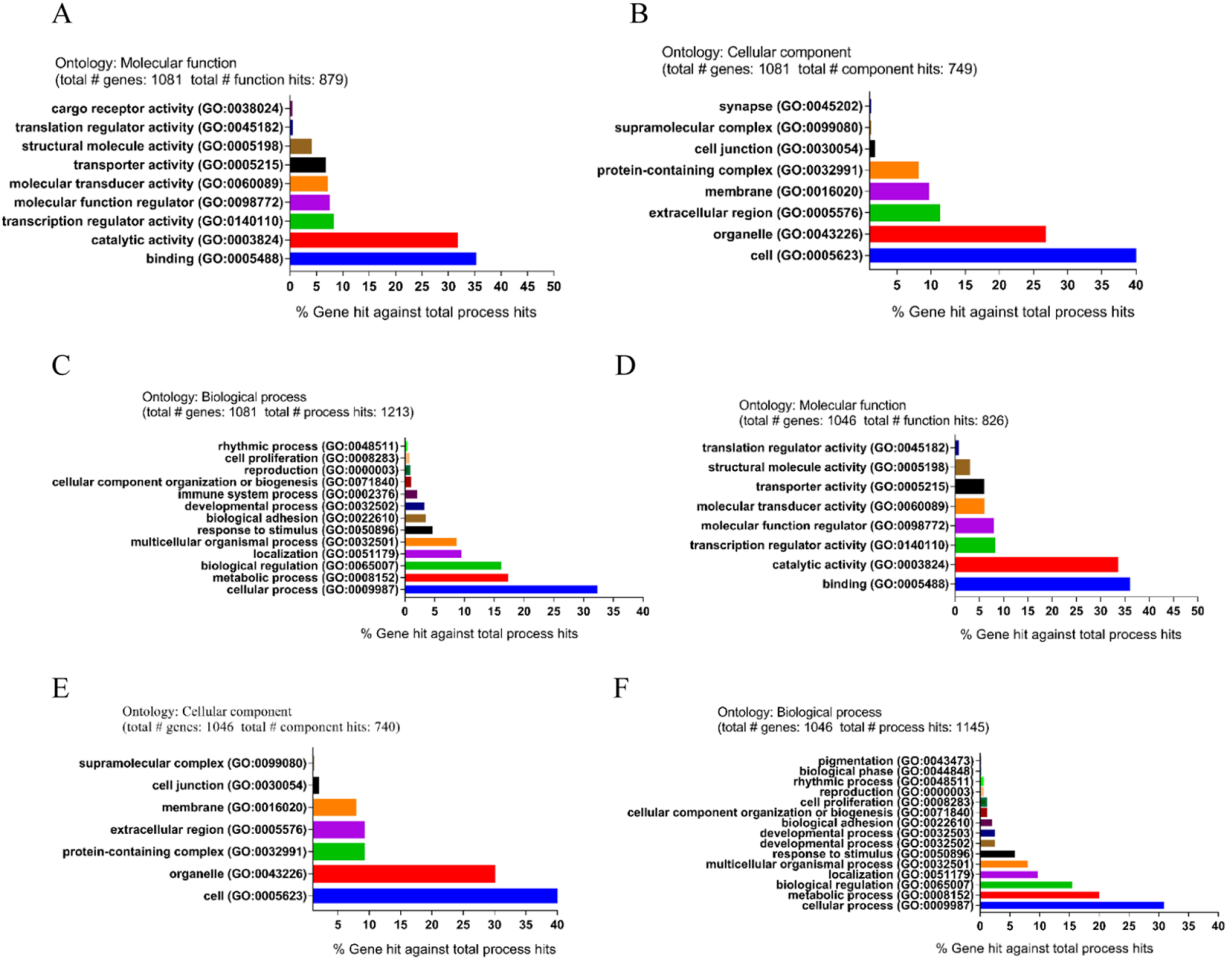
Functional and pathway enrichment analysis of identified modules associated with DEGs. The DEGs were subjected to gene ontology (GO) classification using the PANTHER GO classification system. Up-regulated: (**A**) Molecular function (MF). (**B**) Cellular component (CC). (**C**) Biological process (BP). Down-regulated: (**D**) Molecular function (MF). (**E**) Cellular component (CC). (**F**) Biological process (BP).

According to the PANTHER Go classification system^20,35,36^, DEGs were closely related to various biological processes and molecular functions. The up-regulated genes indicate that the proteins of MF were associated with binding (35.3%), catalytic activity (31.5%) and transcription regulator activity (8.1%). With regard to CC, the majority of proteins were involved in the processes such as cell (42.2%), organelle (26.6%) and extracellular region (11.1%). Regarding of BP, proteins consisted of cellular process (32.4%), metabolic process (17.3%) and biological regulation (16.2%). The down-regulated genes revealed that the proteins of MF were closely linked to binding (36.1%), catalytic activity (33.4%) and transcription regulator activity (8%). For the CC, the majority proteins anticipated in cell (42%), organelle (29.9%) and protein-containing complex (9.1%). With regard to BP, the majority of proteins contained cellular process (30.9%), metabolic process (20%) and biological regulation (15.5%) (Fig. 5).

**Figure 6:**
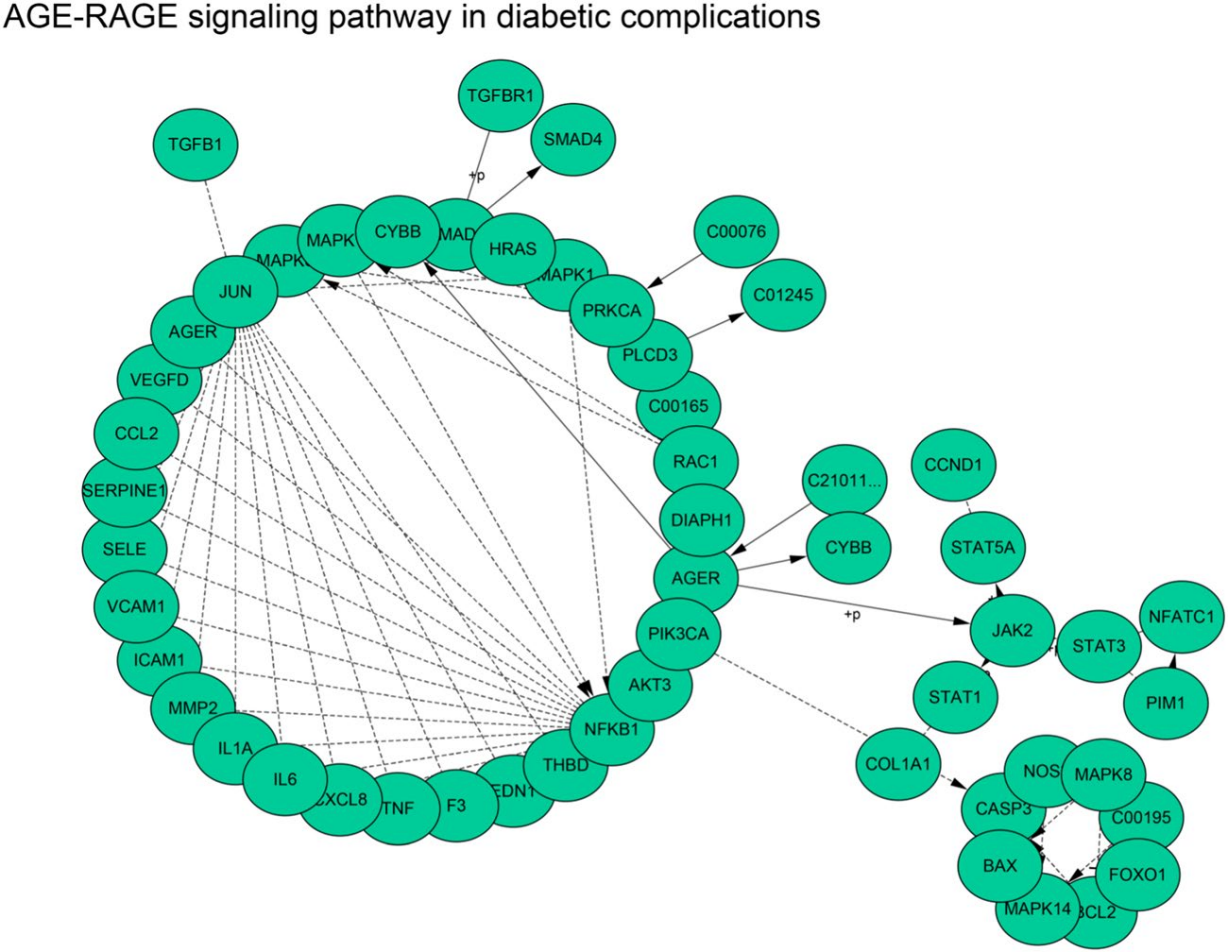
Detailed information of sub-pathway (AGE-RAGE signaling pathway in diabetic complications) in the KEGG.

Table S2 showed the 5 most-significantly enriched pathways of DEGs. The AGE-RAGE signaling pathway in diabetic complications was the most significant pathway from the ClueGO enrichment analysis, and hence were selected for subsequent Cytoscape analysis (Fig. 6).

### Module screening from the PPI network

**Figure 7:**
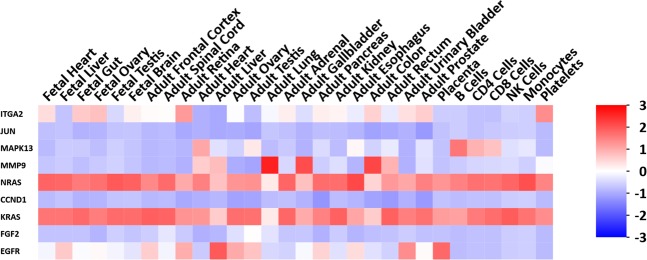
Heat map of the 10 hub genes.

To investigate the protein-protein interactions of DEGs, the STRING database was employed for analysis, followed by the module study^21,37–39^. As shown in Figure S1, the top 10 hub nodes with higher degrees were acquired, including epidermal growth factor receptor (*EGFR*), AP-1 transcription factor subunit (*JUN*), integrin subunit alpha 2 (*ITGA2*), fibroblast growth factor2 (*FGF2*), cyclin D1 (*CCND1*), interleukin6 (*IL6*), KRAS proto-oncogene (*KRAS*), matrix metallopeptidase 9 (*MMP9*), neuroblastoma RAS viral oncogene homolog (*NRAS*) and mitogen-activated protein kinase 13 (*MAPK13*). Among these genes, EGFR showed the highest node degree (score: 189). As shown in Fig. 7, the heatmap was drawn by Funrich to illustrate the expression of the hub genes.

Subsequently, the plug-ins MCODE was used to analyze the various sub-networks of the PPI, which included 1603 nodes and 10506 edges. As demonstrated in Figure S1, the functions of module 1–15 were associated with the following cellular processes: extracellular region part, positive regulation of cell development, positive regulation of cell communication, heparan sulfate proteoglycan biosynthetic process, glutathione transferase activity, GIPase regulator activity, steroid hormone receptor activity, signaling, protein amino acid glycosylation, proteinaceous extracellular matrix, protein kinase activity, proteinaceous extracellular matrix, protein kinase activity, clathrin adaptor complex, positive regulation of fatty acid beta-oxidation, calcium channel activity and nuclesome.

**Figure 8:**
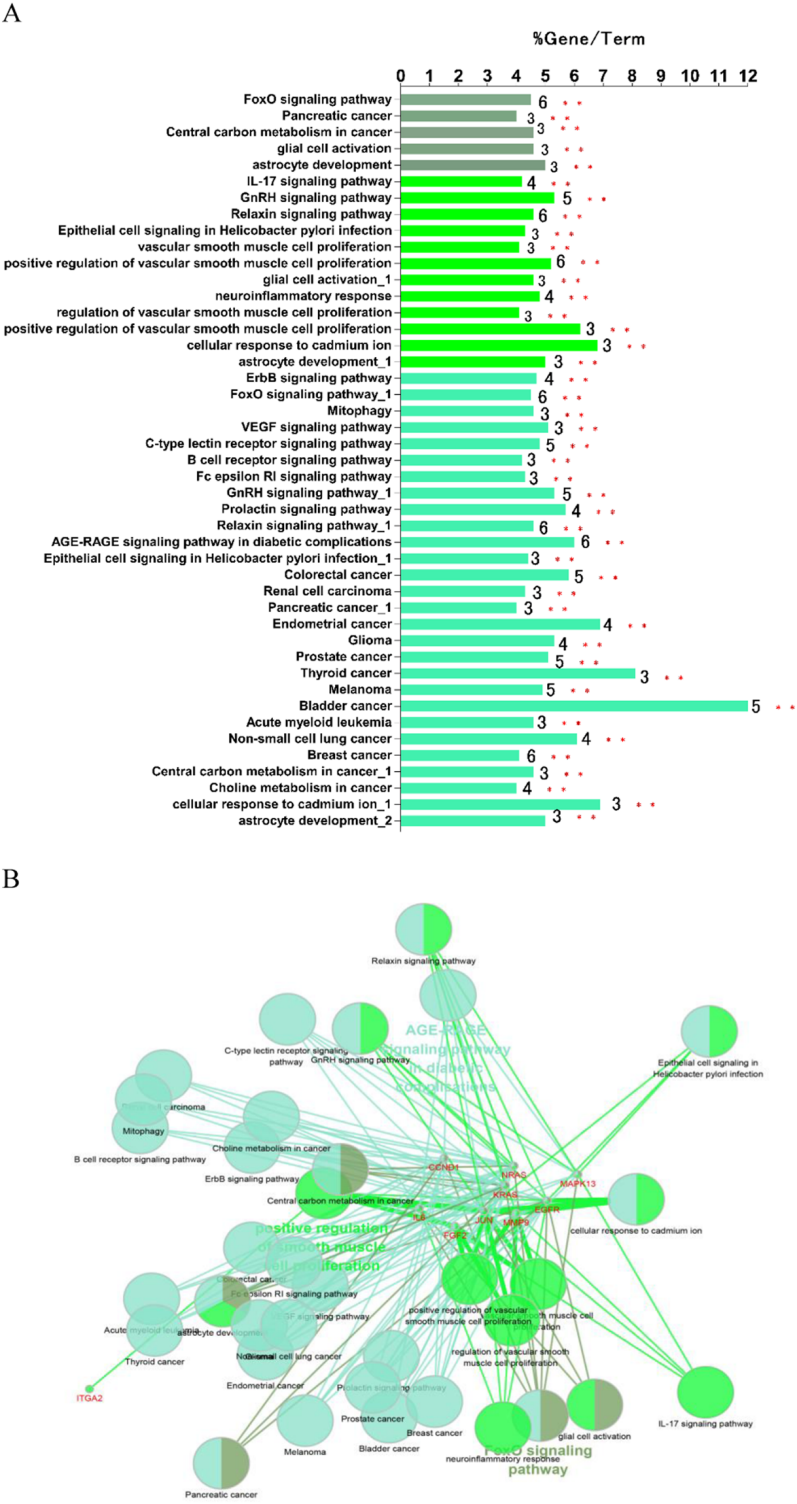
The 10 hub genes were mapped for functional enrichment analysis using ClueGo tool. (**A**) Enriched pathways of 10 genes (different pathways were represented by different colors). (**B**) Pathway enrichment analysis with ClueGO for the 10 hub genes.

In addition, Cluego was also utilized to analyze the functional enrichment of these 10 hub genes. A total of 45 pathways were obtained, which could be grouped into 2 categories: 1. FoxO (forkhead box O, FOXO) signaling pathway; 2. AGE-RAGE signaling pathway in diabetic complications (Fig. 8A,B).

### Sub-localization expression analysis of hub genes

With the help of the online database the Human Protein Atlas (http://www.proteinatlas.org)^40^, we acquired the experimental evidence about the sub-localization of *EGFR, JUN, ITGA2, FGF2, CCND1, IL6, MMP9* and *MAPK13* respectively. Meanwhile, the information for *KRAS* and *NRAS* was not obtained.

**Figure 9:**
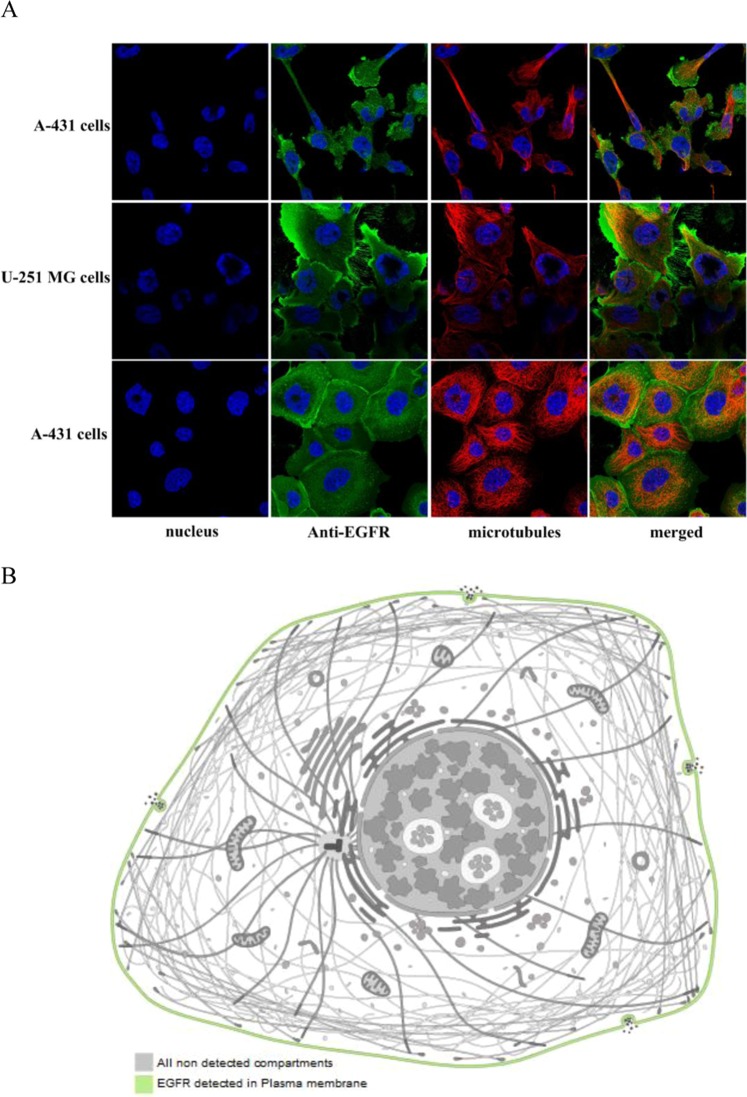
*EGFR* localization in human cells^41^ (https://www.proteinatlas.org/ENSG00000146648-EGFR/cell). (A) The localization of EGFR protein in human cells. Blue: nucleus; Green: *EGFR*; Red: microtubules. (B): Proposed schematic outline of *EGFR* in human cells (The Human Protein Atlas images are licensed under CC BY-SA 3.0 (https://creativecommons.org/licenses/by-sa/3.0/), (https://creativecommons.org/licenses/by-sa/3.0/legalcode)).

The sub-localization of EGFR in human cell line A-431 and U-251 MG, which demonstrated that EGFR protein existed at the plasma membrane of A-431 and U-251 MG cells^41^ (Fig. 9). Figures S2–S8 showed that the sub-localizations of *JUN, ITGA2, FGF2, CCND1, IL6, MMP9* and *MAPK13* in diverse human cell lines^41^, which demonstrated that the hub genes (*JUN, ITGA2, FGF2, CCND1, IL6, MMP9* and *MAPK13*) distributed at different zones of the cells (Table S3).

### Mining genetic alterations connected with lung cancer-associated genes by cBioportal

Although the functional enrichment analysis uncovered the link between dcEFs associated genes and cancer-related pathways, however, detail mechanisms were still needed. To further investigate the validity of this link, cBioportal (an online web-based integrated data mining system) was used to explore the genetic alteration of genes associated with lung cancer^24,42,43^.

**Figure 10:**
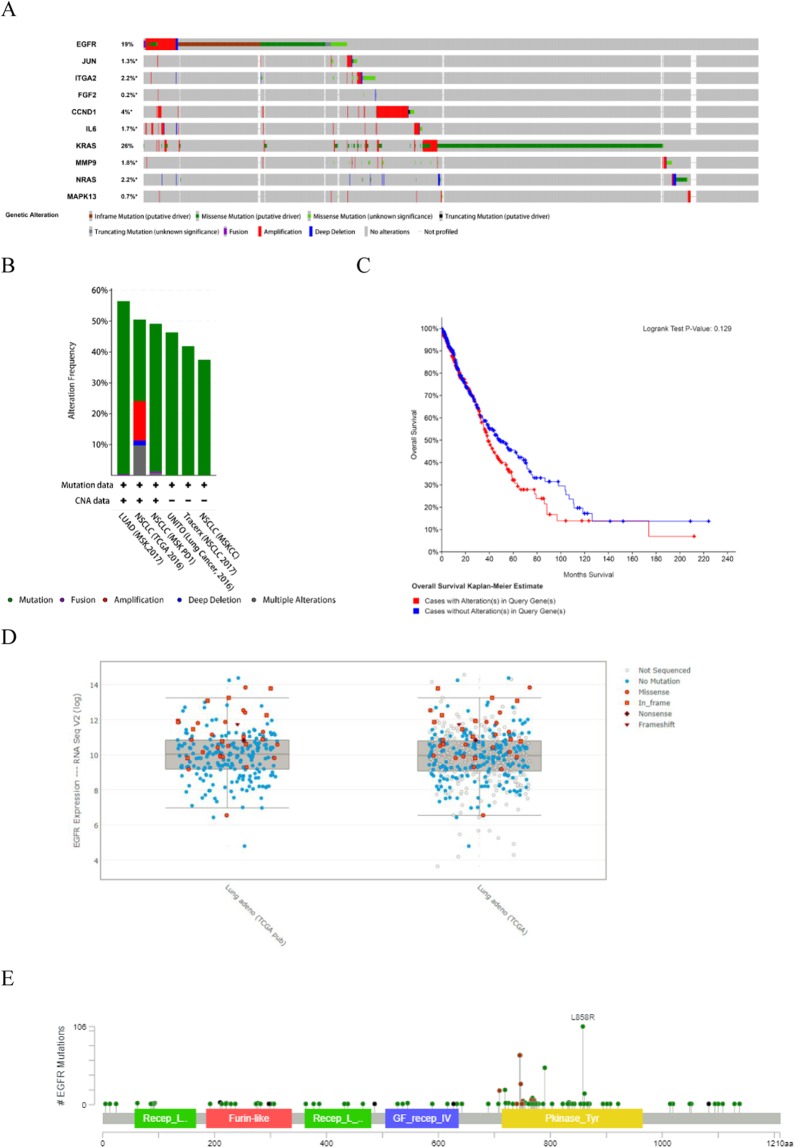
Genetic alterations of the hub genes were analyzed using the cBioPortal. (**A**) Genetic alterations of the hub genes were analyzed using cBioPortal. Grey bars along a vertical line represent the same sample interrogated for amplification (red), deep deletion (blue), missense mutation (green), truncating mutation (black) or Fusion (purple). (**B**) The alteration frequency of a 10-gene signature (*EGFR, JUN, ITGA2, FGF2, CCND1, IL6, MMP9, MAPK13, KRAS and NRAS*) was determined using the cBioPortal (http://www.cbioportal.org). (**C**) Overall Survival Kaplan–Meier estimate. (**D**) Relative expression level as a function of relative copy number of *EGFR* was plotted in different databases. (**E**) The distribution of *EGFR* mutations in non–small-cell lung cancer across protein domains.

Among the six tumor types we used as dataset^44–49^, the expression levels of these hub genes varied from 0.2% to 19% (Fig. 10A), and the mutation frequency of each hub gene was shown in Fig. 10B.

EGFR-related mutations include amplification, deep deletion, inframe mutation and missense mutation. For *JUN* and *ITGA2*, the main changes were amplification, deep deletion and missense mutation. Meanwhile, mutations occurred in *CCND1, IL6* and *KRAS* were amplification and missense mutation. It is worth noting that significant changes in *FGF2, MMP9, MAPK13* and *NRAS* were not found. For details on the specific number of mutations, see Figures S9–S17. In addition, the expression of these hub genes on non-small cell lung cancer cell lines was also demonstrated in Fig. 10C–F.

### Survival analysis of hub genes

**Figure 11:**
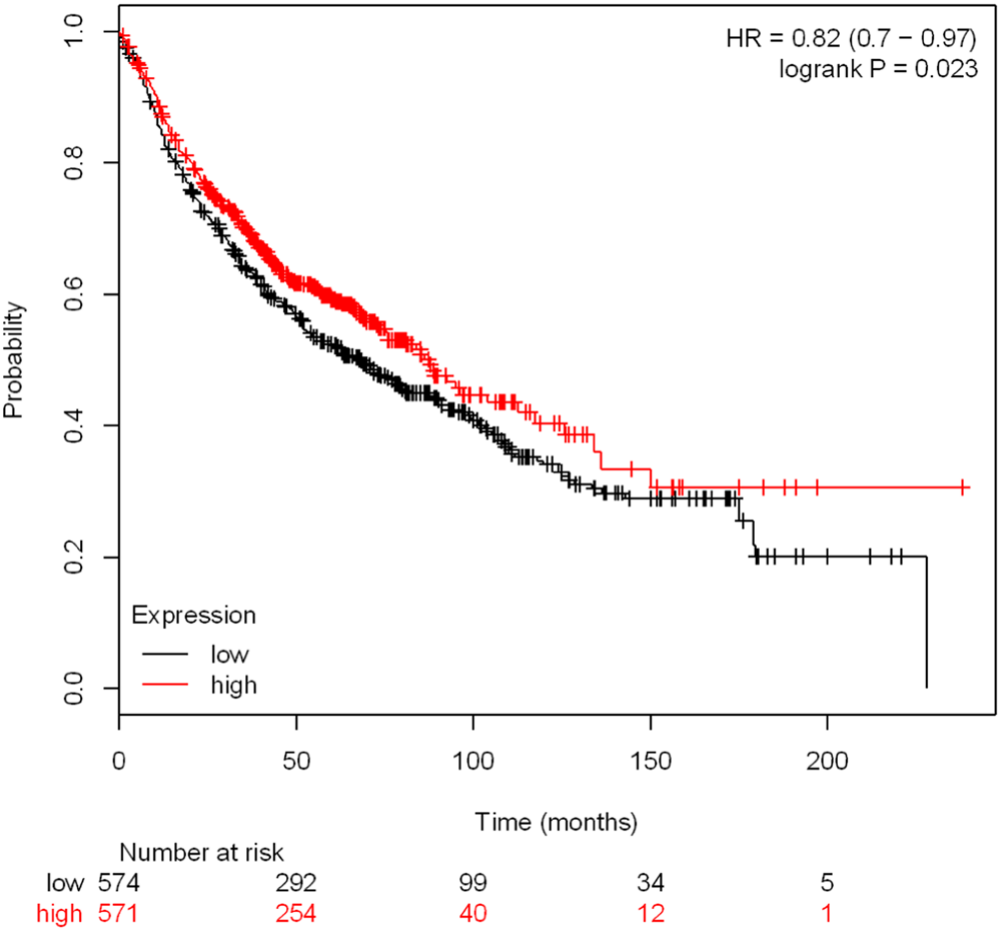
Kaplan–Meier plot for *EGFR* associated with patient survival.

To observe the nature of survival curves and their implications for assessing the value of cancer therapy, the expression survival curves of the tumor suppressor gene were analyzed by using the online tool (Kaplan Meier plotter, http://www.kmplot.com/analysis/). We used this integrative data analysis tool to evaluate the prognostic power of the proliferation-related genes *EGFR* (Fig. 11), *JUN, ITGA2, FGF2, CCND1, IL6, KRAS, MMP9, NRAS* and *MAPK13* (Figures S18–S26). Patients with high levels of several genes (*JUN, FGF2, IL6* and *MAPK13*) have higher survival rates than patients with low expression. Meanwhile, The expression of the other hub genes (*EGFR, CCND1, ITGA2, KRAS, MMP9* and *NRAS*) was not significantly associated with patient survival. These data can provide predictive information for the prognosis of hub gene-associated tumor patients.

### The 10 hub genes expressed in lung cancer analyzed by Oncomine

**Figure 12:**
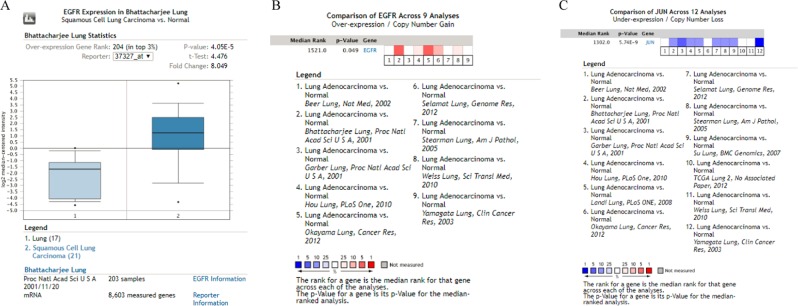
The cDNA microarray analysis was conducted using Oncomine database. (**A**) Comparison of the expression of *EGFR* between lung and squamous cell lung carcinoma samples in the Bhattacharjee database using Oncomine. (**B**) Nine analyses were evaluated in comparing the RNA expression of *EGFR* between lung adenocarcinoma and normal tissue. Values above the average were considered *EGFR* over-expression (red). (**C**) Twelve analyses of *JUN* gene demonstrated the different expression in lung adenocarcinoma compared to normal tissue. Those below the average were considered *JUN* lower-expression (blue).

To investigate the clinical significance of the hub genes, the expression of these 10 hub genes in lung cancer and normal tissues was analyzed with the Oncomine database^26,50,51^. A previous study (Bhattacharjee)^52^ revealed that *EGFR* mRNA expression levels were significantly higher in squamous cell lung carcinoma than in lung (Fig. 12A). In addition, the expressions of hub genes in human lung adenocarcinoma were shown in Figures S27A–S35A, which suggested that the mRNA expression levels of *MMP9* and *NRAS* were significantly higher in lung adenocarcinoma than in normal lung tissue^52,53^.

In addition, the cDNA microarray analysis was also conducted using Oncomine database. As expected, *EGFR* (Fig. 12B), *ITGA2, KRAS, MMP9, NRAS* and *MAPK13* (Figures S28B, S32B–35B) had higher levels in lung adenocarcinoma than in normal lung tissues. However, three hub genes *JUN* (Fig. 12C), *FGF2, CCND1* and *IL6* (Figures S29B–31B) expressed fewer mRNAs in lung cancer tissues than normal lung tissues.

### Hub protein modeling

In order to further investigate the protein structure of these 10 hub genes, 3D protein structures of hub gene-corresponding proteins were modeled with the help of Modeller (9v20). The multi-template-based protein modeling method was used. In all hub proteins, except for the JUN, the 3D protein models of the other 9 could be established by homology modeling (Table S1). Based on the DOPE score, the best model of each hub protein with lowest score (EGFR: −81868.41406; ITGA2: −108323.50781; FGF2: −17431.5957; CCND1: −32457.32227; IL6: −21543.88477; KRAS: −20803.88086; MMP9: −70487.38281; NRAS: −20557.48828; MAPK13: −44303.85156) were selected for further analysis.

Due to unsatisfactory results from NCBI Blast alignment analysis, JUN 3D models were modeled *de novo* by using Rosetta Macromolecular Modeling software. In all 850 candidate models, the lowest scored model (No. S_00000242 which scored 43.301) was chosen as the best one for subsequent MD simulation study.

### Molecular dynamics and simulation

**Figure 13:**
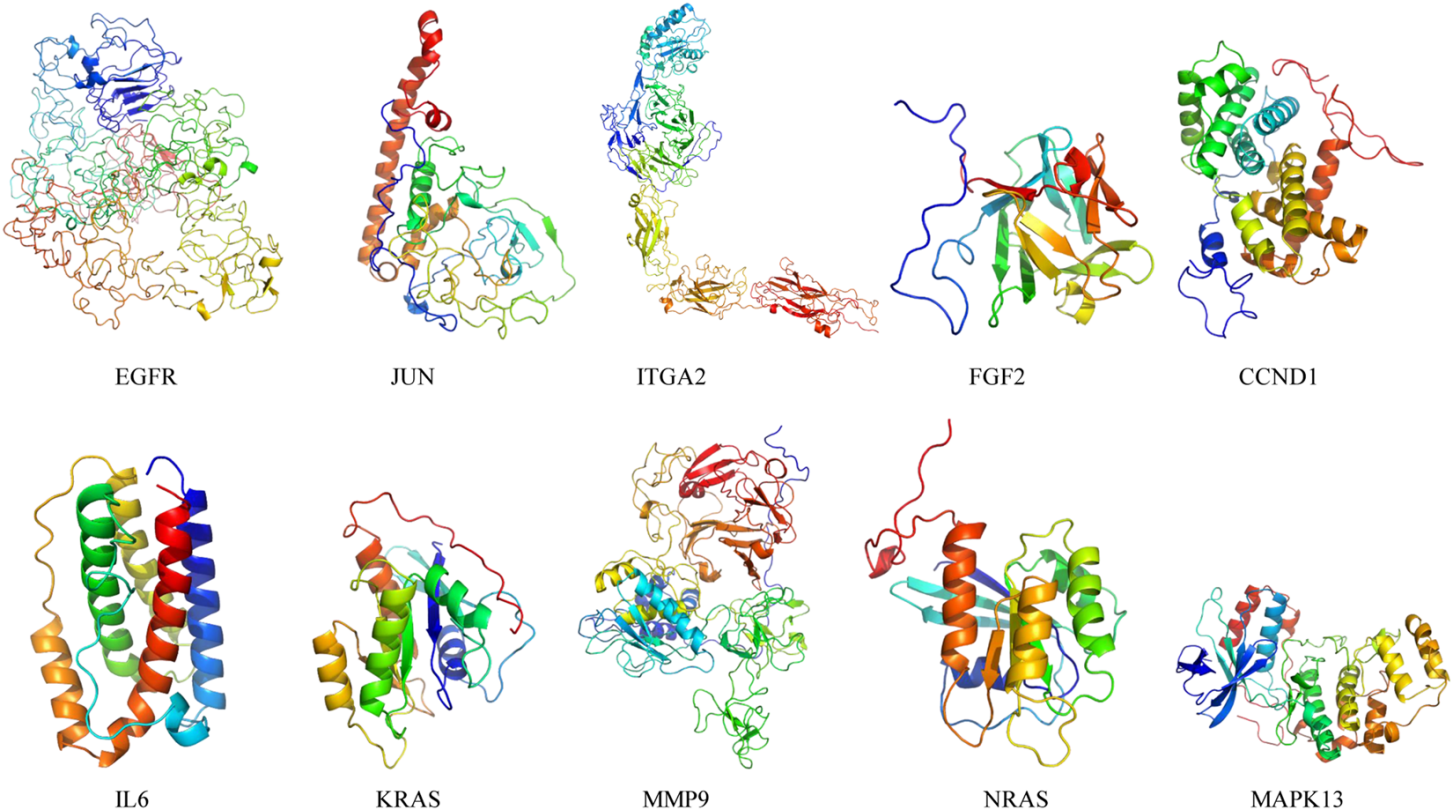
MD-refined 3D structure of the hub genes (drawn by PyMOL (www.pymol.org)).

**Figure 14:**
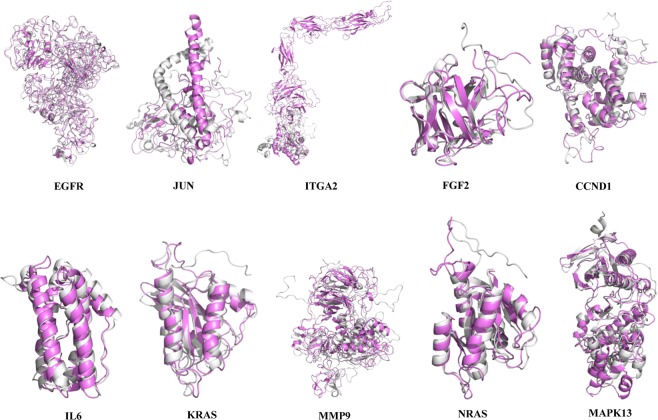
Superposition of the primarily modeled structure (gray) and the MD-refined protein structure (violet).

**Figure 15:**
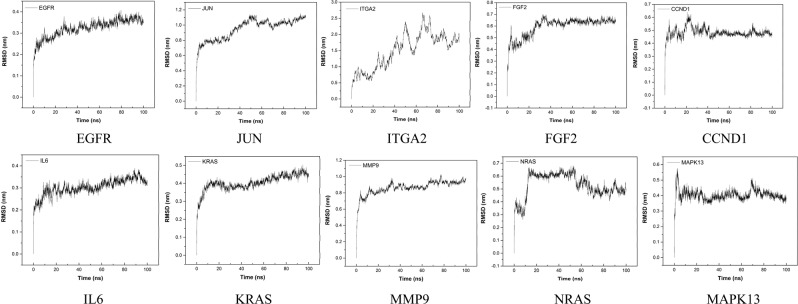
Molecular dynamics simulation of the hub genes (root mean square deviation (RMSD) with time (ns)).

**Figure 16:**
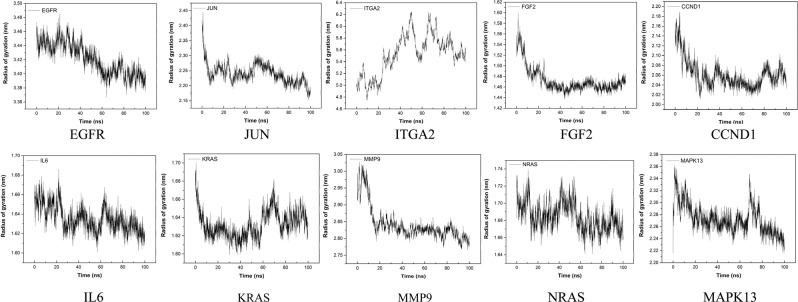
Radius of gyration of the hub genes.

To conduct energy minimization and to investigate the structural behavior of hub protein 3D models in the dynamic system, MD simulation was performed for 100 ns and analyzed using GROMACS utilities (Figs. 13–16). The structural convergence included RMSD (Fig. 15) and gyrate (Fig. 16).

As shown in Fig. 15, the analysis of backbone atoms demonstrated initial equilibration up to 10 ns (except for FGF2 13 ns, ITGA2 21 ns and CCND1 17 ns), and the structure started to converge after 11 ns (except for FGF2 14 ns, ITGA2 22 ns and CCND1 18 ns). Subsequently, RMSD value illustrated deviation up variously in the production MD run (EGFR: 25 ns, JUN: 50 ns, ITGA2: 70 ns, FGF2: 32 ns, CCND1: 30 ns, IL6: 20 ns, KRAS 40 ns, MMP9: 30 ns, NRAS: 65 ns and MAPK13: 70 ns). Finally, models showed stable conformation with an RMSD till the end the MD production run (EGFR: 0.30–0.35, JUN: 0.90–1.10, ITGA2: 5.4–5.8, FGF2: 0.60–0.65, CCND1: 0.44–0.52, IL6: 0.25–0.30, KRAS 0.35–0.45, MMP9: 0.80–0.90, NRAS: 0.45–0.50 and MAPK13: 0.35–0.40. Noteworthy, the simulation of larger proteins (EGFR 1157 aa, ITGA2 1181 aa) were performed by running MD 3 times to refine the protein structures (300 K (20 ns)−350 K (20 ns)−300 K(100 ns) in MD process).

The radius of gyration (Rg) showed the level of compactness present in the protein model. The reduction of Rg values indicated the stability of the system. As shown in Fig. 16 and Table S4, the average Rg value for hub gene proteins (EGFR: 3.42, JUN: 2.24, ITGA2: 5.53, FGF2: 1.47, CCND1: 2.06, IL6: 1.64, KRAS 1.63, MMP9: 2.84, NRAS: 1.69 and MAPK13: 2.27) for protein structures, with their maximum values denoted the stability of the protein structures. These data implied that the proteins of the hub genes had reached a balanced state in the simulation system.

**Figure 17:**
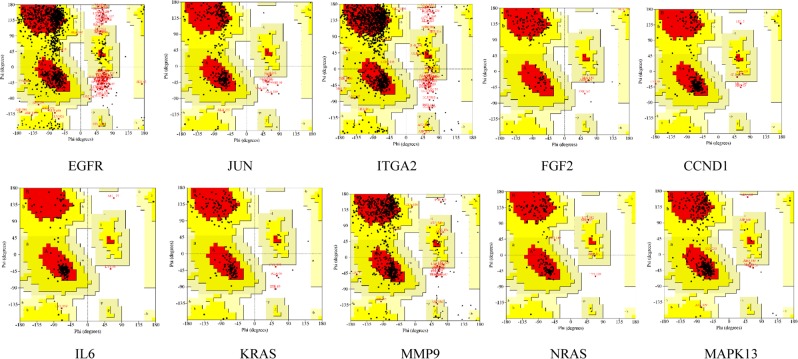
Ramachandran plot of hub proteins.

Ramachandran plot analysis (Table S5) were conducted to evaluate the lowest-potential frame of proteins during MD process. As shown in Fig. 17 and Table S5, the residues existed in outlier region ranged from 0% to 3.8% (EGFR 3.8%; JUN 2.2%; ITGA2 2.1%; FGF2 0.8%; CCND1 1.1%; IL6 1.3%; KRAS 1.8%; MMP9 1.4%; NRAS 0.6%; MAPK13 0.9%).

After the MD production run, the 3D models of hub gene proteins were shown in Fig. 14, with RMSD of primary models and MD-refined models (EGFR 2.364; JUN 9.221; ITGA2 17.898; FGF2 0.926; CCND1 2.467; IL6 1.620; KRAS 1.522; MMP9 5.583; NRAS 1.696 and MAPK13 2.051).

## Discussion

In this study, we acquired the data from GSE33845 and identified 251 genes out of a total of 50,802 genes using bioinformatics analysis. Function annotation showed that these DEGs were mainly involved in ECM-receptor interaction, pathways in cancer, MAPK signaling pathway and glutathione metabolism. By constructing the PPI, 10 hub genes were finally identified, followed by a series of comprehensive bioinformatics analysis. At last, by using three-dimensional modeling and molecular dynamics simulation (100 ns), the three-dimensional protein structure models of these hub genes were constructed.

### Lung cancer cells in dcEF-*hub genes*

In the present study, a total of 10 hub genes were obtained: *EGFR, JUN, ITGA2, FGF2, CCND1, IL6, KRAS, MMP9, NRAS* and *MAPK13*. In the following studies, a series of bioinformatics tools were used to explore these hub genes systematically.

### Lung cancer cells in dcEF-*hub genes-basic analysis*

*EGFR* is a member of the ErbB family of receptors, which exist on the cell surface. It was reported that electric field-enhanced directional migration related to the expression level of *EGFR* receptor. Many *EGFR* inhibitors have been developed that can stop the growth of tumors, and in some cases, make the tumors disappear^54^. These findings may provide useful help for the development of *EGFR*-related anticancer drugs.

The *JUN* belongs to the AP-1 transcription factor family and the *JUN* family proteins consist of c-Jun, JunB and JunD. c-Jun is involved in numerous cell activities, such as neuronal apoptosis, survival, inflammation and repair^55^.

Integrin alpha2 beta1 (α2 β1) plays an essential role in tumor metastasis, invasion and angiogenesis. The changes in the expression level of this receptor are often closely related to the clinical prognosis of some solid tumors, suggesting that this gene is highly associated with the development of tumors. Recently, the report suggests that *ITGA2* expression might be regulated by epigenetic mechanisms^56^.

*FGF2* is also known basic fibroblast growth factor (bFGF), which is a member of the fibroblast growth factor family. Previous studies show that the bFGF affects the migration, proliferation and survival of tumor cells. Moreover, it has been confirmed that *FGF2* participates in many malignant diseases, including myeloproliferative syndromes, lymphomas, lung cancer and so on. Very recently, *FGF2* is found to be inhibited by the PI3K/Akt signaling pathway, which implies that the PI3K/Akt pathway and *FGF2* might be potential therapeutic targets to treat tumor recurrence^57^.

*CCND1* belongs to the D-type cyclins family (which includes the cyclin D1, D2 and D3). In human cancer, *CCND1* is more commonly dysregulated than *CCND2* and *CCND3*. Interestingly, overexpression of cyclin D1 in cancer tissues is closely related to cancer genome instability and resistance of DNA-damaging cancer drugs^58^, implying a close correlation between *CCND1* and the tumor.

*IL6* is a potent proinflammatory cytokine with essential functions in the management of the immune system. *IL-6* as a protagonist can regulate the growth and differentiation of various malignancies. Till now, a large number of reports suggest that *IL-6* is associated with a range of diseases with an inflammatory background., such as bone metabolism, anemia of chronic diseases and multiple cancers^59^. Excitingly, antibody drugs against *IL-6* are being tried to treat a variety of tumors including lung cancer^60^.

*KRAS* is a member of the mammalian RAS gene family. *KRAS* proteins play an important role in human cancer, and the transforming protein can cause a variety of malignant tumors, including mucinous adenoma, lung adenocarcinoma and colorectal carcinoma^61^. New technologies insights into the signaling pathways of *KRAS* with self-targeting have advanced renewed efforts to develop anticancer drugs. At present, *KRAS* is attracting more and more attention as a hot target in the field of anti-tumor drugs. In this study, we found that *KRAS* is one of the hug genes in lung cancer cells after dcEF treatment, suggesting that *KRAS* may be one of the targets of electrochemical therapy for tumors^62^.

*MMP9* belongs to the matrix metalloproteinase (MMP) family. Matrix metalloproteinases (MMPs) are a group of enzymes, which play a key role in the progression and transformation of tumors, such as cancer cell invasion and metastasis. Previous studies suggest that many members of the *MMP9* family (MMP1, 2, 3, 7, 9, 13 and 14) are closely related to multiple aspects of the tumor, including tumor metastasis, progression and prognoses^63^. Especially, *MMP9* drives metastasis and tumor progression. In the present study, *MMP9* is a response signal molecule of lung cancer cells to dcEF stimulation, suggesting that *MMP9* may also be involved in external physical stimulation feedback of lung cancer cells, such as dcEF.

*MAPK13* is a member of the mitogen-activated protein (MAP) kinase family. As an integration point for multiple biochemical signals, MAP kinases are involved in cell transcription, proliferation, development, differentiation and apoptosis. It seems that MAP kinase pathways represent a new kind of potential target for cancer therapy^64^.

Mutations in *NRAS* are often found in several hematological diseases and tumors, such as follicular thyroid cancer, somatic rectal cancer and myelomonocytic leukemia^65^. Our data show that NRAS is also involved in the response of lung cancer cells to external electric field stimulation, suggesting that *NRAS* may also anticipate in the electrochemical treatment of tumors.

### Lung cancer cells in dcEF-*hub genes-signaling pathway*

To explore the signaling pathways of these hub genes, a series of tools were utilized for subsequent study. A total of two signaling pathways were obtained: the FoxO signaling pathway and the AGE-RAGE signaling pathway.

The forkhead box O (FOXO) signaling pathway belongs to a family of transcription factors, which modulates the expression of genes in multiple cellular physiological processes including cell-cycle control, oxidative stress resistance, apoptosis and longevity. Recently, the FoxO signaling pathway is found to promote inflammation via enhancing Tlr4-mediated signaling in macrophages^66^. In this study, FoxO is involved in the stimuli response of lung cancer cells to electric fields, suggesting that this inflammatory process may also be stimulated by the electric field.

Previous studies had confirmed that RAGE expression had been detected in a variety of human tumors, including lung, colon, brain, colorectal, prostate, breast, ovarian cancer and oral squamous cell^67^. Moreover, increased expression of RAGE was documented in a variety of chronic inflammatory and acute diseases, such as chronic renal disease, vasculitis, osteoarthritis and late diabetic complications^68^. In our study, RAGE is also involved in the feedback of lung cancer cells in response to dcEF, indicating that direct current electric field stimulation may also cause changes in the inflammatory environment of tumor cells.

Overall, in the feedback of lung cancer cells to dcEF stimulation, two inflammation-related signaling pathways are activated, suggesting that changes in the inflammatory state of tumor cells may be involved in the feedback mechanism of tumor cells in response to electric field stimulation.

### Lung cancer cells in dcEF-*hub genes-survival analysis*

In this study, we computed Kaplan–Meier plots for the markers (*EGFR, JUN, ITGA2, FGF2, CCND1, IL6, KRAS, MMP9, NRAS* and *MAPK13*) to assess their effects on prognosis, which hinted that the prognostic value of *JUN, FGF2, IL6* and *MAPK13* had a high predictive power. However, the results of *EGFR, CCND1, ITGA2, KRAS, MMP9* and *NRAS* were (although partially significant) not convincing. Taken together, these results may enable us to deliver higher accuracy in prognosis prediction.

### Lung cancer cells in dcEF-*EGFR and electrotaxis*

Electrotaxis is a phenomenon in which cells can directionally respond to an applied electric field^69^. The cellular mechanisms of electrotaxis include a variety of factors such as the movement of intracellular/extracellular calcium ions, actin polymerization/depolymerization, actomyosin contractility, changes in cell surface charge, growth factors and protein kinases. In addition, the electrotaxis of cells is also closely related to metastatic disease, which provides a clue for potential clinical applications of cell electrotaxis^13^.

Among the many factors affecting cell electrotaxis, EGFR is considered to have a fundamental role in cells’ response to physical electric field stimulation. As a key protein in tumor growth and metastasis, EGFR is involved in the metastasis of many types of tumors^70^. However, in terms of cell electrotaxis, EGFR has shown opposite effects in different research systems. On the one hand, some studies suggest that EGFR may not participate in the cell’s electrotaxis. In 2013, by using a dcEF stimulation device made by researchers, the involvement of EGFR in the molecular signaling mechanism of human lung tumor cells’ response to electric fields is systematically investigated. After dcEF treatment (dcEF: 300 mV/mm, 2 h), the electrotaxis of CL1-5 cells is serum-dependent and EGFR-dependent. Moreover, after the EGFR signaling pathway is blocked by anti-EGFR monoclonal antibodies, the electrotaxis of CL1-5 cells is not affected, although higher levels of EGFR protein are expressed on the cells. Further molecular mechanism studies demonstrate that the modification of protein active sites caused by dcEF stimulation (physical stimulation) and EGF stimulation (biological stimulation) on CL1 cells is different (including phosphorylation of S6 ribosomal protein (rpS6) and Akt), which indicates that the response of CL1 cells to dcEF stimulation is not achieved through the EGFR signaling pathway^4^.

Similar results are also seen in two reports in 2017. After stimulation with dcEF (dcEF: 50–300 mV/mm, 4 h), mesenchymal stem cells (MSC) from C57BL/6 mice move toward the cathode. However, although the accumulation of EGFR is observed at the cathode MSCs, EGFR is not involved in electrotaxis^71^. In the same line, after dcEF stimulation (dcEF: 0–450 mV/mm, 0–8 h), lung adenocarcinoma H1975 cells move toward the cathode, and EGFR is related to cell migration without electric field stimulation rather than cell movement with electric field stimulation^72^. Overall, the above findings suggest that EGFR would not participate in the electrotaxis of human lung adenocarcinoma cells (H1975 and CL1-5) and mouse mesenchymal stem cells (MSC).

On the other hand, EGFR is involved in the electrotaxis of certain tumor cells. Under the stimulation of dcEF (dcEF: 150 mV/mm, 1 h), human metastatic breast cancer cells MDA-MB-231 move to the anode, accompanied by EGFR polarization, which suggests that EGFR is involved in the electrotaxis of MDA-MB-231 cells^73^.

To date, there are no conclusive explanations for the inconsistency of the effects of EGFR on the electrotaxis of various cell types^74^. We speculate that it may be caused by the following reasons.

1. The electric field affects different ion channels of different tumor cells, which may cause EGFR to play different roles when EF stimulates various types of tumor cells. For details on the response of ion channels to EF, see “4.5 RAW 264.7 cells in dcEF-ion channels” below.
2. The differences in the chemical environment of the cells in each experimental system may lead to different experimental results. At present, most researchers build their own electric field devices to introduce electric field stimulation to cells. It is worth noting that different electrodes cause different chemical environments in which cells are placed. Experimental systems using platinum electrodes^72,73^ and Ag-AgCl electrodes^3,4,6^ can cause differences in the chemical environment in which cells are placed. Considering that the electrolysis of aqueous buffer using platinum electrodes decreases the pH at the positive electrode and decreases the pH at the negative electrode^75^. Therefore, the resulting pH gradient across the cells under these studies may also contribute to the different dcEF effects.
3. The heterogeneity of tumor cells causes EGFR to participate in different manifestations of cells in response to EF stimulation. Different tumor types have a high degree of heterogeneity^25^, which may cause EGFR of different tissue types to act differently on electrotaxis.

### Lung cancer cells in dcEF-*MMP inhibitor and electrotaxis*

The MMP family is involved in electric field-induced tumor cell migration. Very recently, Ji-Yen Cheng’s group conducts a study on a non-specific MMP inhibitor regulating the electrotaxis of tumor cells^6^. Three cell types are used in the study, of which human lung squamous cell carcinoma NCI-H520 cells and human large cell lung carcinoma NCI-H460 are used to represent non-small cell lung cancer (NSCLC) cells, and non-cancerous MRC- 5 lung fibroblasts are considered normal cell controls. After dcEF stimulation (dcEF: 300–375 mV/mm, 2–6 h), H460 and H520 cells migrate toward the cathode, while MRC-5 cells move toward the anode. Moreover, these cells move faster as the electric field strength increases, suggesting that the electric field strength is a regulator of cell migration. Interestingly, the MMP inhibitor doxycycline^76^ enhances dcEF-induced migration but inhibits H520 and H460 migration to the cathode, suggesting that the MMP inhibitor (doxycycline) regulates the NSCLC’s electrotaxis.

In the same line, in our study, electric field stimulation (dcEF: 300 mV/mm, 2 h) caused significant changes in the MMP9 gene (which belongs to the MMP family) of lung cancer CL1-0 cells, indicating that MMP9 should be an electric field-sensitive gene. Taken together, the MMP family (including MMP9) may be a bridge connecting the external physical electric field stimulation and the response of lung cancer cells.

### Lung cancer cells in dcEF-*CL1-0 and electrotaxis*

Since electrotaxis was reported, it has attracted a lot of attention^13,69^. However, for cells whose electrotaxis is not obvious, less attention has been paid to its underlying molecular mechanisms such as gene expression and molecular mechanisms. In this study, human lung adenocarcinoma CL1-0 cells, which are less malignant than lung adenocarcinoma CL1-5 cells, were selected as the cell model with insignificant electrotaxis for research.

A series of studies demonstrate that the electrotaxis of human lung adenocarcinoma CL1-5 cells with high malignancy is prominent, in contrast, the electrotaxis of human lung adenocarcinoma CL1-0 cells with low malignancy is not significant^6,10,77^. Under EF stimulation, some tumor cells undergo redirection and migration, which has potential implications for tumor metastasis^13,73,78^. However, for tumor cells with insignificant electrotaxis, their potential signaling pathways are still worthy of attention. Our data suggest that the inflammatory state of human lung adenocarcinoma CL1-0 cells may be involved in the feedback mechanism of lung cancer cells in response to electric field stimulation, which provides preliminary data for exploring the signaling mechanism of tumor cells that are not significantly electrotaxis.

### RAW 264.7 cells in dcEF-*ion channels*

Electric field stimulation can significantly affect the cell’s ion channels. Among the many cellular ion channels affected by EF, the effect of EF on voltage-gated calcium ion channels has been studied intensively.

EF mainly affects voltage-gated calcium channel proteins. On U87 glioblastoma cells, pulsed electric fields (PEF) directly act on the cell’s voltage-gated ion channels, which can be blocked by pharmacological antagonists of ion channels. Moreover, the cell membrane depolarization caused by EF can also be blocked by certain cationic channels^79^. Therefore, ion channels may be one of the main bridges that EF acts on cells. In addition, data from molecular dynamics simulations show that four phenylalanine residues at the internal gate region on human calcium channel proteins are important for EF to activate ion channels^80^. In summary, ion channels may be one of the main bridges that EF acts on cells, which deserves further investigation.

### Lung cancer cells in dcEF-*reactive oxygen species*

Increasing evidence suggests that reactive oxygen species (ROS) may be one of the bridges connecting electric fields and inflammatory signaling pathways. On the one hand, ROS is one of the key signal molecules for the electric field to regulate cell behavior. After dcEF stimulation (dcEF: 200 mV/mm, 30–60 min), glioma cells (C6, 87 and U251) migrated directionally, accompanied by a significant increase in intracellular ROS levels^81^. Besides, electric field stimulation (dcEF: 100–400 mV/mm, 2 h) on NIH 3T3 fibroblasts not only caused cell migration but also increased intracellular ROS production in an EF intensity-dependent manner^82^. On the other hand, ROS is also one of the important signaling molecules in the inflammatory signaling pathway. At the site of inflammation, polymorphonuclear neutrophils (PMNs) produce excess ROS, which can cause damage to endothelial cells and tissues. At the single-cell level, ROS can be produced in cellular compartments such as mitochondria, endoplasmic reticulum, peroxisomes and cytoplasm, and are widely involved in the general inflammatory signaling cascade^83^. According to the above findings, ROS is not only a sensitive molecule in which the electric field affects cells, but also one of the key members in the inflammatory response cascade. Therefore, the ROS system may be one of the bridges connecting cells (biological systems) and electric fields (physical stimuli), which deserves further exploration.

### The limitations of our study

There were still a few limitations in the present study. Firstly, only one lung cancer tumor cell line (CL1-0) was analyzed in silico for changes in gene expression profiles caused by dcEF stimulation, which has limitations on the response of the entire lung cancer tissue to dcEF. Considering the complexity of lung cancer classification^25^, it should be clearly realized that this study only has certain reference value for the response of lung cancer tissues to dcEF stimulation. The response of lung cancer tissue to electric field stimulation and its specific molecular mechanism needs to be further improved with more types of pathological tissue and electric field stimulation.

Secondly, in silico data has limited reference value for real clinical research, which must be experimentally verified. In this study, we had difficulties in obtaining original experimental samples and constructing professional electric field stimulation devices. Therefore, further biological experimental verification could not be performed. For example, in a physiological experiment using adult rat lungs, the alveolar transepithelial potential difference was observed by a unique method for isolating intact alveolar epithelial cells from parallel pulmonary epithelia^84^. This type of research provides a solid physiological basis for investigating the mechanism by which electric fields affect cells. As an in silico study, from the perspective of bioinformatics, we tried to explore the changes in gene expression profiles of human lung cancer CL1-0 cells caused by electric field stimulation (dcEF: 300 mV/mm, 2 h), and to investigate the molecular mechanisms of lung cancer cells (biological systems) in response to electric field stimuli (physical stimuli).

Thirdly, changes in the expression level of the hub gene lack validation of qPCR results. Since the invention of microarray technology, the stability and accuracy of gene expression have been continuously improved. However, qPCR verification of chip results has become the standard procedure for microarray experiments. In our work, we had difficulty obtaining the original samples and hence deliberately chose the genes with larger changes for analysis. In addition, in the experimental stage, the authors have already verified the expression of some differential genes by qPCR, which showed that the microarray results and qPCR results have a good consistency.

Finally, the differences in analysis results between different databases can easily cause some confusion. According to the results of Kaplan Meier analysis, the survival rates of the patients expressing high levels of *JUN, FGF2, IL6* and *MAPK13* were higher than that of the low-expressing patients, whereas the patients expressing high levels of *EGFR, CCND1, ITGA2, KRAS, MMP9* and *NRAS* showed no significance in comparison with the low-expressing groups. As for the differences between the results of the above different database analyses, we speculated that these differences may be explained by the following reasons: Firstly, the level of gene expression did not directly reflect their protein levels, as a series of modulations could also affect the protein production, including transcription, post-transcription, translation and post-translation. Therefore, the level of gene expression may not directly represent the activity of its corresponding protein. Second, the expression level of each gene was affected by multiple regulatory networks. Hence, the short-term or long-term interaction between these regulatory networks determined the final gene expression level, resulting in large differences in gene expression levels. Thirdly, the survival curve was the embodiment of the outcome of the tumor and a composite result of multiple genes during tumorigenesis, hence the level of gene expression was just one of the many factors that affected tumor survival time. Finally, there were differences between the samples collected from different databases. Besides, these differences may also represent the diversity of tumor biology.

## Conclusions

In the present study, three levels of research have been integrated with an attempt to provide clues for understanding the molecular mechanisms by which dcEF stimulates tumor cells. At the gene expression level, with the help of bioinformatics, DEGs after dcEF stimulation of lung cancer cells were obtained, and then the hub genes were obtained by functional annotation analysis. At the protein structure level, the optimal structure of the proteins corresponding to the hub genes was obtained through molecular dynamics simulations of at least 100 ns, which provided meaningful data for a deeper understanding of the effect of external physical electric fields on the protein structure of tumor cells. At the clinical significance level, through the analysis of various oncology gene expression databases, the expression characteristics of hub genes in human tumor tissues and their guiding value for tumor prognosis were clarified. Collectively, our work would not only help to understand the mechanism of dcEF-induced changes in tumor cells, but also be useful in pointing out that the inflammatory state of tumor cells may be involved in the feedback of the cells to the dcEF stimulation.

## Supplementary information

Supplementary information
