# Supplementary material for: Cellular processes involved in lung cancer cells exposed to direct current electric field
Source: Sci Rep. 2020 Mar 24;10:5289. doi: 10.1038/s41598-020-62332-0 (PMC7093422; doi:10.1038/s41598-020-62332-0)
Supplement: Supplementary file 1 — Supplementary information [file 41598_2020_62332_MOESM1_ESM.pdf]

Supplementary Information

Cellular processes involved in lung cancer cells exposed to direct current electric field

Huijuan Li [1], Shibin Liu [1,*], XueYang [1], Yongqian Du [1], Jiezhang Luo [1], Jie Tan [1], and Yulong Sun [2,*]

Affiliations:

[1.] School of Electronics and Information, Northwestern Polytechnical University, Xi'an, 710072, China

[2] School of Life Sciences, Northwestern Polytechnical University, Xi'an, 710072, China

Emails:

*Corresponding authors:

Shibin Liu, Ph.D.

Address: School of Electronics and Information, Northwestern Polytechnical University, 127 Youyi Xilu, Xi' an Shaan Xi Province, 710072, PR China

Tel.: +86 29 88491653, E-mail: liushibin@nwpu.edu.cn

Yulong Sun, Ph.D.

Address: School of Life Sciences, Northwestern Polytechnical University, 127 Youyi Xilu, Xi' an Shaan Xi Province, 710072, PR China

Tel.: +86 29 88460332, E-mail: yulongsun@nwpu.edu.cn

Table of contents

1. **Figures**

2. **Tables**

3. **Supplementary References**

A (1)

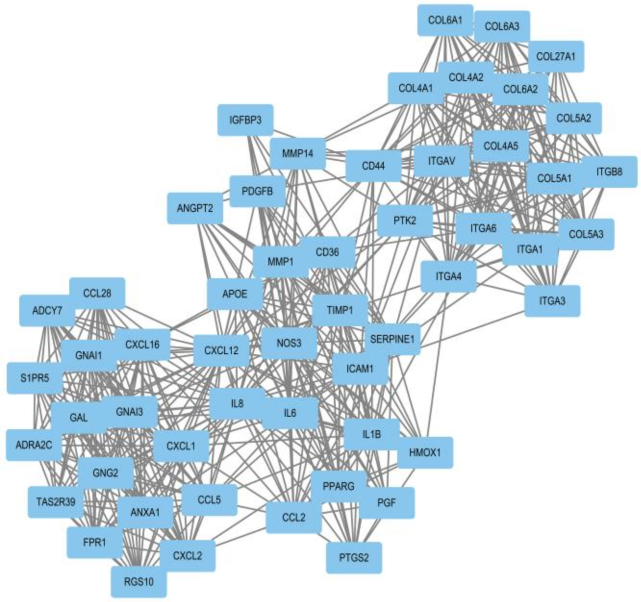

A (2)

GeneSet	P-value	Correct p-value	Nodes
extracellular region part	2.23E-25	4.09E-22	*SERPINE1 CXCL1 CXCL2 CXCL16 ICAM1 CCL5 CCL2 HMOX1 TIMP1 APOE COL27A1 ANGPT2 MMP1 IGFBP3 PGF MMP14 IL6 CXCL12 COL4A2 COL5A1 IL8 COL 4A1 IL1B COL6A2 COL5A3 COL6A1 COL5A2 COL6A3 COL4A5 ITGA6 CCL28*
locomotion	2.78E-20	2.55E-17	*ITGA4 NOS3 ITGA1 PDGFB FPR1 CXCL1 CXCL2 PTK2 CXCL16 ICAM1 MMP14 IL6 CXCL12 COL5A1 IL8 IL1B CCL5 CCL2 ITGA6 CCL28 CD44*
extracellular region	2.12E-19	1.29E-16	*SERPINE1 PDGFB CXCL1 CXCL2 CXCL16 ICAM1 CCL5 CCL2 HMOX1 TIMP1 APOE COL27A1 ANXA1 ANGPT2 MMP1 IGFBP3 PGF MMP14 IL6 GAL CXCL12 COL4A2 COL5A1 IL8 COL4A1*

			IL1B COL6A2 COL5A3 COL6A1 COL5A2 COL6A3 COL4A5 ITGA6 CCL28

B (1)

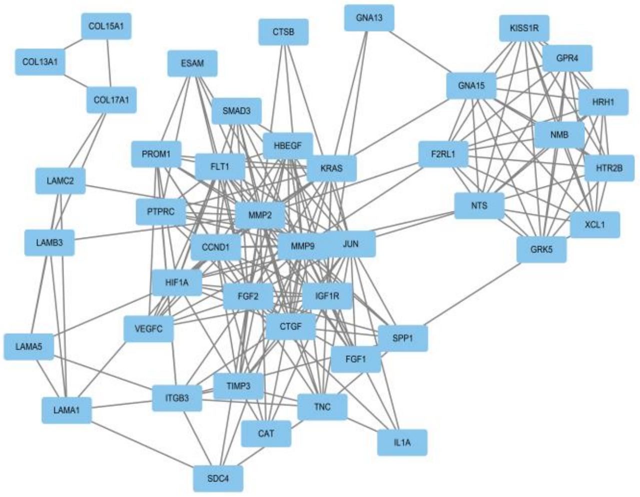

B (2)

GeneSet	P-value	Correct p-value	Nodes
positive regulation of cell development	1.91E-19	3.51E-16	*COL17A1 LAMA5 COL15A1 FLT1 COL13A1 LAMA1 ITGB3 TNC HTR2B LAMC2 FGF1 HIF1A FGF2 IGF1R CTGF GNA13 CCND1 SPP1 TIMP3 CTSB NMB JUN SMAD3 LAMB3 MMP2 VEGFC MMP9 PTPRC HBEGF*
protein metabolic process	3.28E-18	3.01E-15	*COL17A1 LAMA5 COL15A1 FLT1 SDC4 COL13A1 LAMA1 ITGB3 TNC HTR2B LAMC2 FGF1 NTS HIF1A FGF2 IGF1R CTGF GNA13 CCND1 SPP1 TIMP3 CTSB NMB JUN SMAD3 LAMB3 MMP2 VEGFC MMP9 PTPRC KRAS HBEGF*

neurogenesis	6.43E-17	3.93E-14	*LAMA5 NMB JUN FLT1 SMAD3 LAMA1 ITGB3 HTR2B GPR4 FGF1 FGF2 IGF1R CTGF GNA13 IL1A HRH1 GNA15 PTPRC CCND1 GRK5 KISS1R F2RL1 KRAS HBEGF*

C (3)

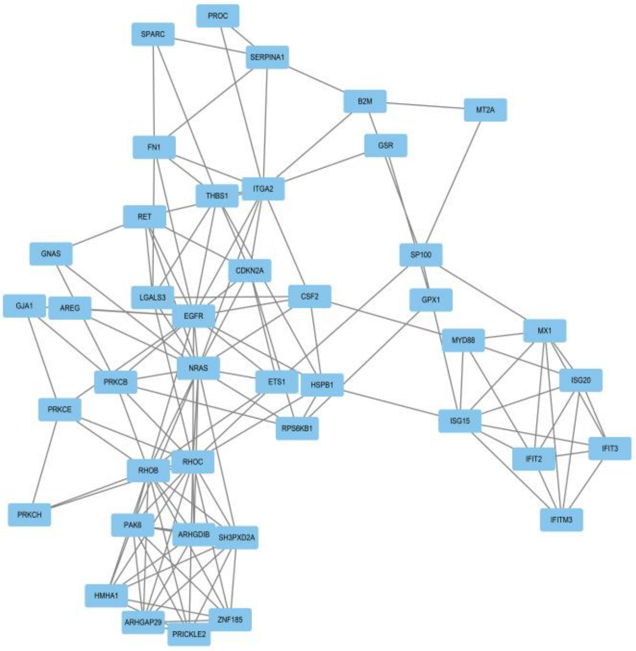

C (2)

GeneSet	P-value	Correct p-value	Nodes
positive regulation of cell communication	2.73E-09	2.50E-06	*NRAS GJA1 CSF2 GPX1 CDKN2A PRKCB ITGA2 RHOC THBS1 EGFR MYD88*
intracellular signaling pathway	4.66E-09	2.50E-06	*RET SP100 PRKCH CDKN2A PRKCE ARHGAP29 HMHA1 RHOC THBS1 EGFR RHOB NRAS RPS6KB1 ARHGDIB GNAS MYD88*
regulation of apoptosis	6.43E-09	2.50E-06	*CSF2 GPX1 CDKN2A PRKCE MX1 HSPB1 THBS1 ETS1 EGFR NRAS ARC PROC RPS6KB1 MYD88*

D (1)

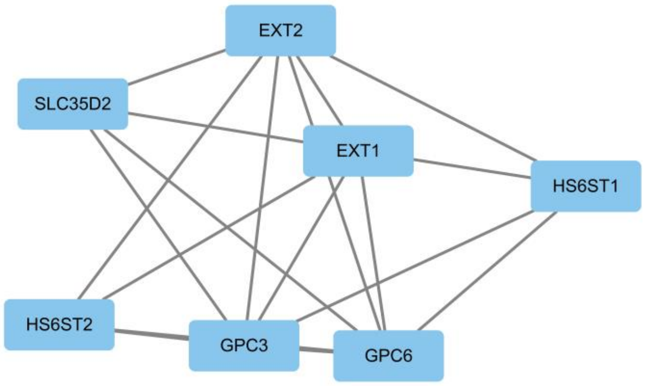

D (2)

GeneSet	P-value	Correct p-value	Nodes
heparan sulfate proteoglycan biosynthetic process	4.47E-09	1.08E-06	*EXT1 EXT2 HS6ST1*
heparan sulfate proteoglycan metabolic process	1.06E-08	1.28E-06	*EXT1 EXT2 HS6ST1*
carbohydrate biosynthetic process	5.32E-08	4.27E-06	*EXT1 EXT2 HS6ST1 HS6ST2*

E (1)

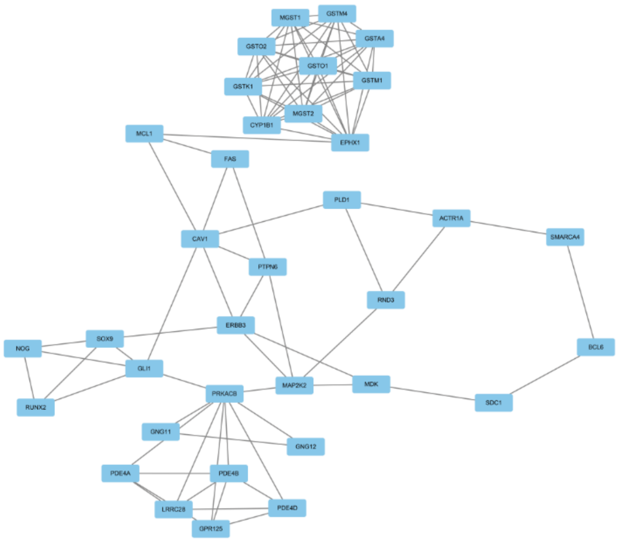

E (2)

GeneSet	P-value	Correct p-value	Nodes
glutathione transferase activity	1.90E-17	2.45E-14	*GSTM4 GSTK1 GSTM1 GSTO2 GSTA4 GSTO1 MGST1 MGST2*
transferase activity, transferring alkyl or aryl (other than methyl) groups	4.04E-14	2.61E-11	*GSTM4 GSTK1 GSTM1 GSTO2 GSTA4 GSTO1 MGST1 MGST2*
response to organic substance	5.03E-07	2.17E-04	*ERBB3 CAV1 EPHX1 MGST1 CYP1B1 SDC1 PLD1 GNG12 PRKACB GNG11 MCL1*

F (1)

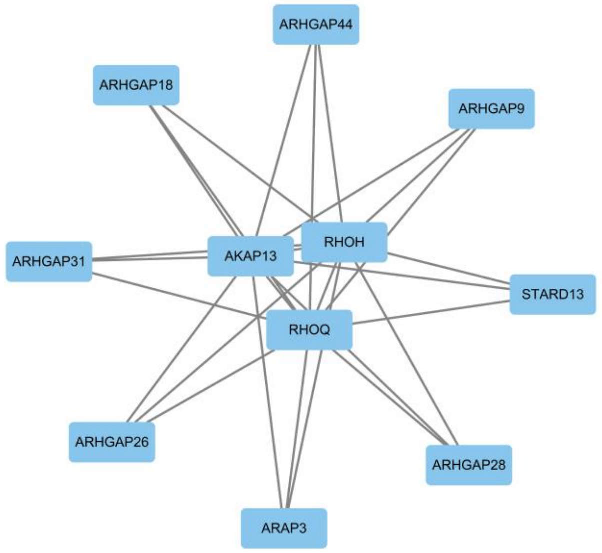

F (2)

GeneSet	P-value	Correct p-value	Nodes
GIPase regulator activity	1.88E-14	3.81E-12	*ARHGAP9 AKAP13 ARHGAP31 STARD13 RHOH ARAP3 ARHGAP18 ARHGAP28 ARHGAP26*
nucleoside-triphosphatase regulate activity	2.28E-14	3.81E-12	*ARHGAP9 AKAP13 ARHGAP31 STARD13 RHOH ARAP3 ARHGAP18 ARHGAP28 ARHGAP26*
GIPase activator activity	6.20E-12	6.91E-10	*ARHGAP9 ARHGAP31 STARD13 ARAP3 ARHGAP18 ARHGAP28 ARHGAP26*

G (1)

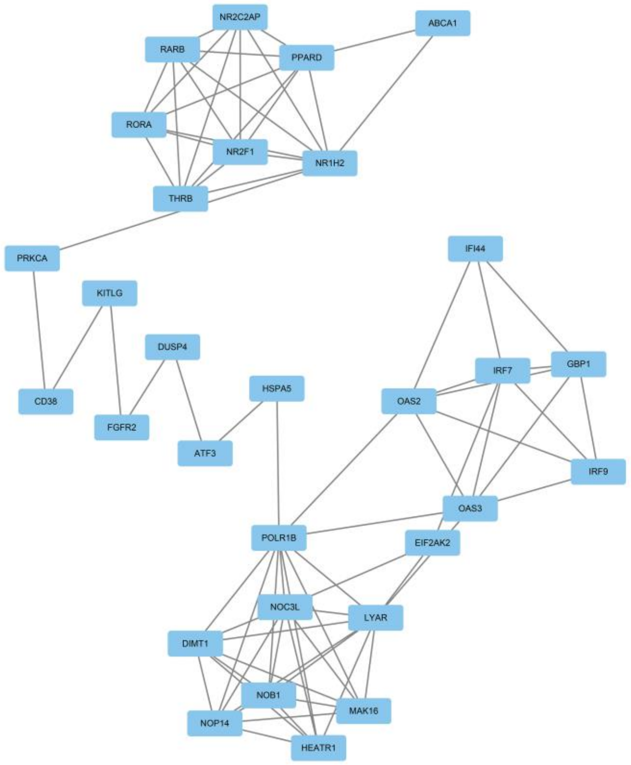

G (2)

GeneSet	P-value	Correct p-value	Nodes
steroid hormone receptor activity	1.80E-10	1.44E-07	*THRB NR1H2 NR2F1 RARB RORA PPARD*
ligand-dependent nuclear receptor activity	2.60E-10	1.44E-07	*THRB NR1H2 NR2F1 RARB RORA PPARD*
nucleus	1.14E-08	4.20E-06	*DUSP4 NOP14 THRB HSPA5 DIMT1 NR1H2 MAK16 HEATR1 NR2F1 RORA PRKCA NOC3L NOB1 OAS2 POLR1B IRF7 CD38 RARB LYAR FGFR2 ATF3 IRF9 NR2C2AP PPARD*

H (1)

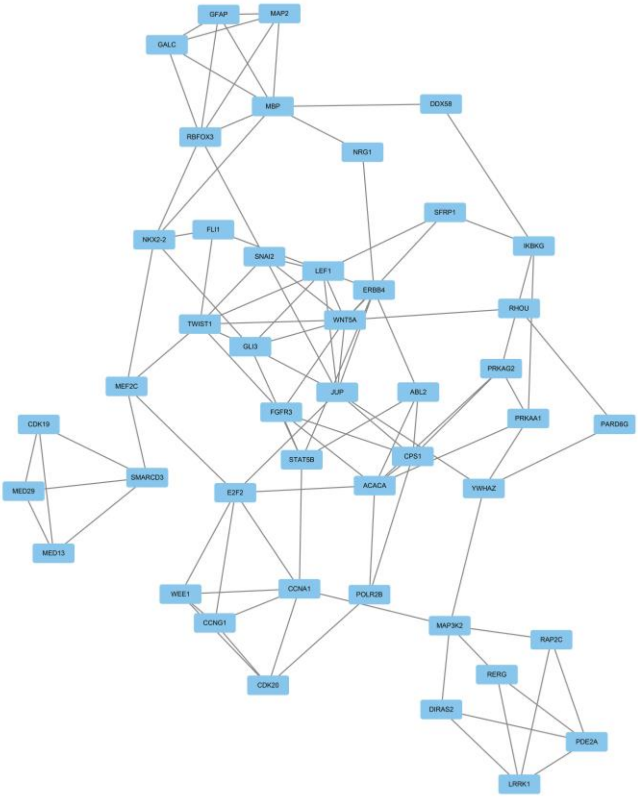

H (2)

GeneSet	P-value	Correct p-value	Nodes
signaling	5.50E-09	4.68E-06	*PRKAA1 DIRAS2 LEF1 LRRK1 PRKAG2 RERG GLI3 MED13 ERBB4 ABL2 MBP IKBKG NKX2-2 MAP3K2 STAT5B PDE2A WNT5A NRG1 YWHAZ RAP2C SFRP1 CCNG1 SNAI2 RHOU FGFR3*
biological regulation	7.32E-09	4.68E-06	*PRKAA1 SMARCD3 DIRAS2 LEF1 LRRK1 PRKAG2 TWIST1 RERG GLI3 ACACA MED13 ERBB4 MAP2 ABL2 E2F2 MBP IKBKG NKX2-2 MAP3K2 STAT5B MEF2C JUP DDX58 PDE2A WNT5A NRG1 YWHAZ FLI1 MED29 RAP2C SFRP1 CPS1 CCNG1 SNAI2 RHOU FGFR3*
regulation of biological process	8.40E-09	4.68E-06	*PRKAA1 SMARCD3 DIRAS2 LEF1 LRRK1 PRKAG2 TWIST1 RERG GLI3 MED13 ERBB4 MAP2 ABL2 E2F2 MBP IKBKG NKX2-2 MAP3K2 STAT5B MEF2C JUP DDX58 PDE2A WNT5A NRG1 YWHAZ FLI1 MED29 RAP2C SFRP1 CPS1 CCNG1 SNAI2*

RHOU FGFR3

I (1)

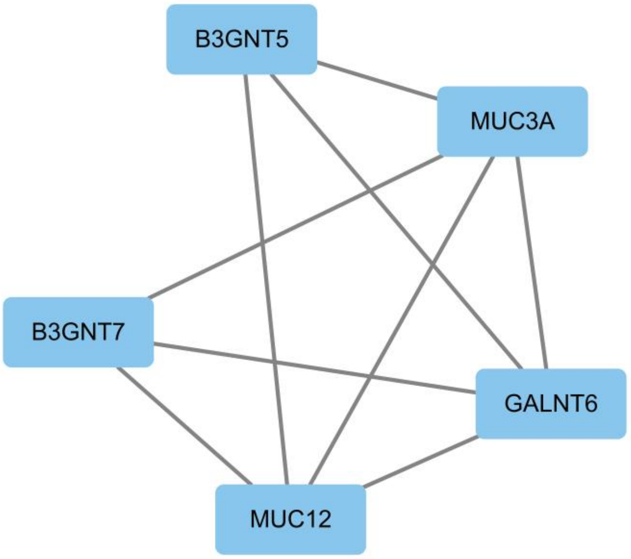

I (2)

GeneSet	P-value	Correct p-value	Nodes
protein amino acid glycosylation	2.89E-06	9.63E-05	*GALNT6 B3GNT7 B3GNT5*
macromolecule glycosylation	2.89E-06	9.63E-05	*GALNT6 B3GNT7 B3GNT5*
glycosylation	2.89E-06	9.63E-05	*GALNT6 B3GNT7 B3GNT5*

J (1)

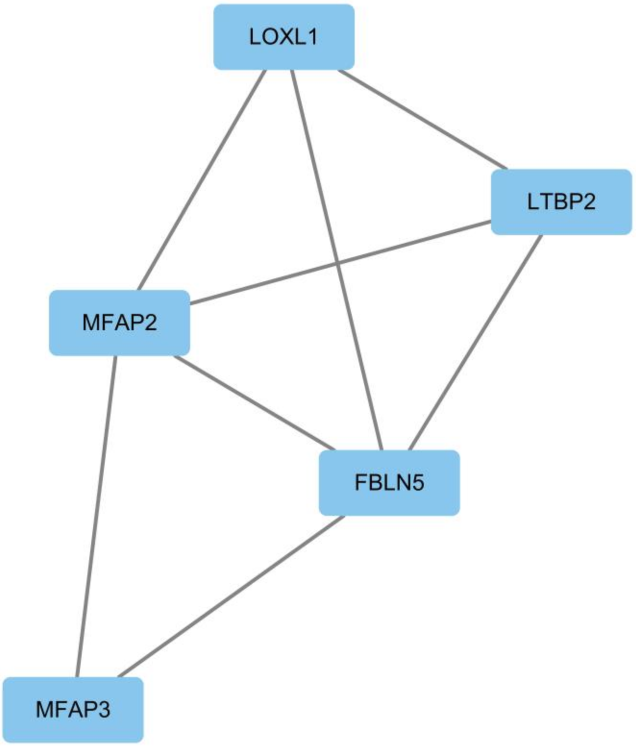

J (2)

GeneSet	P-value	Correct p-value	Nodes
proteinaceous extracellular matrix	4.58E-07	2.62E-05	*MFAP2 LTBP2 LOXL1 FBLN5*
extracellular matrix	6.46E-07	2.62E-05	*MFAP2 LTBP2 LOXL1 FBLN5*
extracellular region part	4.47E-05	1.21E-03	*MFAP2 LTBP2 LOXL1 FBLN5*

K (1)

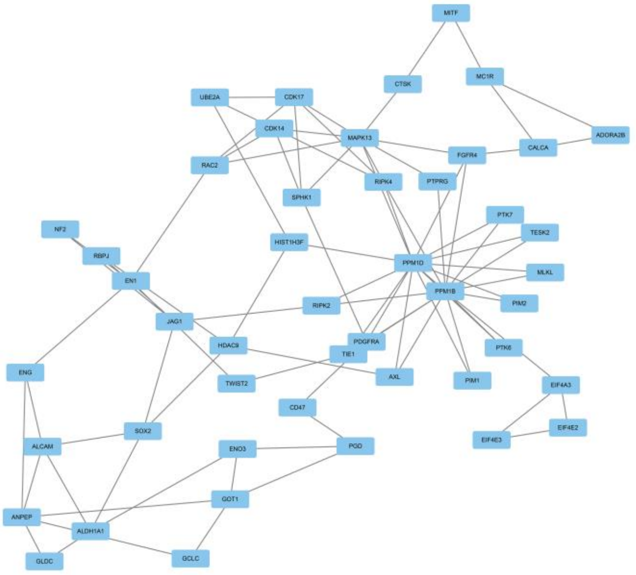

K (2)

GeneSet	P-value	Correct p-value	Nodes
protein kinase activity	5.71E-13	3.88E-10	*CDK17 PDGFRA MLKL RIPK2 TIE1 RIPK4 TESK2 PTK6 MAPK13 PTK7 AXL PIM1 PIM2 FGFR4 CDK14 ENG*
phosphotransferase activity, alcohol group as acceptor	6.29E-13	3.88E-10	*CDK17 PDGFRA MLKL RIPK2 TIE1 SPHK1 RIPK4 TESK2 PTK6 MAPK13 PTK7 AXL PIM1 PIM2 FGFR4 CDK14 ENG*
phosphorus metabolic process	9.24E-13	3.88E-10	*CDK17 PDGFRA MLKL RIPK2 TIE1 RIPK4 TESK2 PTK6 PPM1D PTPRG MAPK13 PPM1B ADORA2B PTK7 AXL PIM1 PIM2 FGFR4 CDK14*

L (1)

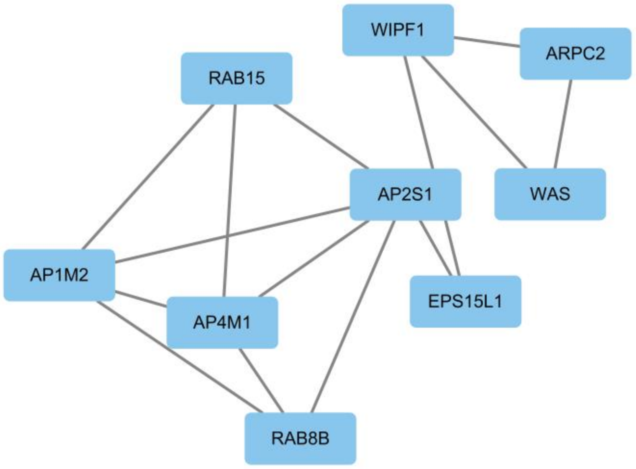

L (2)

GeneSet	P-value	Correct p-value	Nodes
clathrin adaptor complex	2.60E-07	3.51E-05	*AP4M1 AP2S1 AP1M2*
AP-type membrane coat adaptor complex	2.91E-07	3.51E-05	*AP4M1 AP2S1 AP1M2*
clathrin coat	6.34E-07	5.09E-05	*AP4M1 AP2S1 AP1M2*

M (1)

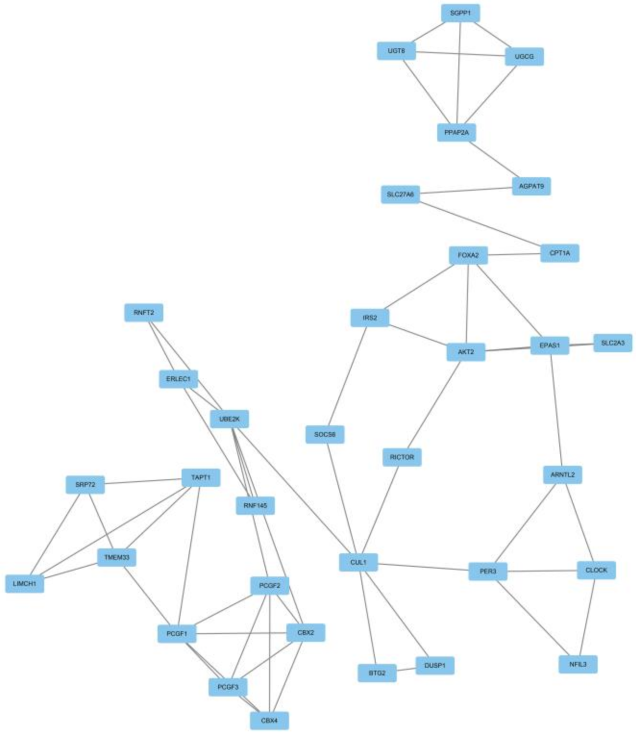

M (2)

GeneSet	P-value	Correct p-value	Nodes
positive regulation of fatty acid beta-oxidation	2.22E-07	1.97E-04	*CPT1A AKT2 IRS2*
positive regulation of fatty acid oxidation	7.58E-07	3.08E-04	*CPT1A AKT2 IRS2*
regulation of fatty acid beta-oxidation	1.04E-06	3.08E-04	*CPT1A AKT2 IRS2*

N (1)

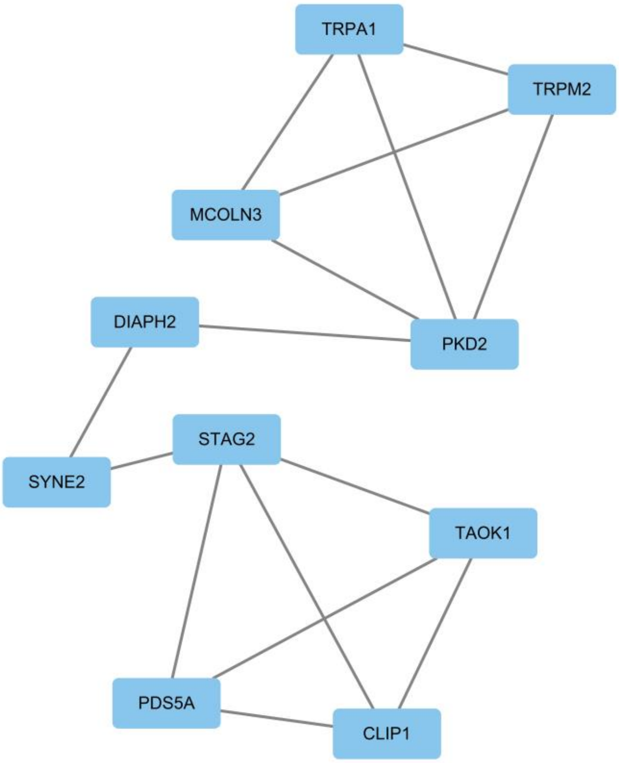

N (2)

GeneSet	P-value	Correct p-value	Nodes
calcium channel activity	7.79E-06	3.04E-03	*TRPM2 TRPA1 PKD2*
detection of mechanical stimulus	2.97E-05	3.04E-03	*TRPA1 PKD2*
organelle organization	3.48E-05	3.04E-03	*TRPM2 CLIP1 DIAPH2 STAG2 PDS5A SYNE2*

O (1)

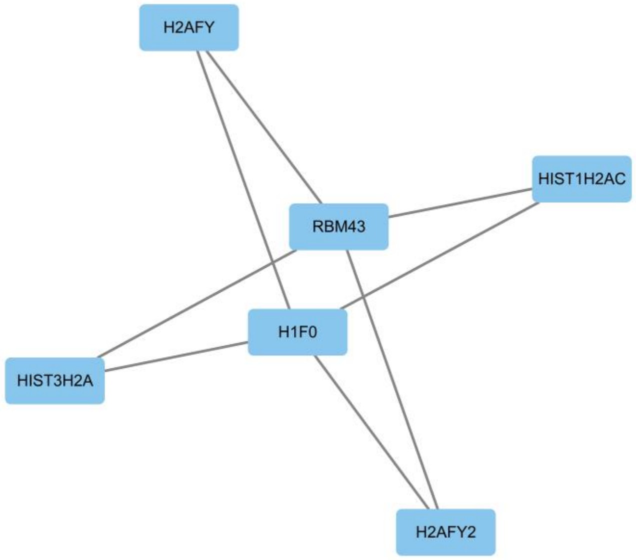

O (2)

GeneSet	P-value	Correct p-value	Nodes
nucleosome	3.33E-12	2.30E-10	*H1F0 HIST3H2A H2AFY H2AFY2 HIST1H2AC*
nucleosome assembly	1.17E-11	2.89E-10	*H1F0 HIST3H2A H2AFY H2AFY2 HIST1H2AC*
chromatin assembly	1.49E-11	2.89E-10	*H1F0 HIST3H2A H2AFY H2AFY2 HIST1H2AC*

Figure S1 Top 15 modules from the protein–protein interaction network. **A (1)** module 1, **A (2)** the enriched pathways of module 1, **B (1)** module 2, **B (2)** the enriched pathways of module 2, **C (1)** module 3, **C (2)** the enriched pathways of module 3, **D (1)** module 4, **D (2)** the enriched pathways of module 4, **E (1)** module 5, **E (2)** the enriched pathways of module 5, **F (1)** module 6, **F (2)** the enriched pathways of module 6, **G (1)** module 7, **G (2)** the enriched pathways of module 7, **H (1)** module 8, **H (2)** the enriched pathways of module 8, **I (1)** module 9, **I (2)** the enriched pathways of module 9, **J (1)** module 10, **J (2)** the enriched pathways of module 10, **K (1)** module 11, **K (2)** the enriched pathways of module 11, **L (1)** module 12, **L (2)** the enriched pathways of module 12, **M (1)** module 13, **M (2)** the enriched pathways of module 13, **N (1)** module 14, **N (2)** the enriched pathways of module 14, **O (1)** module15, **O (2)** the enriched pathways of module 15.

A

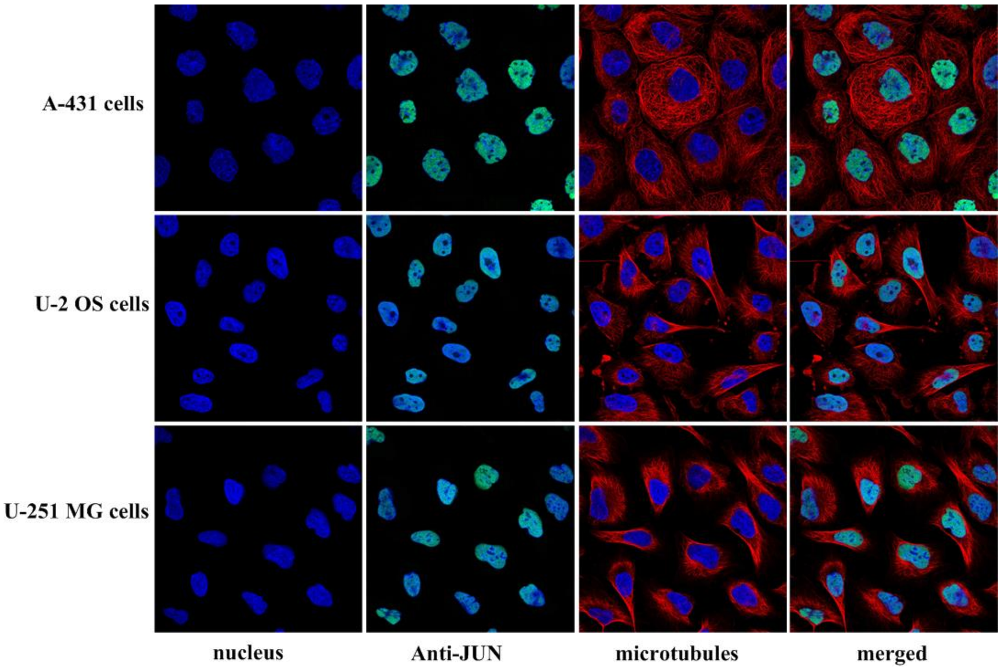

B

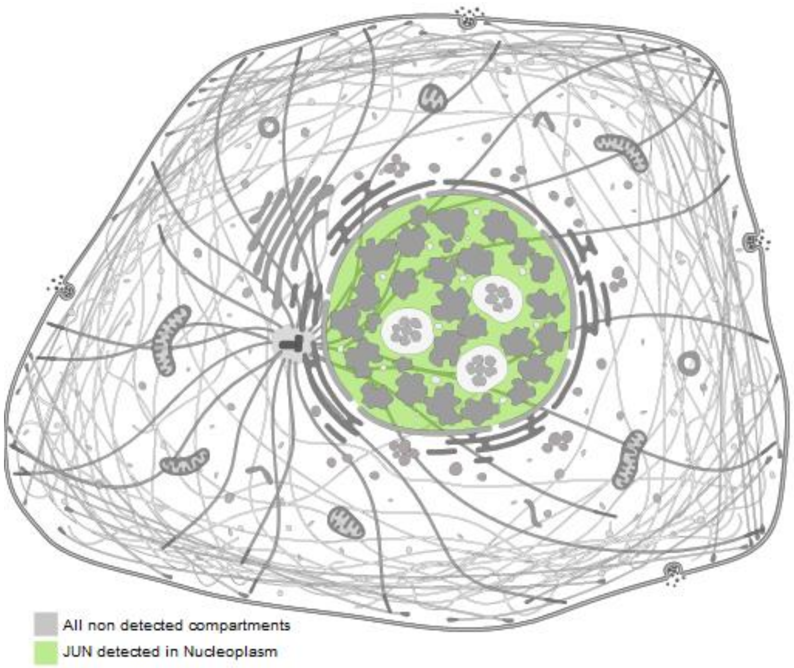

Figure S2 *JUN* localization in human cells[1] (https://www.proteinatlas.org/ENSG00000177606-JUN/cell). **A** The localization of *JUN* protein in human cells. Blue: nucleus; Green: *JUN*; Red: microtubules. **B** Proposed schematic outline of *JUN* in human cells (The Human Protein Atlas images are licensed under CC BY-SA 3.0 (https://creativecommons.org/licenses/by-sa/3.0), (https://creativecommons.org/licenses/by-sa/3.0/legalcode)).

A

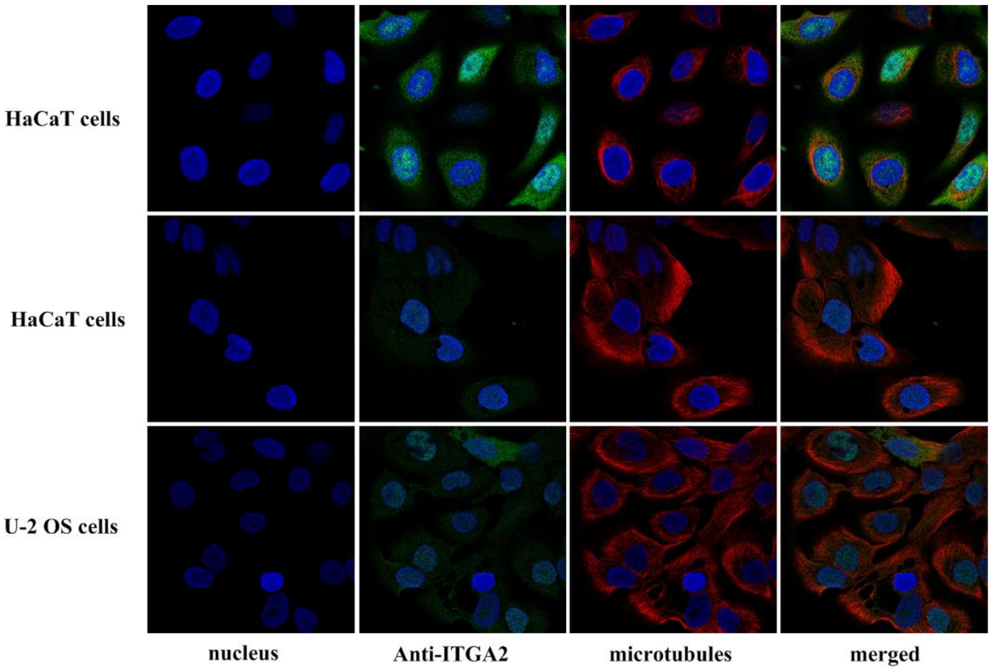

B

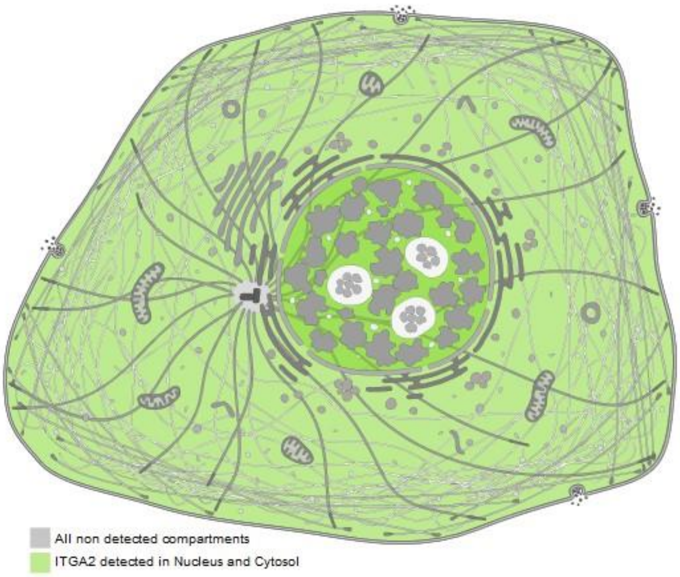

(B)

Figure S3 *ITGA2* localization in human cells[1] (https://www.proteinatlas.org/ENSG00000164171-ITGA2/cell). **A** The localization of *ITGA2* protein in human cells. Blue: nucleus; Green: *ITGA2*; Red: microtubules. **B** Proposed schematic outline of *ITGA2* in human cells (The Human Protein Atlas images are licensed under CC BY-SA 3.0 (https://creativecommons.org/licenses/by-sa/3.0), (https://creativecommons.org/licenses/by-sa/3.0/legalcode)).

A

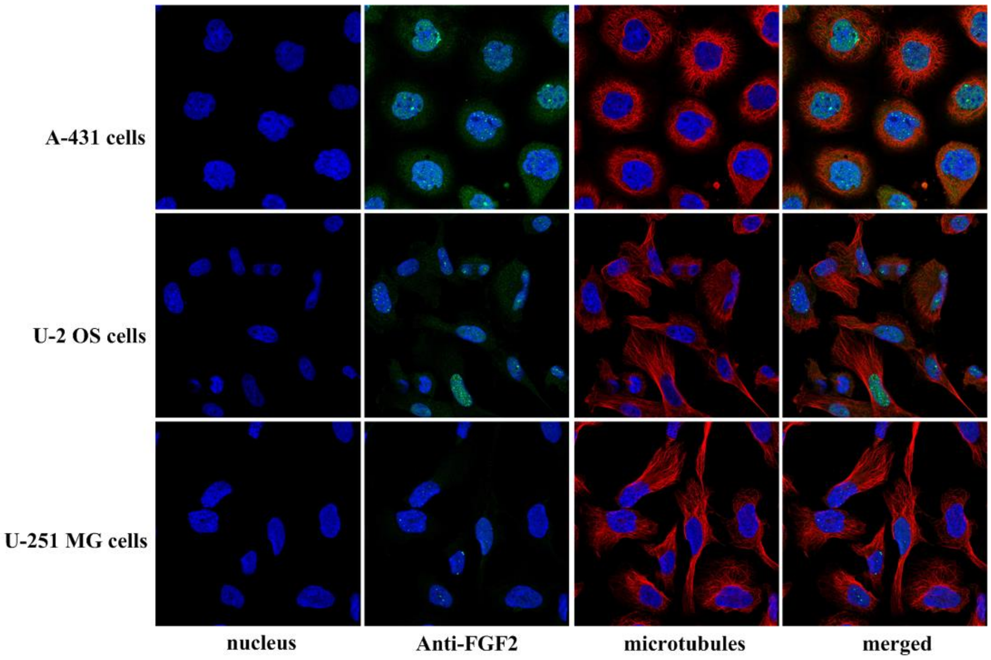

B

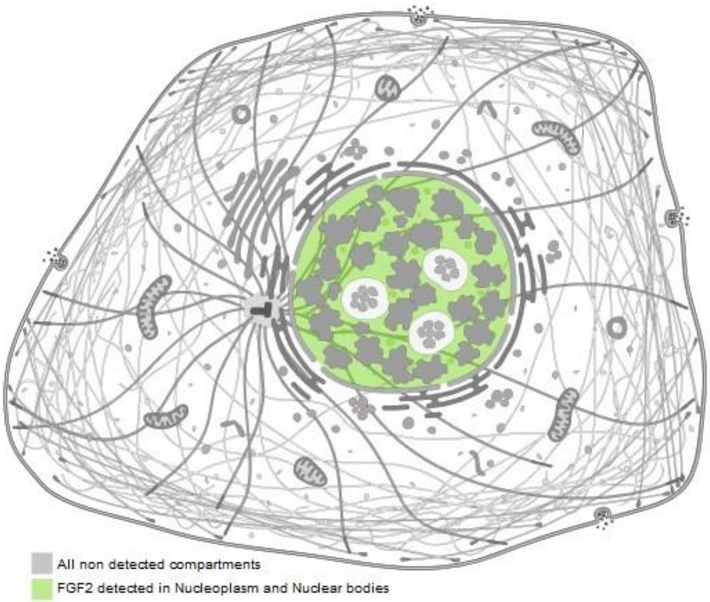

Figure S4 *FGF2* localization in human cells[1] (https://www.proteinatlas.org/ENSG00000138685-FGF2/cell). **A** The localization of *FGF2* protein in human cells. Blue: nucleus; Green: *FGF2*; Red: microtubules. **B** Proposed schematic outline of *FGF2* in human cells (The Human Protein Atlas images are licensed under CC BY-SA 3.0 (https://creativecommons.org/licenses/by-sa/3.0), (https://creativecommons.org/licenses/by-sa/3.0/legalcode)).

A

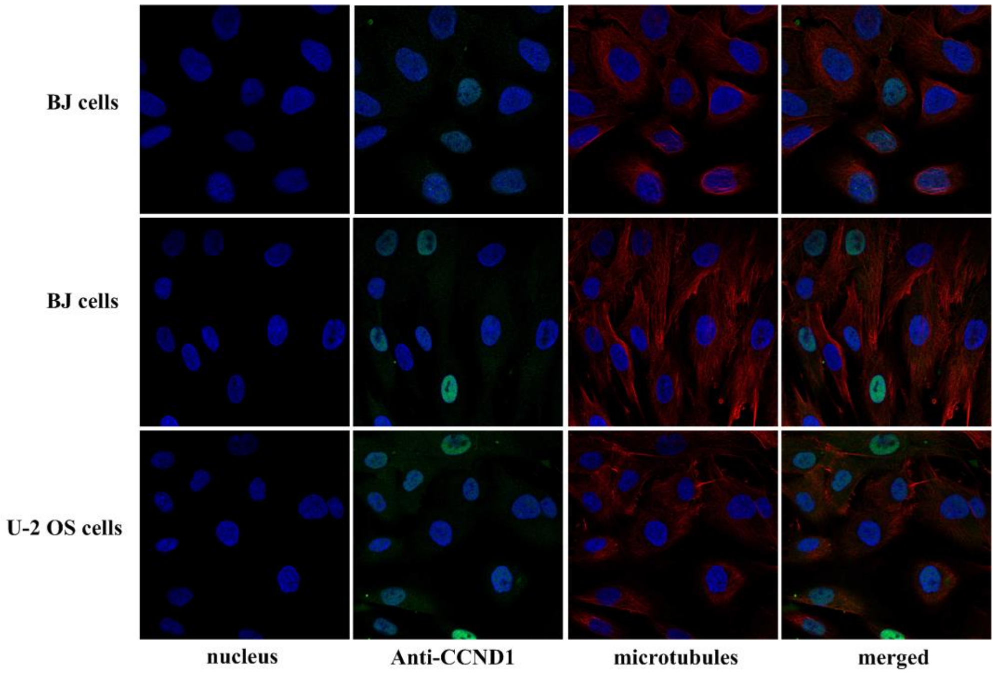

B

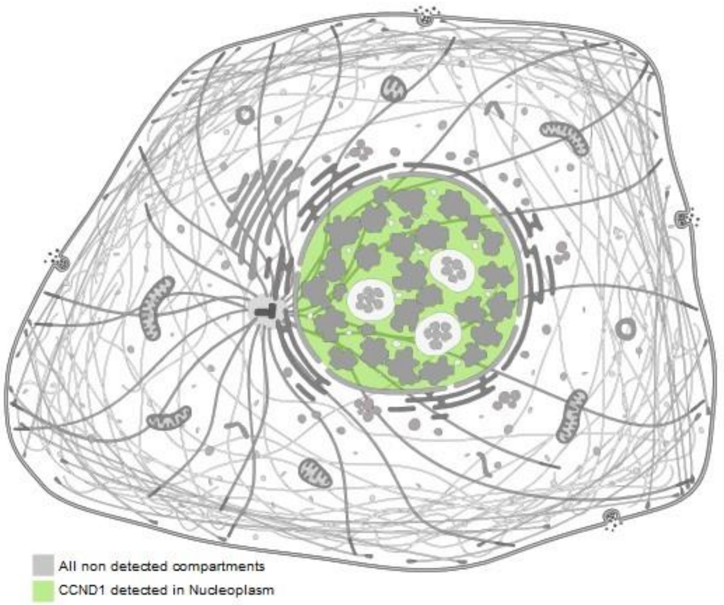

Figure S5 *CCND1* localization in human cells[1] (https://www.proteinatlas.org/ENSG00000110092-CCND1/cell). **A** The localization of *CCND1* protein in human cells. Blue: nucleus; Green: *CCND1*; Red: microtubules. **B** Proposed schematic outline of *CCND1* in human cells (The Human Protein Atlas images are licensed under CC BY-SA 3.0 (https://creativecommons.org/licenses/by-sa/3.0), (https://creativecommons.org/licenses/by-sa/3.0/legalcode)).

A

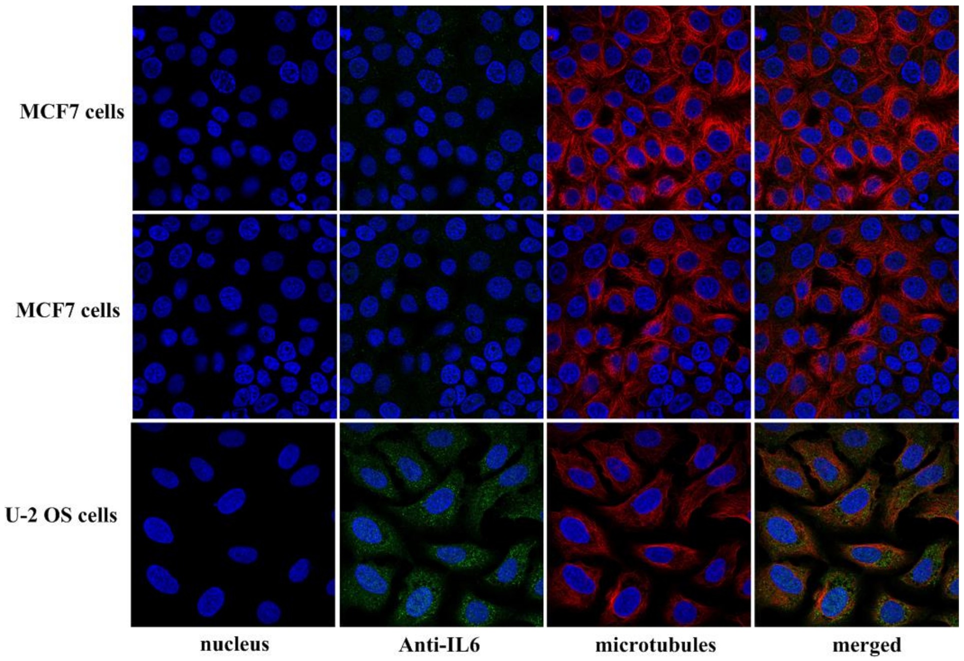

B

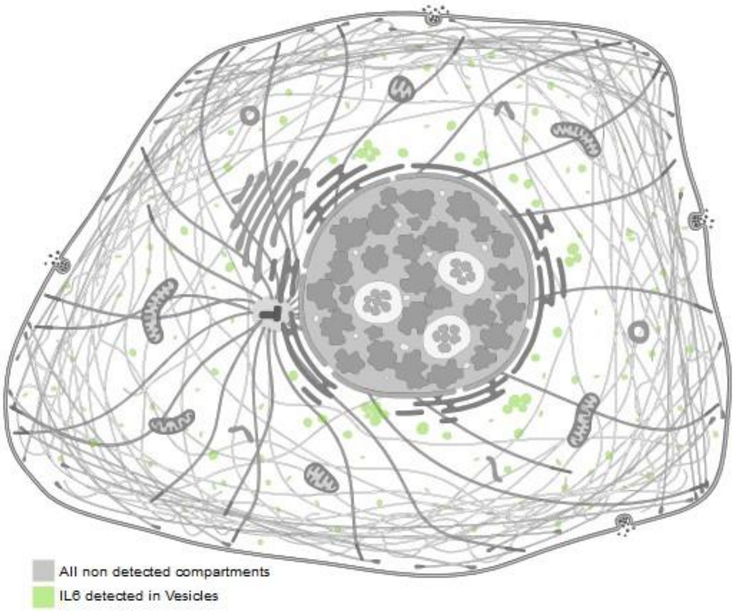

Figure S6 *IL6* localization in human cells[1] (https://www.proteinatlas.org/ENSG00000136244-IL6/cell). **A** The localization of *IL6* protein in human cells. Blue: nucleus; Green: *IL6*; Red: microtubules. **B** Proposed schematic outline of *IL6* in human cells (The Human Protein Atlas images are licensed under CC BY-SA 3.0 (https://creativecommons.org/licenses/by-sa/3.0), (https://creativecommons.org/licenses/by-sa/3.0/legalcode)).

A

B

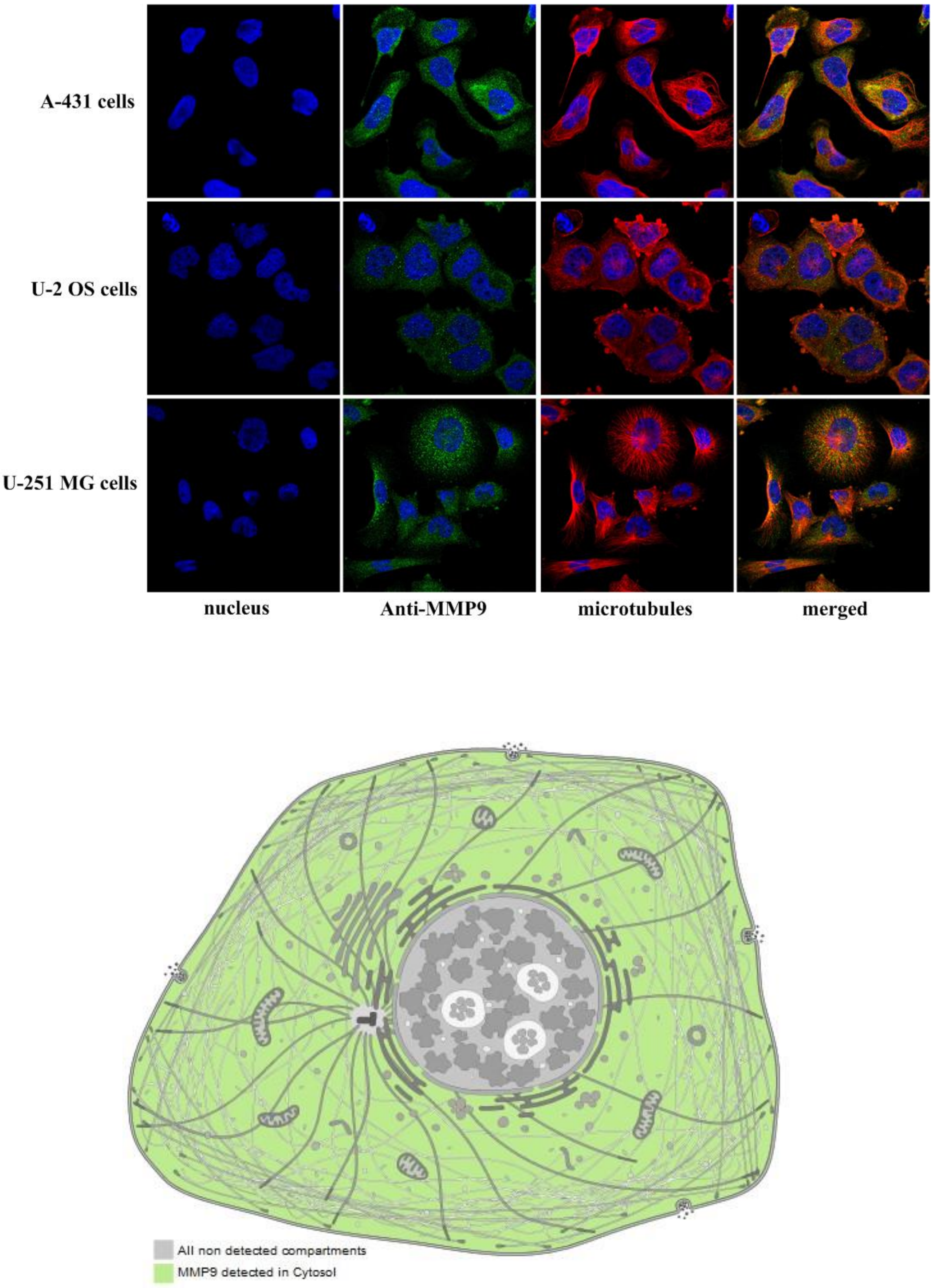

Figure 7 *MMP9* localization in human cells[1] (https://www.proteinatlas.org/ENSG00000100985-MMP9/cell). **A** The localization of *MMP9* protein in human cells. Blue: nucleus; Green: *MMP9*; Red: microtubules. **B** Proposed schematic outline of *MMP9* in human cells (The Human Protein Atlas images are licensed under CC BY-SA 3.0 (https://creativecommons.org/licenses/by-sa/3.0), (https://creativecommons.org/licenses/by-sa/3.0/legalcode)).

A

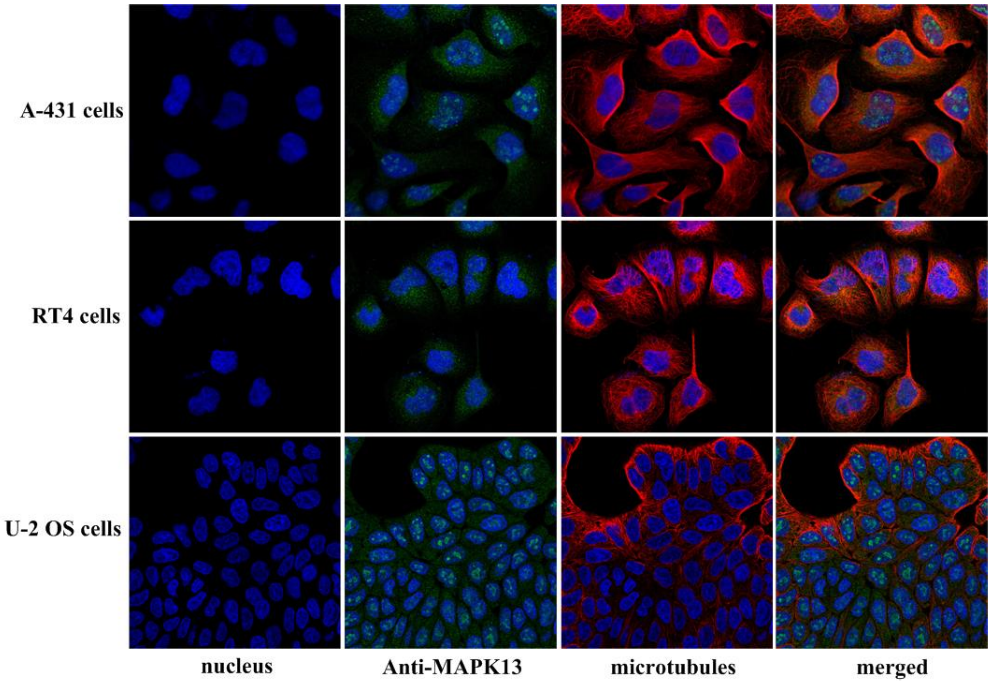

B

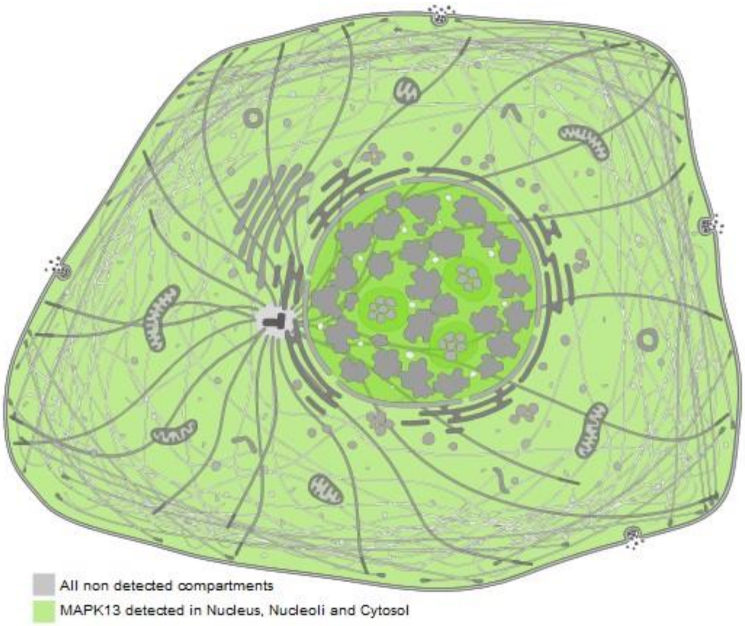

Figure S8 *MAPK13* localization in human cells[1] (https://www.proteinatlas.org/ENSG00000156711-MAPK13/cell). **A** The localization of *MAPK13* protein in human cells. Blue: nucleus; Green: *MAPK13*; Red: microtubules. **B** Proposed schematic outline of *MAPK13* in human cells (The Human Protein Atlas images are licensed under CC BY-SA 3.0 (https://creativecommons.org/licenses/by-sa/3.0), (https://creativecommons.org/licenses/by-sa/3.0/legalcode)).

A

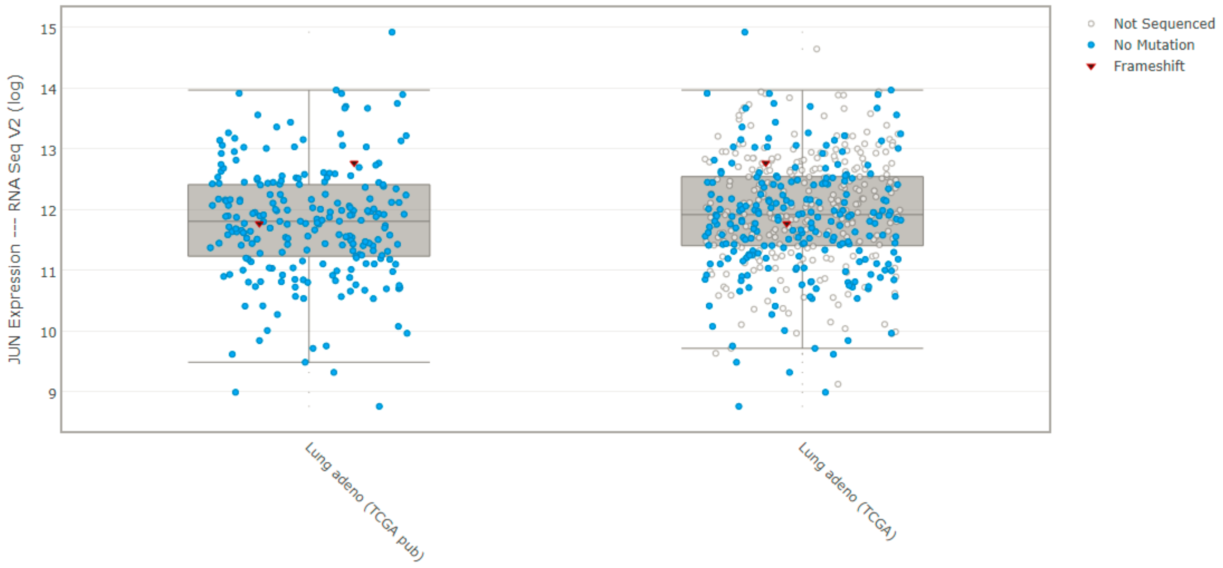

B

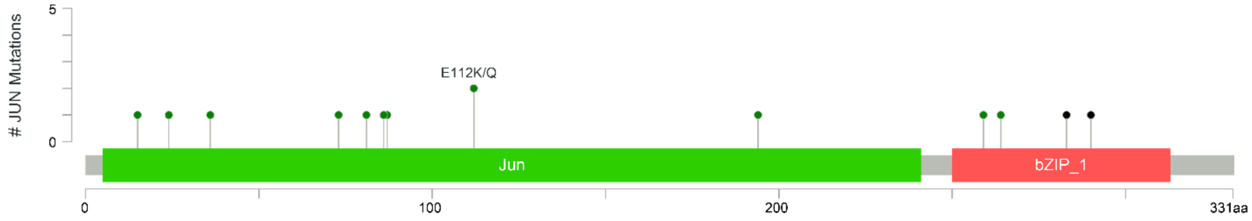

Figure S9 Genetic alteration of *JUN* was analyzed using the cBioPortal. **A** Relative expression level as a function of relative copy number of *JUN* was plotted in two lung adenocarcinoma databases respectively. (Lung Adenocarcinoma (TCGA, Nature 2014); Lung Adenocarcinoma (TCGA, Provisional). White circle: Not sequenced; Blue circle: No mutation; Red triangle: Frameshift.) **B** The distribution of *JUN* mutations in non–small-cell lung cancer across protein domains.

A

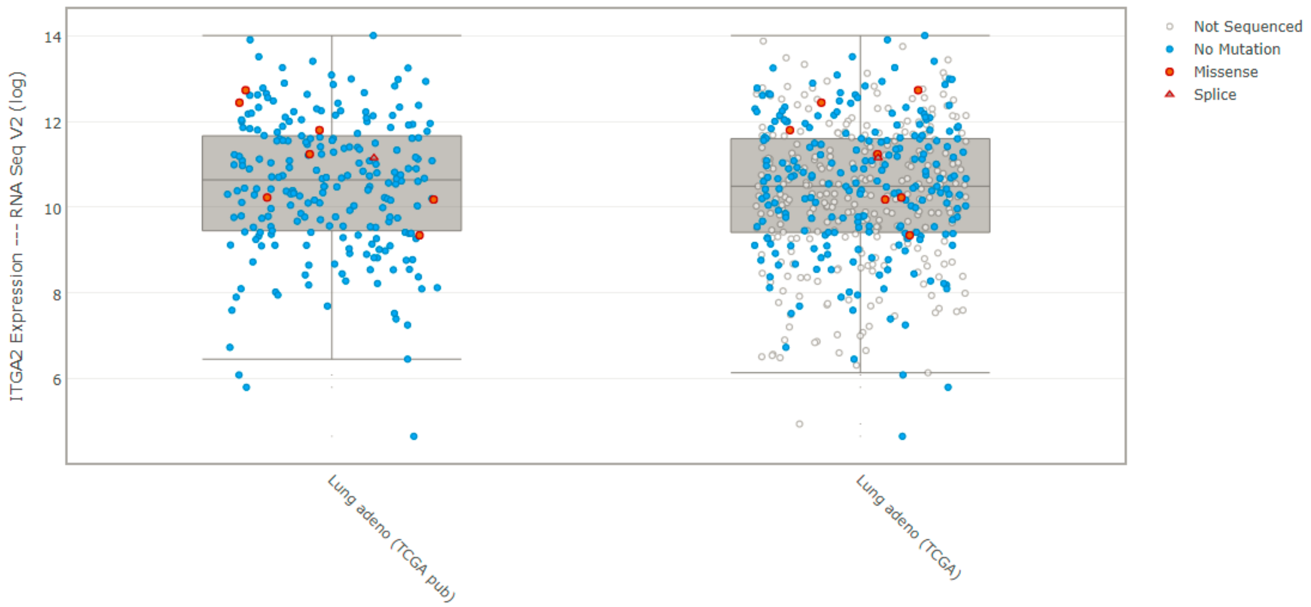

B

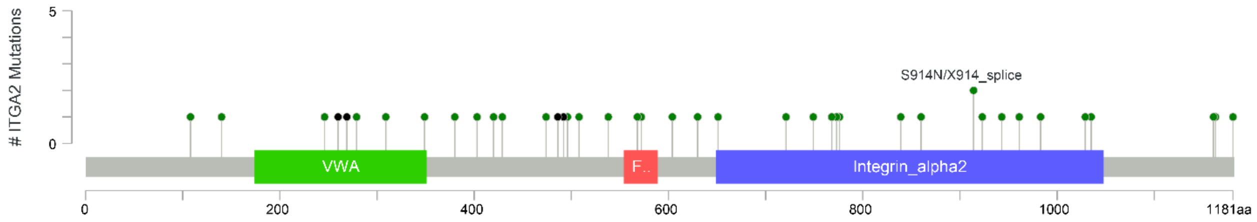

Figure S10 Genetic alteration of *ITGA2* was analyzed using the cBioPortal. **A** Relative expression level as a function of relative copy number of *ITGA2* gene was plotted in two lung adenocarcinoma databases respectively. (Lung Adenocarcinoma (TCGA, Nature 2014); Lung Adenocarcinoma (TCGA, Provisional). White circle: Not sequenced; Blue circle: No mutation; Red circle: Missense; Red triangle: Splice.). **B** The distribution of *ITGA2* mutations in non–small-cell lung cancer across protein domains.

A

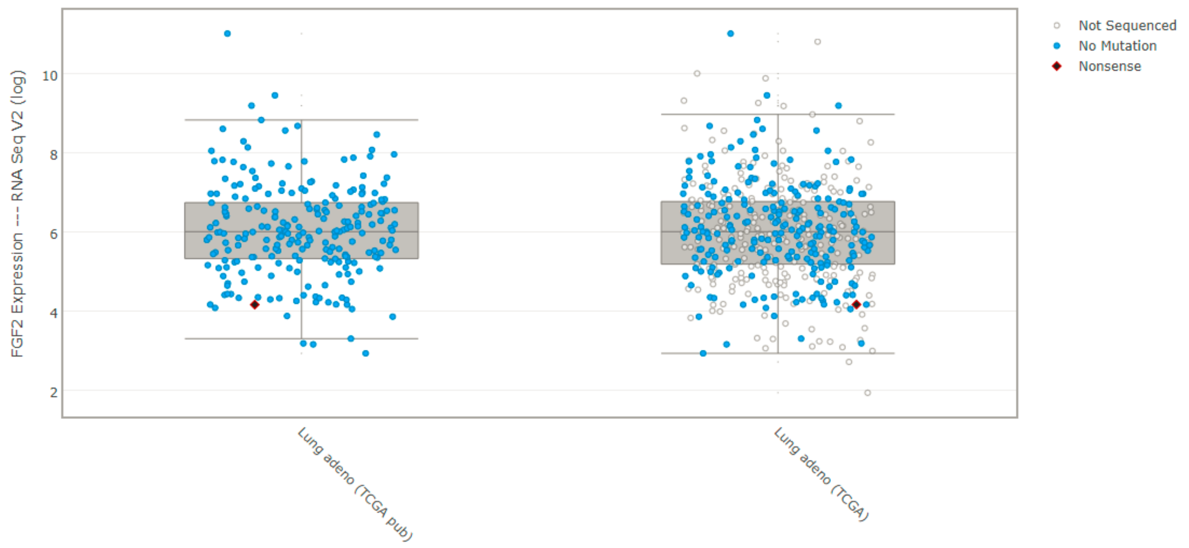

B

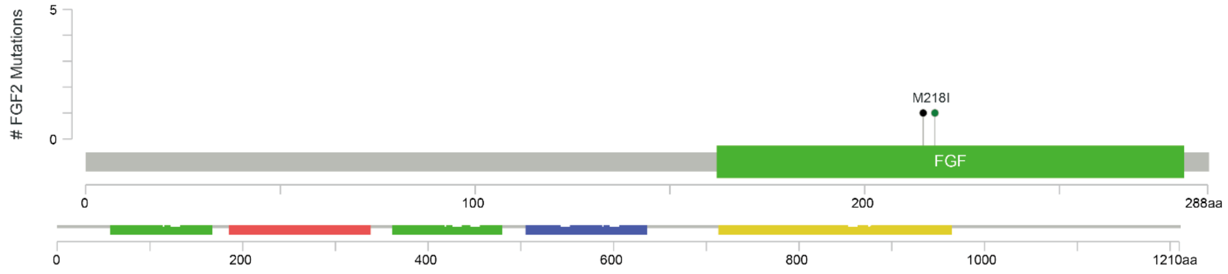

Figure S11 Genetic alteration of *FGF2* was analyzed using the cBioPortal. **A** Relative expression level as a function of relative copy number of *FGF2* was plotted in two lung adenocarcinoma databases respectively. (Lung Adenocarcinoma (TCGA, Nature 2014); Lung Adenocarcinoma (TCGA, Provisional). White circle: Not sequenced; Blue circle: No mutation; Red lozenge: Nonsense.). **B** The distribution of *ITGA2* mutations in non–small-cell lung cancer across protein domains.

A

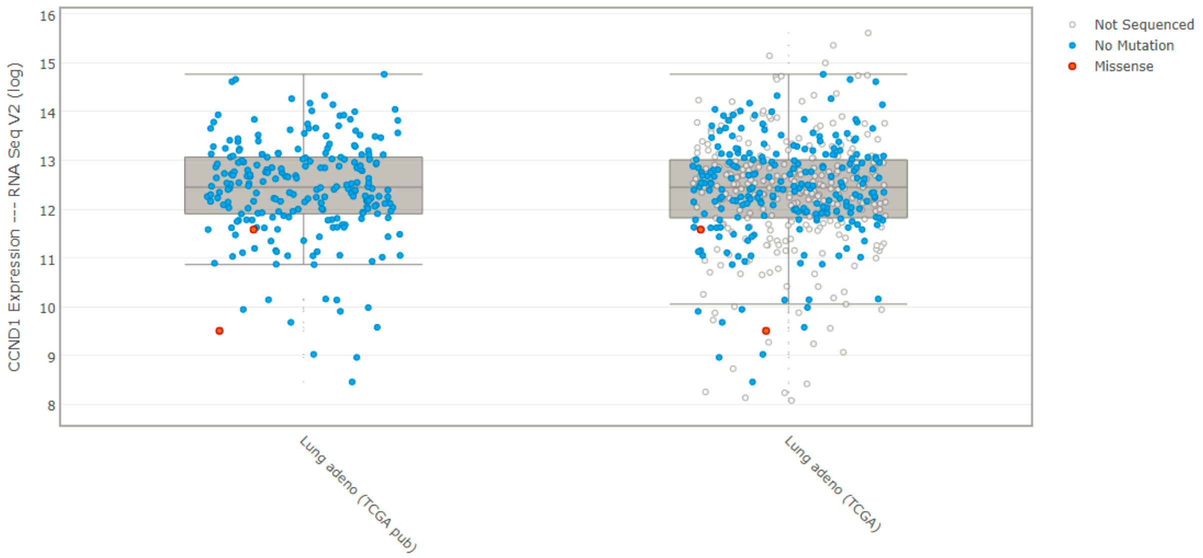

B

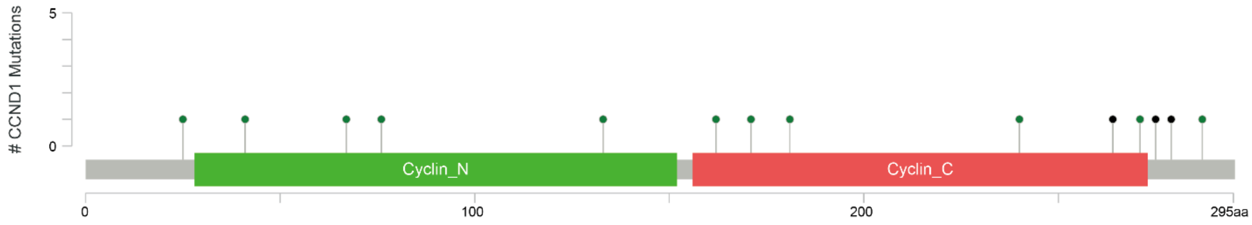

Figure S12 Genetic alteration of *CCND1* was analyzed using the cBioPortal. **A** Relative expression level as a function of relative copy number of *CCND1* gene was plotted in two lung adenocarcinoma databases respectively. (Lung Adenocarcinoma (TCGA, Nature 2014); Lung Adenocarcinoma (TCGA, Provisional). White circle: Not sequenced; Blue circle: No mutation; Red circle: Missense.). **B** The distribution of *CCND1* mutations in non–small-cell lung cancer across protein domains.

A

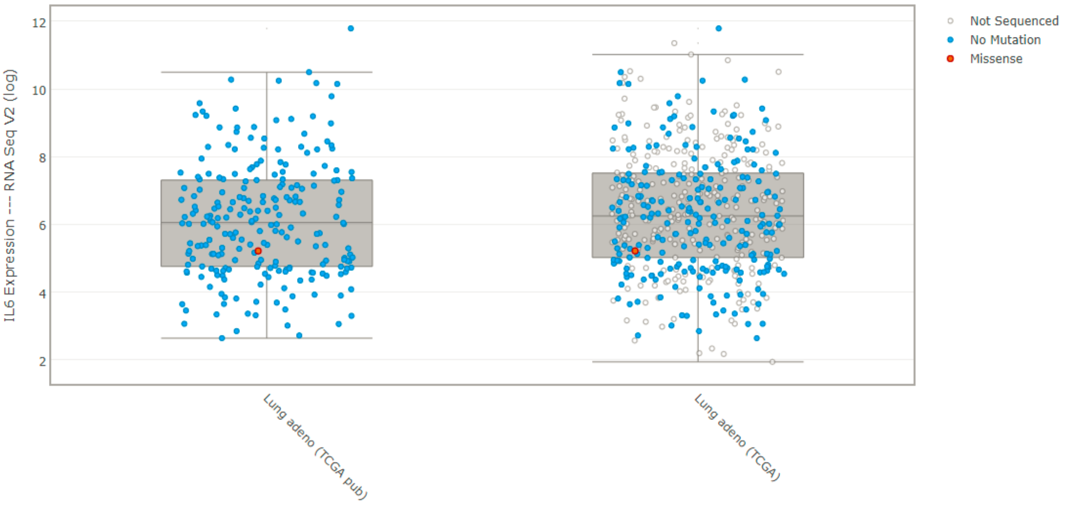

B

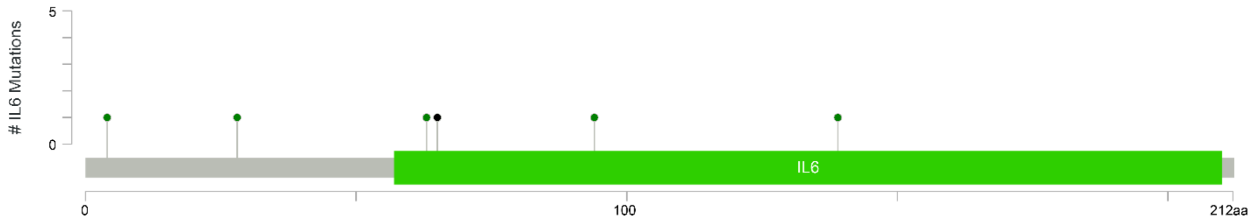

Figure S13 Genetic alteration of *IL6* was analyzed using the cBioPortal. **A** Relative expression level as a function of relative copy number of *IL6* gene was plotted in two lung adenocarcinoma databases respectively. (Lung Adenocarcinoma (TCGA, Nature 2014); Lung Adenocarcinoma (TCGA, Provisional). White circle: Not sequenced; Blue circle: No mutation; Red circle: Missense.). **B** The distribution of *IL6* mutations in non–small-cell lung cancer across protein domains.

A

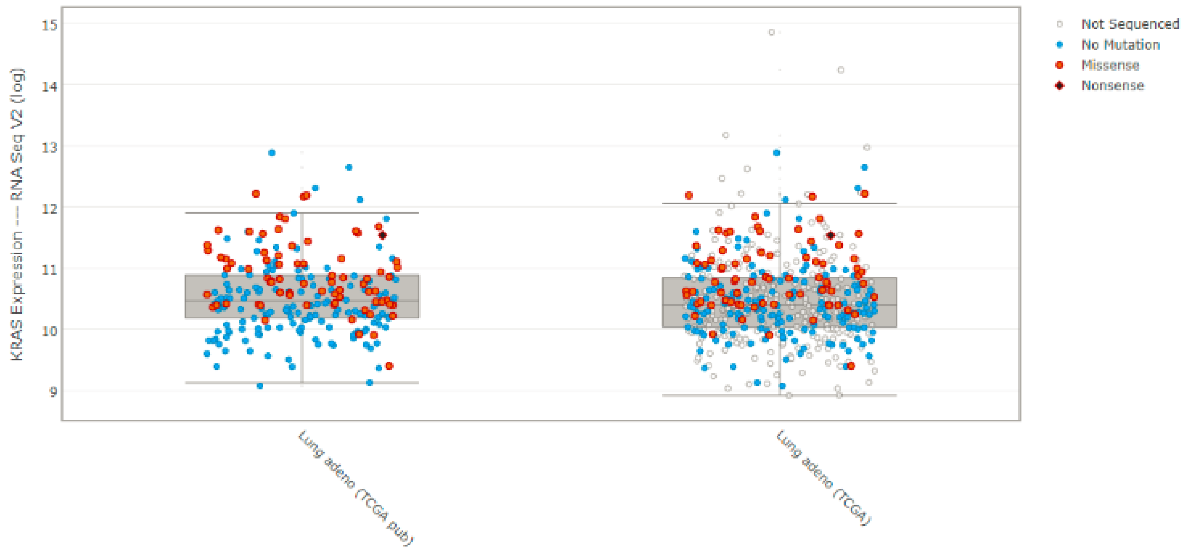

B

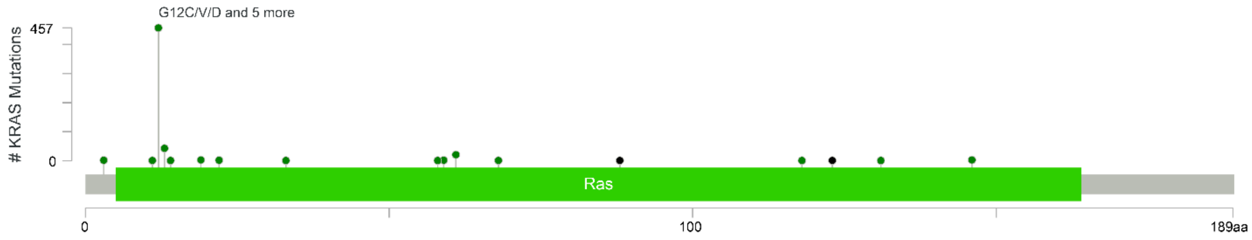

Figure S14 Genetic alteration of *KRAS* was analyzed using the cBioPortal. **A** Relative expression level as a function of relative copy number of *KRAS* gene was plotted in two lung adenocarcinoma databases respectively. (Lung Adenocarcinoma (TCGA, Nature 2014); Lung Adenocarcinoma (TCGA, Provisional). Red circle: Missense; White circle: Not sequenced; Blue circle: No mutation; Red lozenge: Nonsense.). **B** The distribution of *KRAS* mutations in non–small-cell lung cancer across protein domains.

A

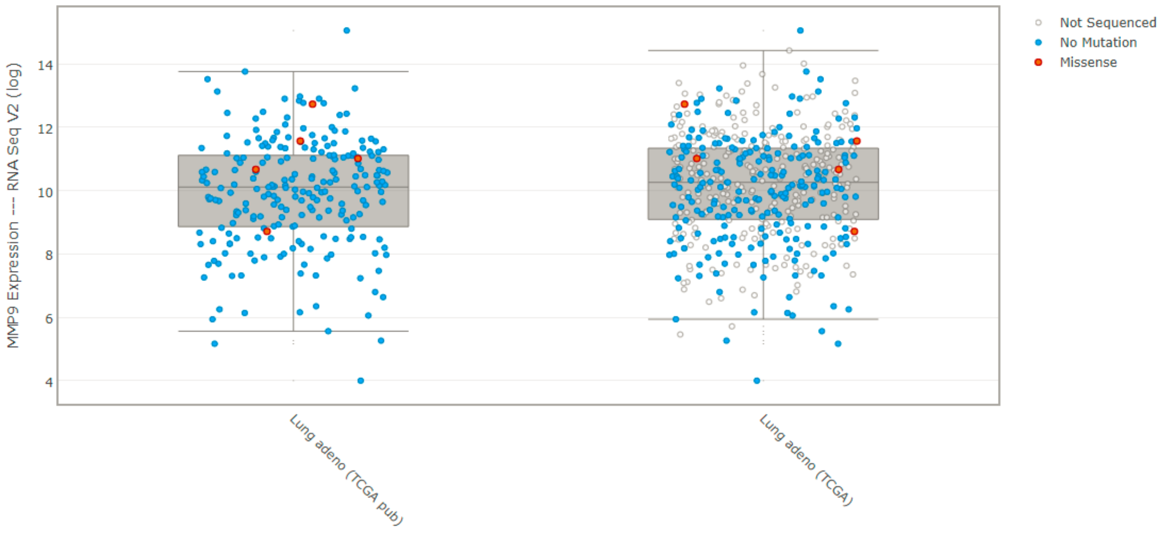

B

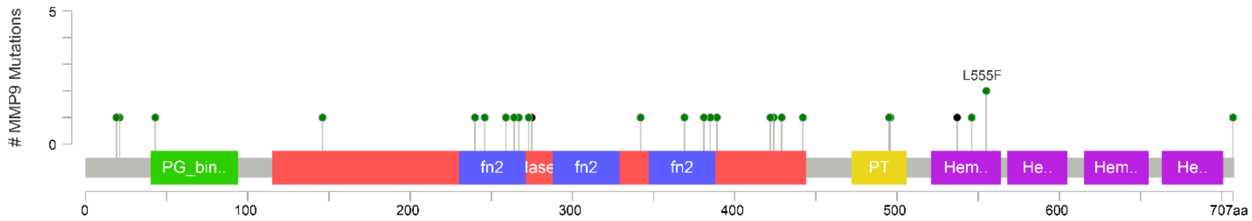

Figure S15 Genetic alteration of *MMP9* was analyzed using the cBioPortal. **A** Relative expression level as a function of relative copy number of *MMP9* gene was plotted in two lung adenocarcinoma databases respectively. (Lung Adenocarcinoma (TCGA, Nature 2014); Lung Adenocarcinoma (TCGA, Provisional). White circle: Not sequenced; Blue circle: No mutation; Red circle: Missense.). **B** The distribution of *MMP9* mutations in non–small-cell lung cancer across protein domains.

A

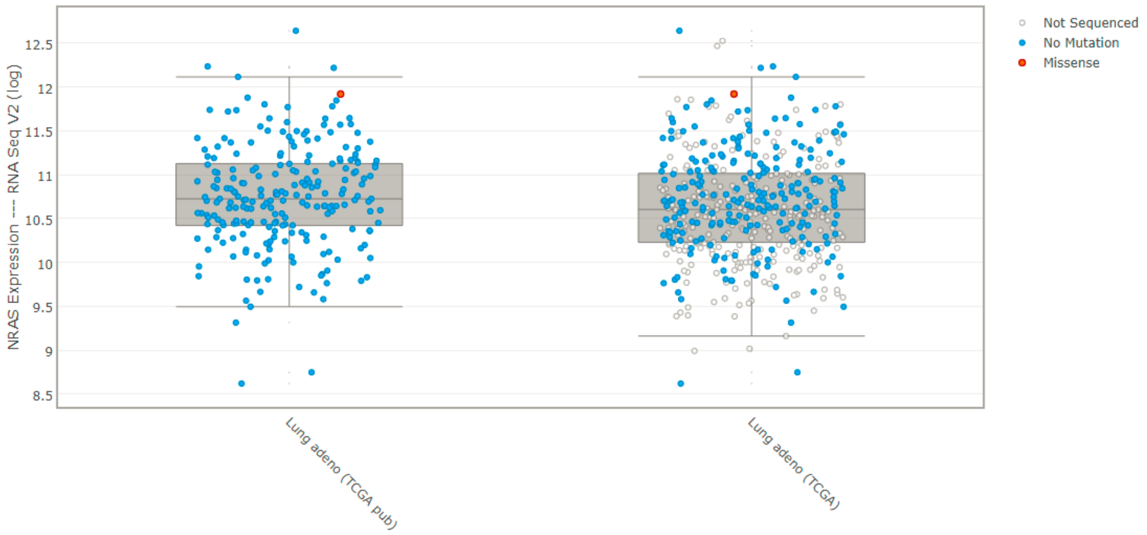

B

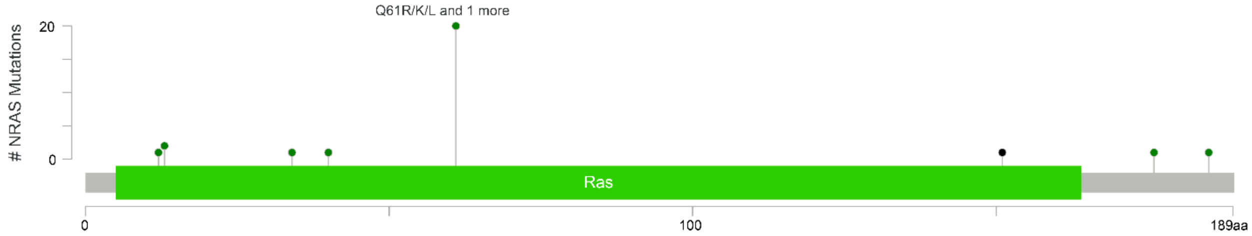

Figure S16 Genetic alteration of *NRAS* was analyzed using the cBioPortal. **A** Relative expression level as a function of relative copy number of *NRAS* gene was plotted in two lung adenocarcinoma databases respectively. (Lung Adenocarcinoma (TCGA, Nature 2014); Lung Adenocarcinoma (TCGA, Provisional). White circle: Not sequenced; Blue circle: No mutation; Red circle: Missense.) **B** The distribution of *NRAS* mutations in non–small-cell lung cancer across protein domains.

A

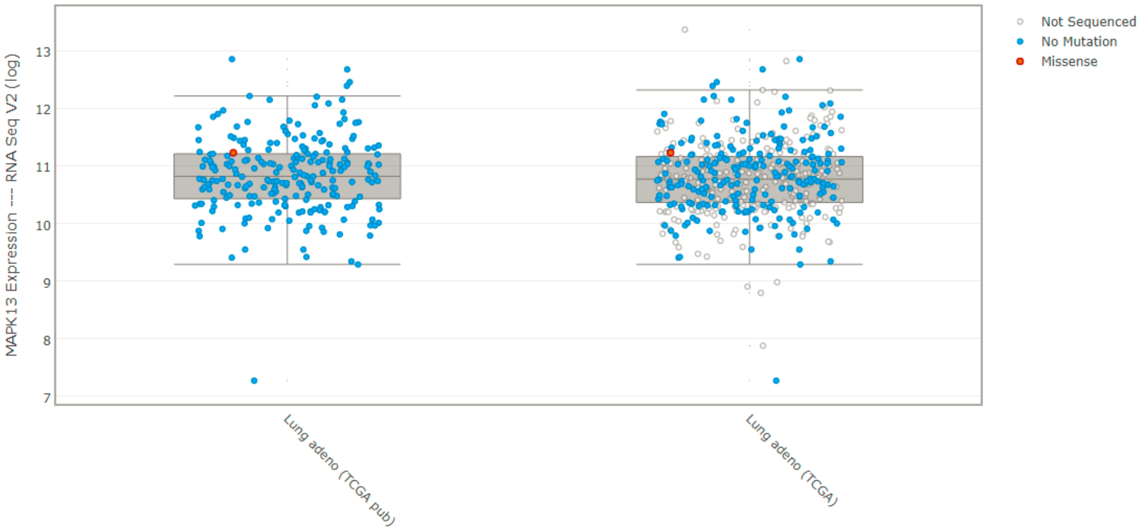

B

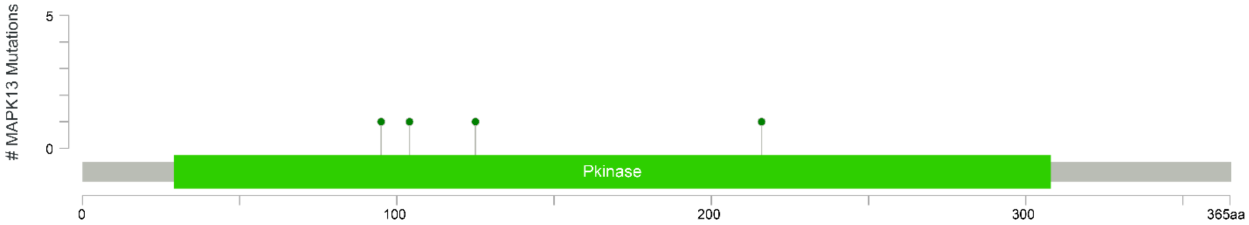

Figure S17 Genetic alteration of *MAPK13* was analyzed using the cBioPortal. **A** Relative expression level as a function of relative copy number of *MAPK13* gene was plotted in two lung adenocarcinoma databases respectively. (Lung Adenocarcinoma (TCGA, Nature 2014); Lung Adenocarcinoma (TCGA, Provisional). White circle: Not sequenced; Blue circle: No mutation; Red circle: Missense.). **B** The distribution of *MAPK13* mutations in non–small-cell lung cancer across protein domains.

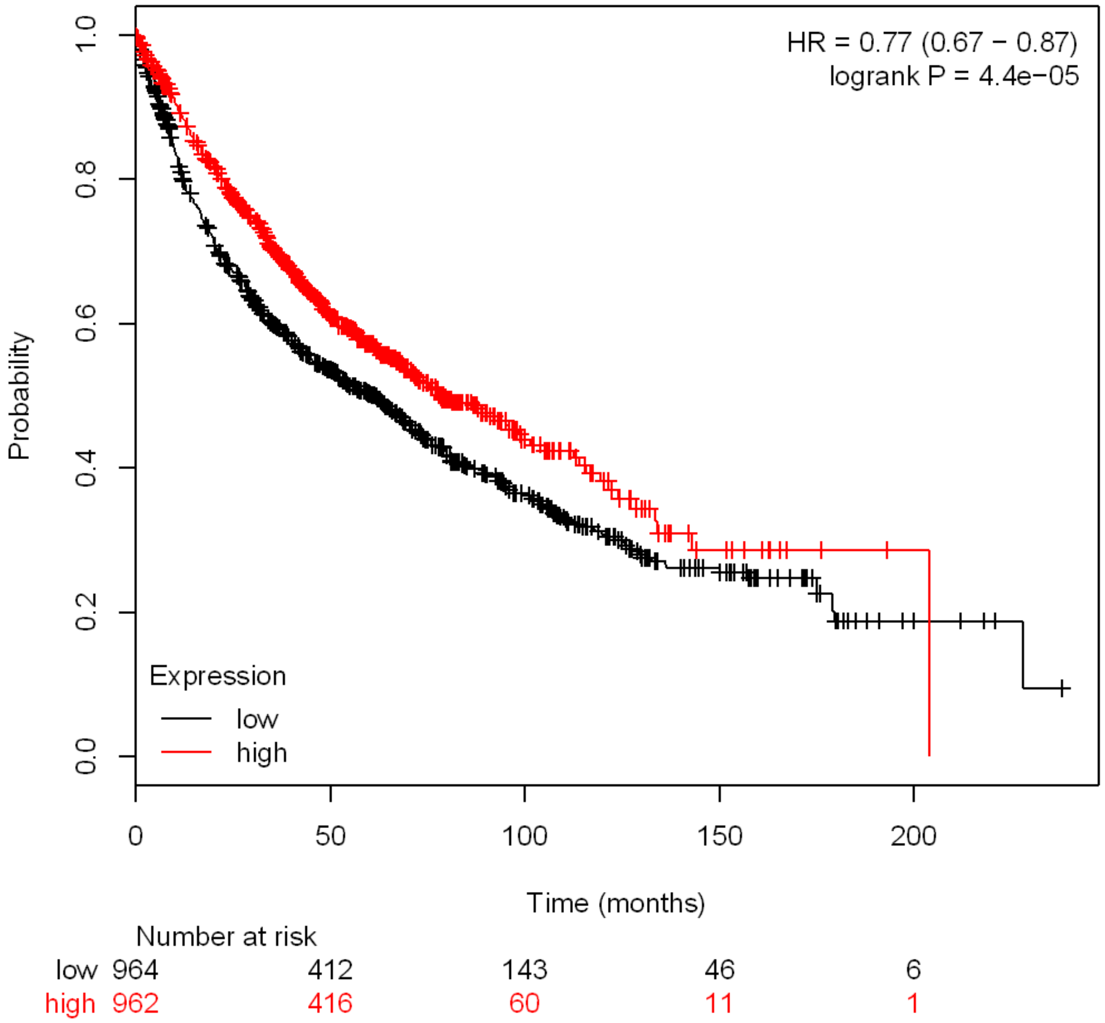

Figure S18 Kaplan–Meier plot for *JUN* associated with patient survival.

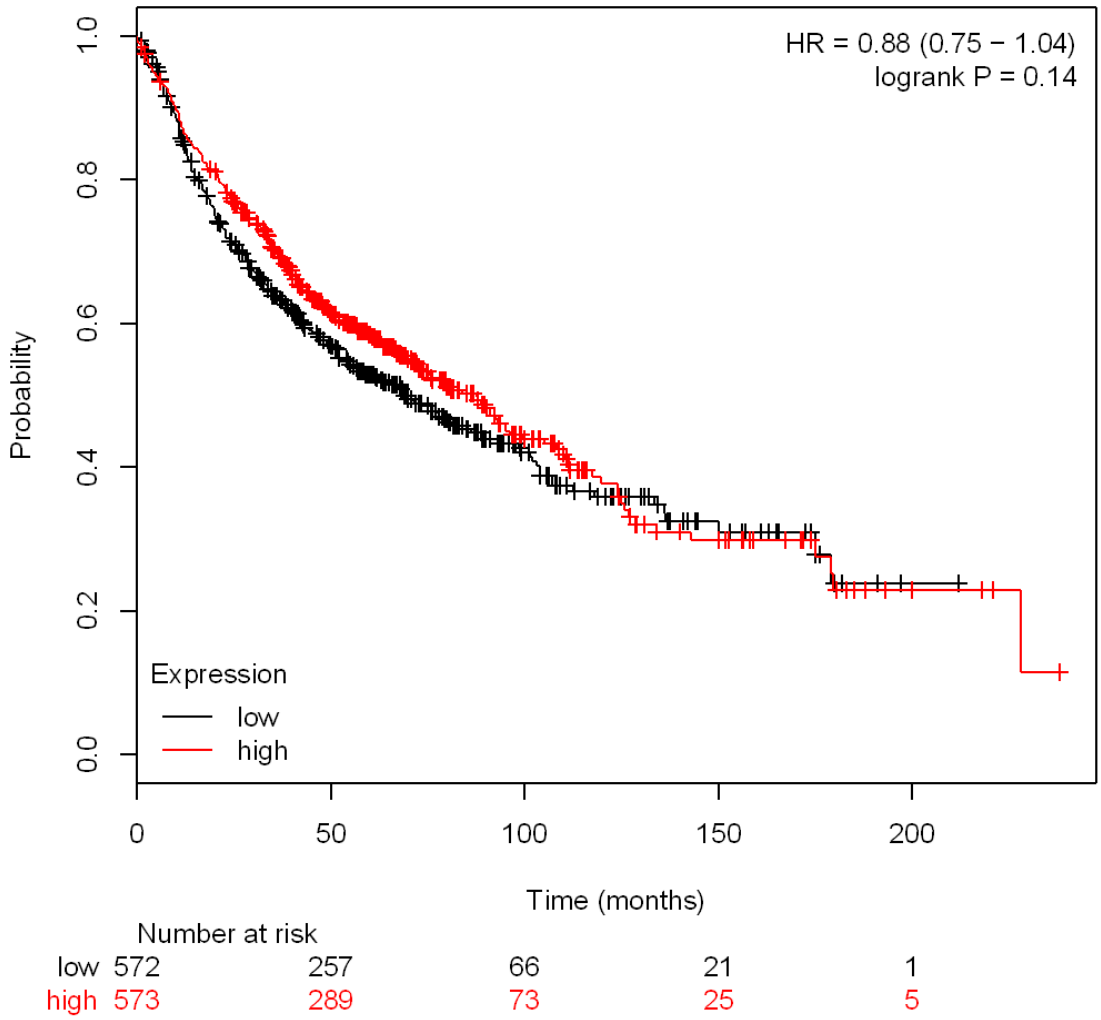

Figure S19 Kaplan–Meier plot for *ITGA2* associated with patient survival.

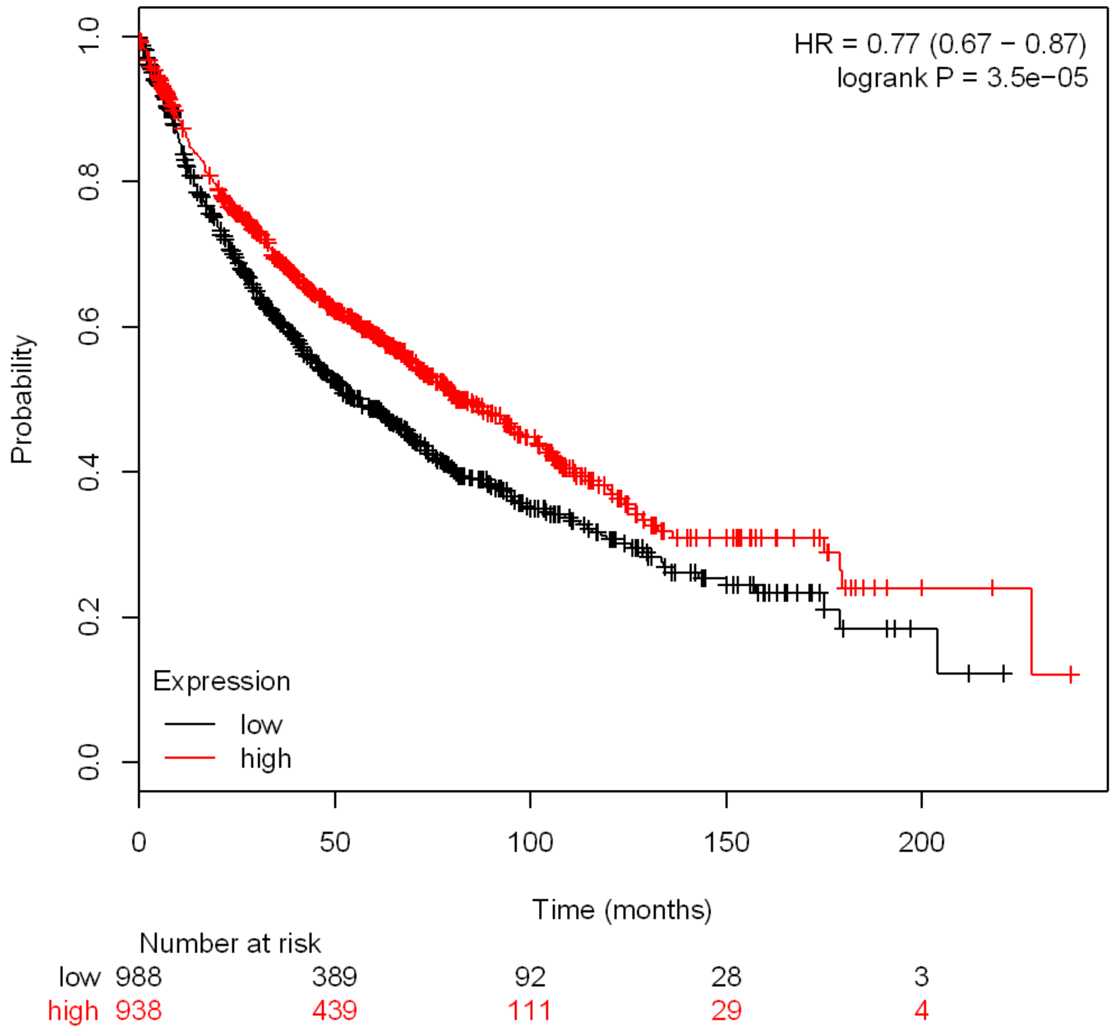

Figure S20 Kaplan–Meier plot for *FGF2* associated with patient survival.

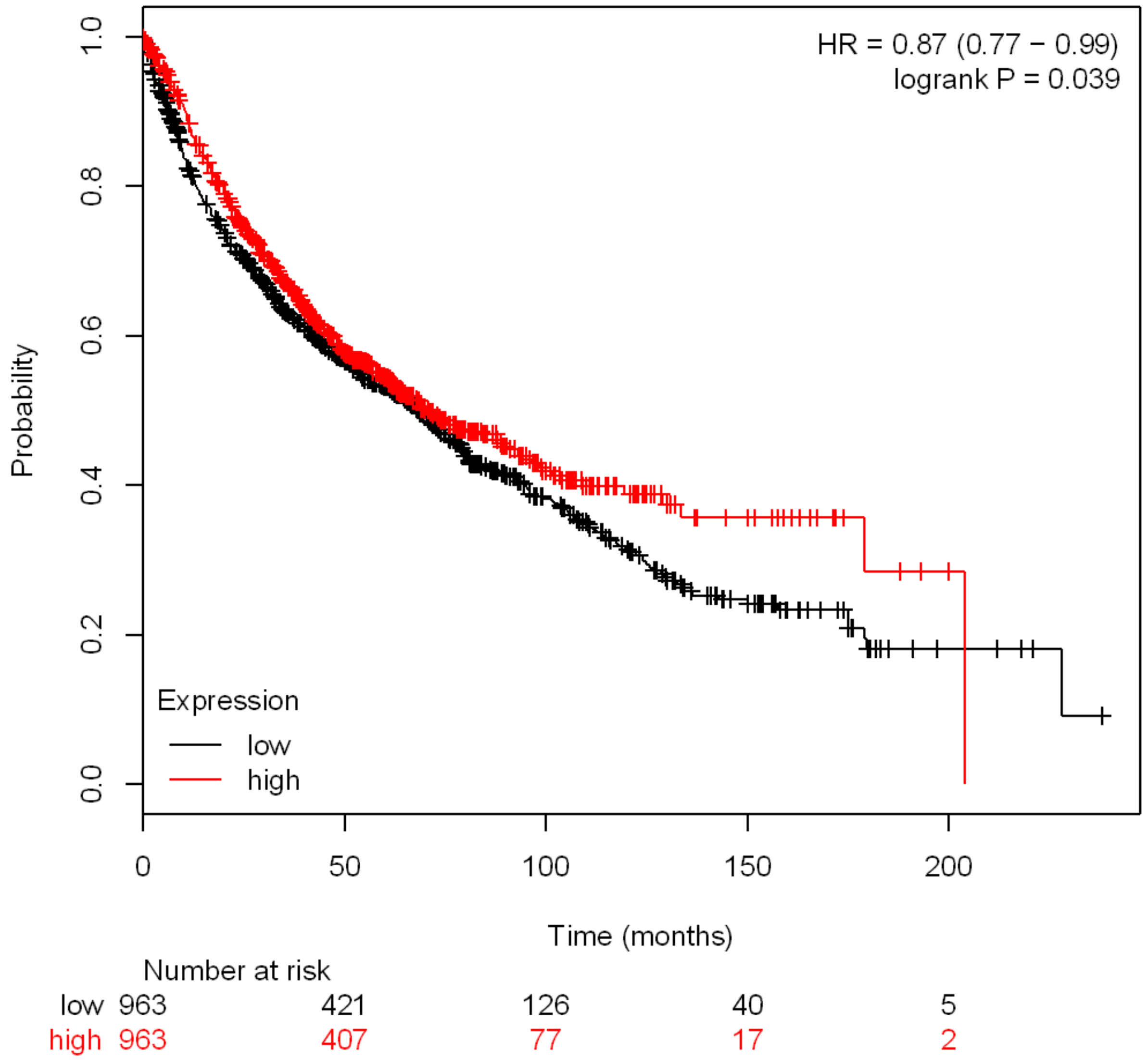

Figure S21 Kaplan–Meier plot for *CCND1* associated with patient survival.

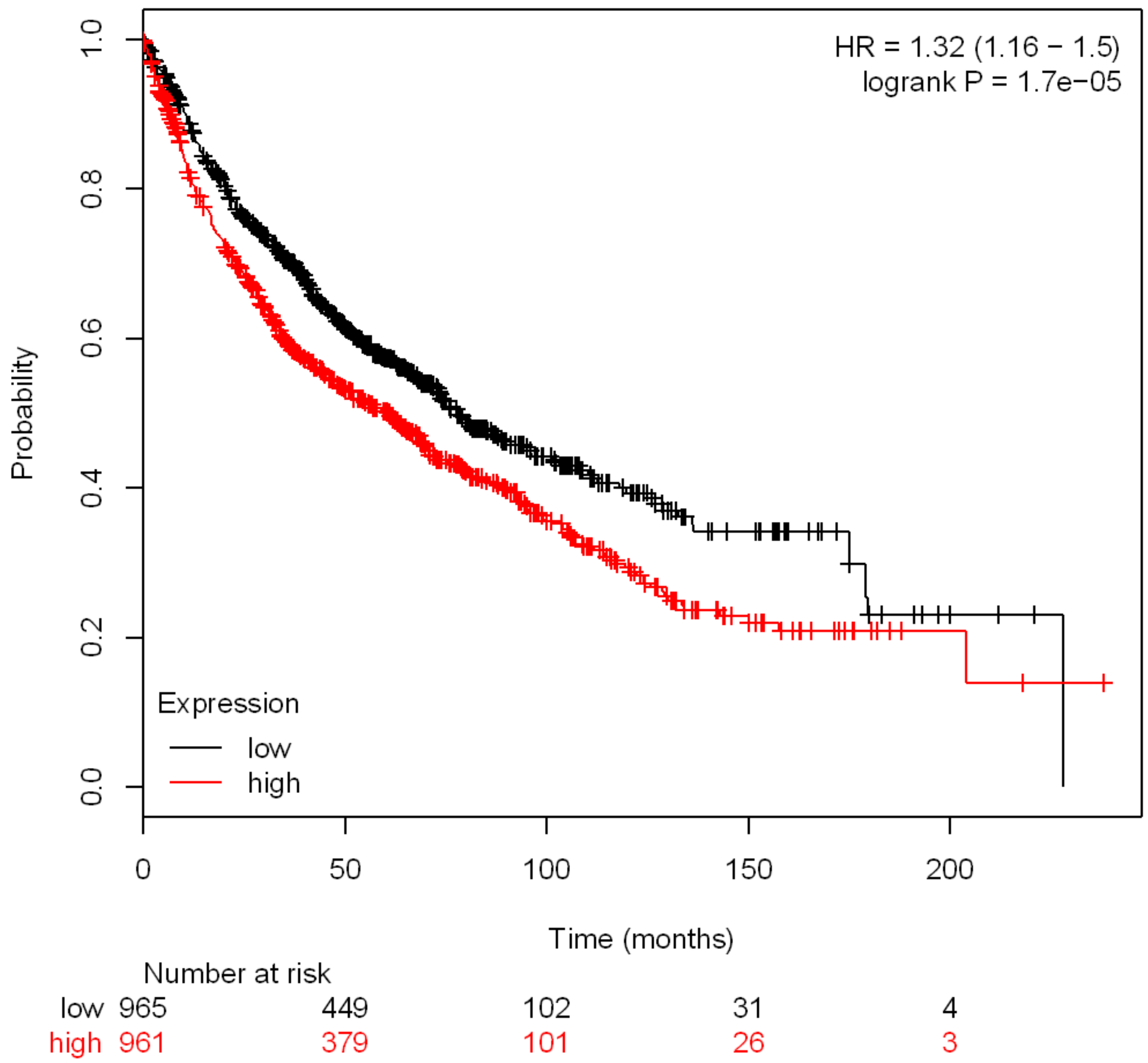

Figure S22 Kaplan–Meier plot for *IL6* associated with patient survival.

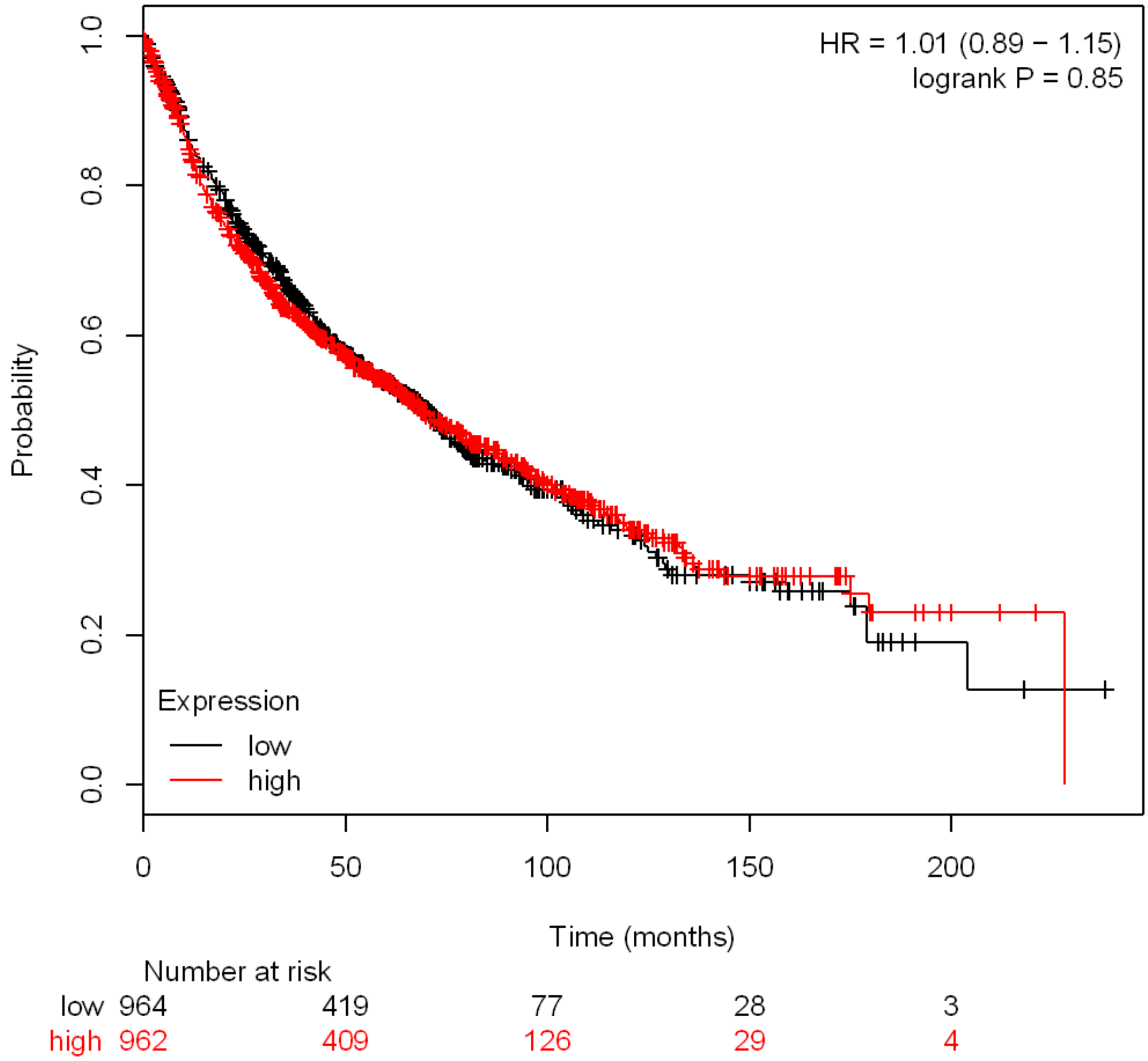

Figure S23 Kaplan–Meier plot for *KRAS* associated with patient survival.

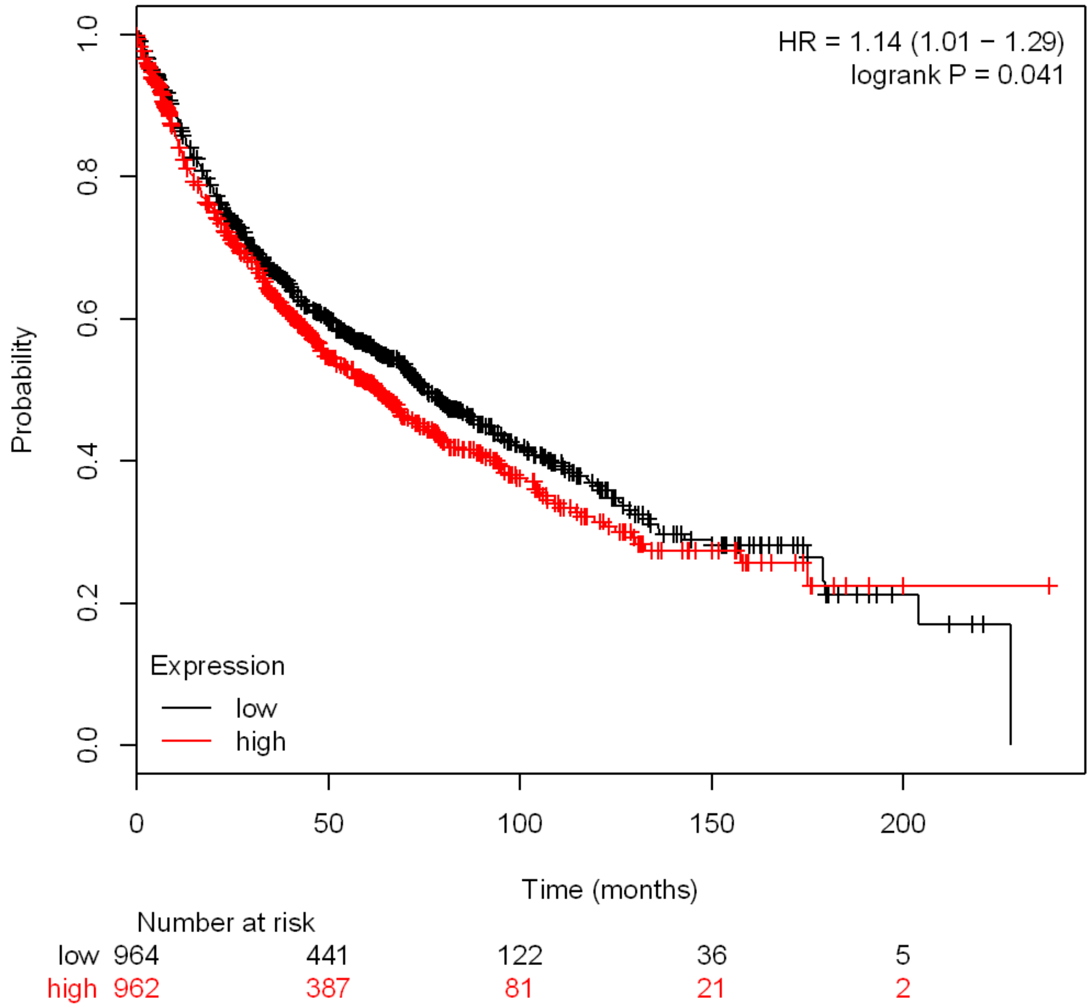

Figure S24 Kaplan–Meier plot for *MMP9* associated with patient survival.

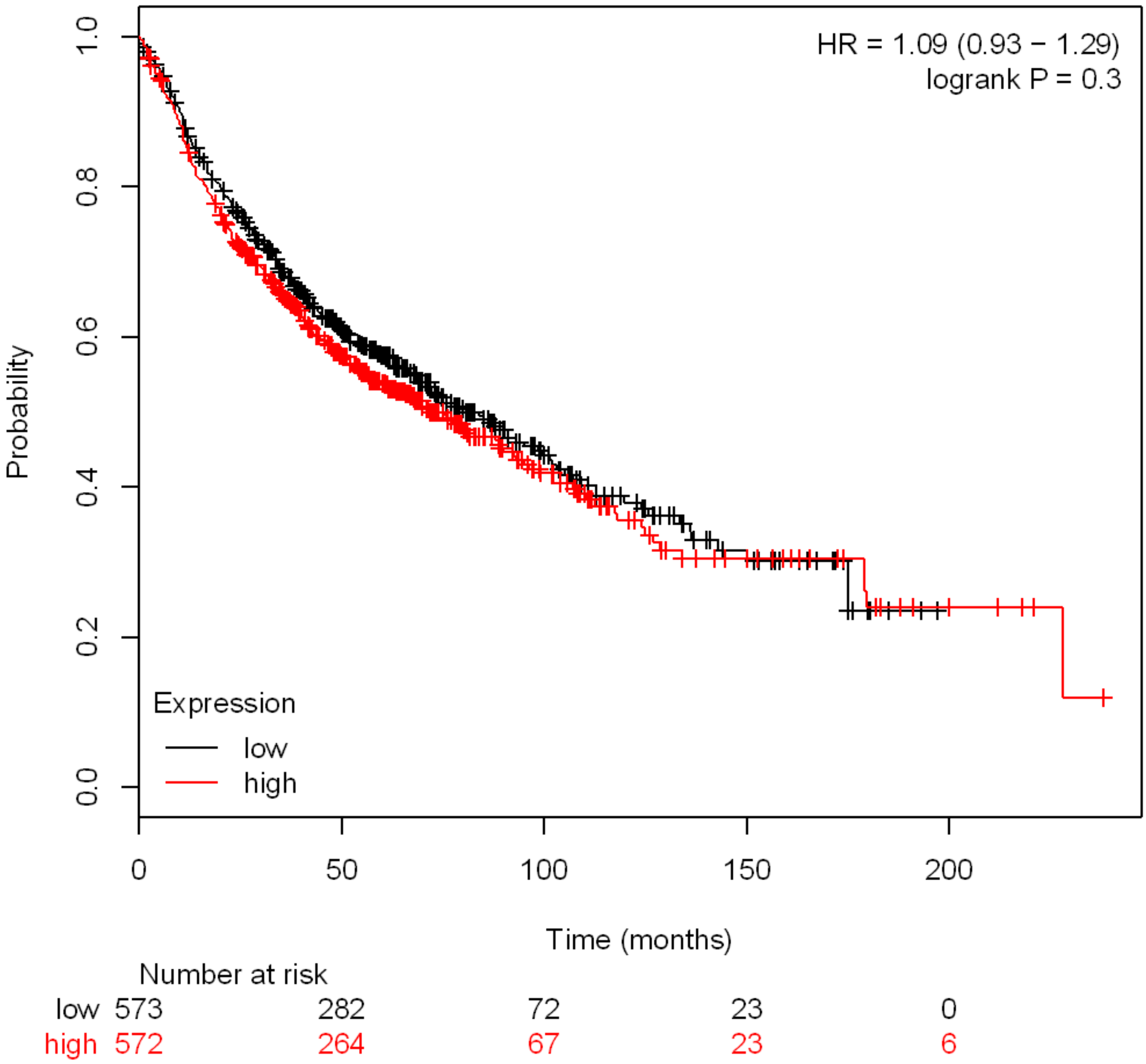

Figure S25 Kaplan–Meier plot for *NRAS* associated with patient survival.

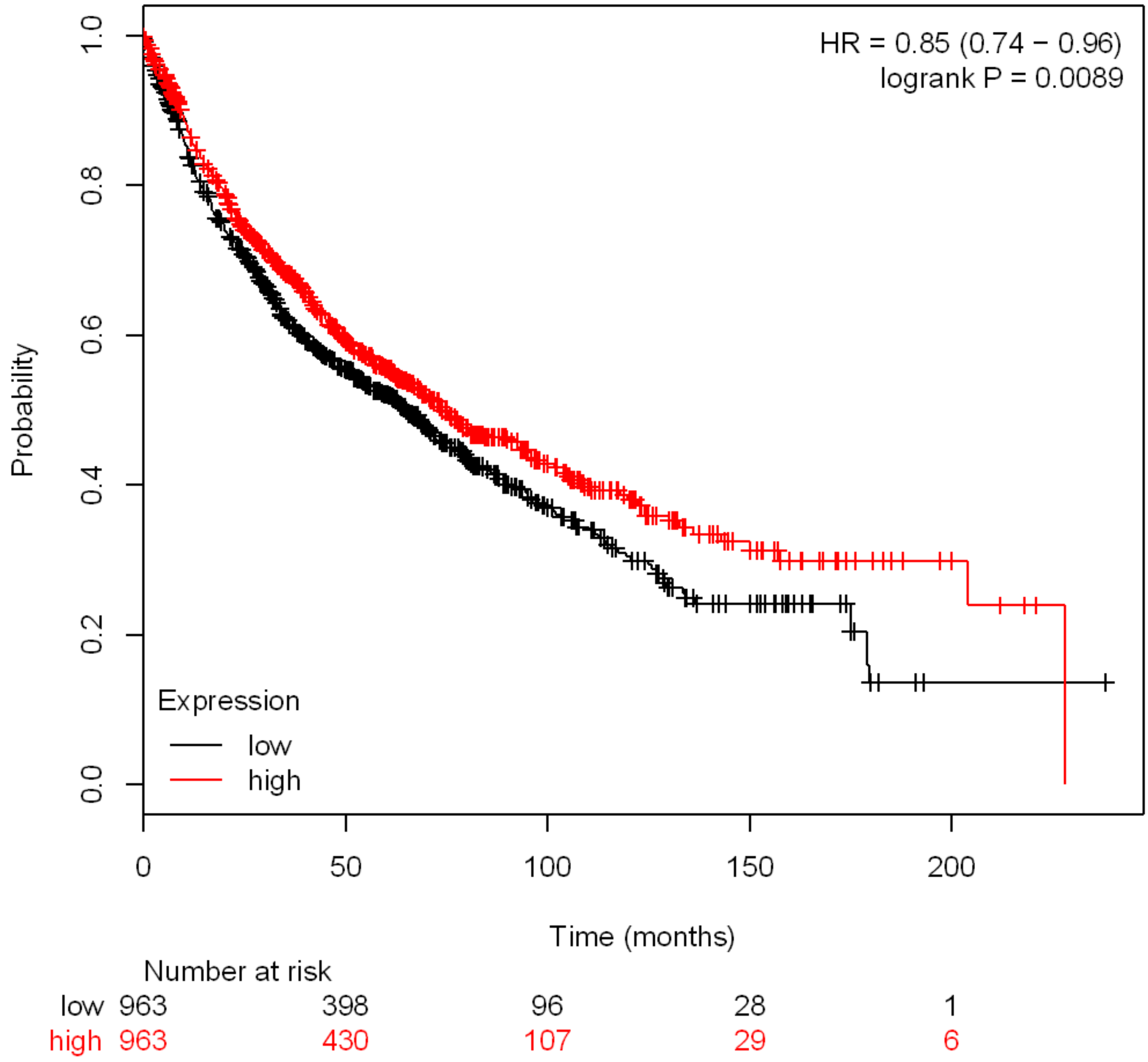

Figure S26 Kaplan–Meier plot for *MAPK13* associated with patient survival.

A

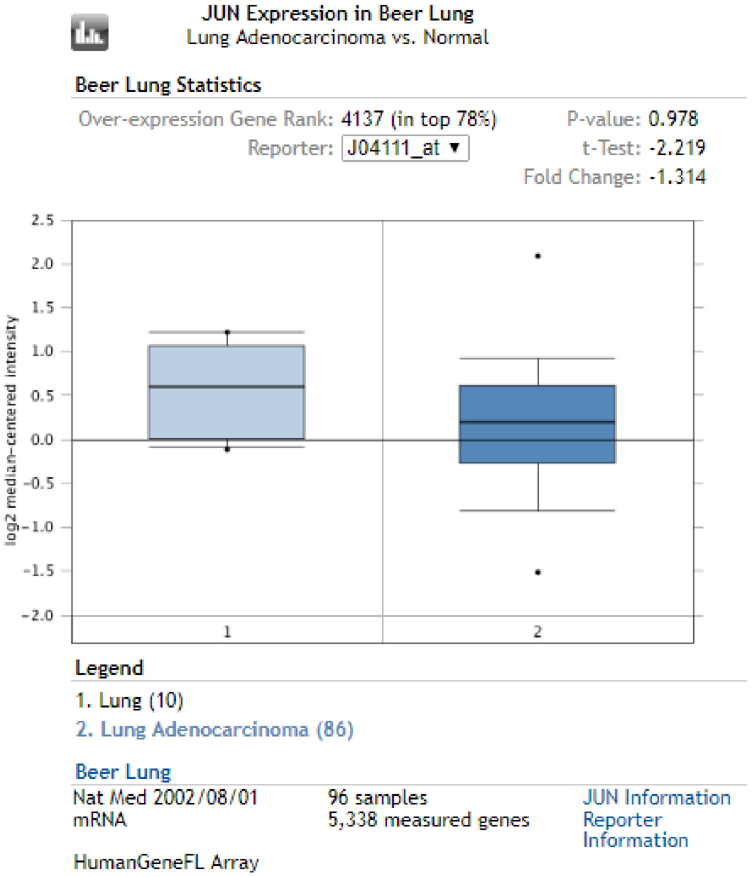

Figure S27 A Comparison of the expression of *JUN* between lung and lung adenocarcinoma samples in the Beer database using Oncomine.

A

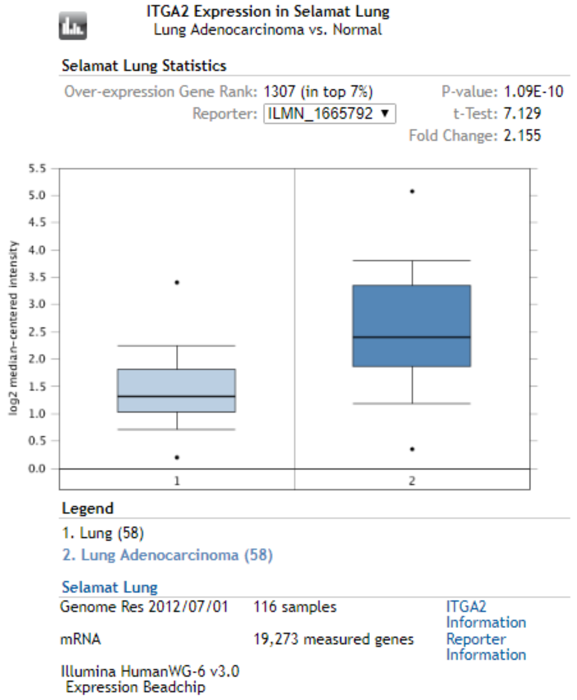

B

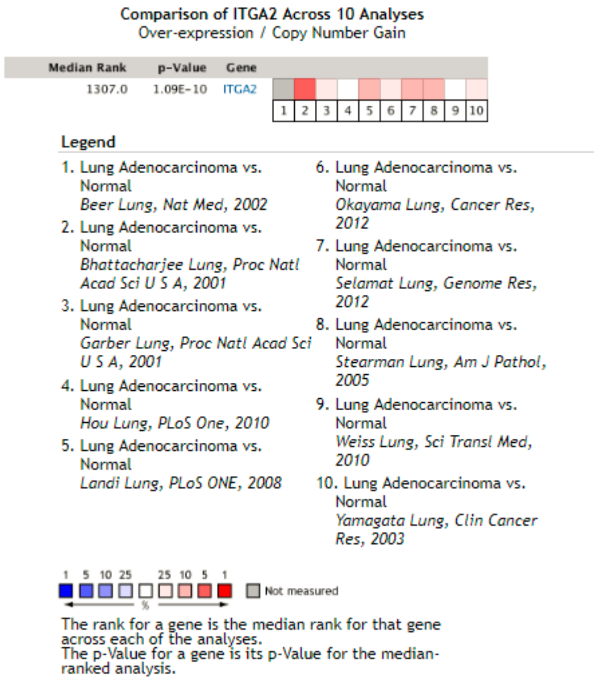

Figure S28 The *ITGA2* expressed in lung cancer by Oncomine. **A** Comparison of the expression of *ITGA2* between lung and lung adenocarcinoma samples in the Selamat database using Oncomine. **B** Ten analyses were evaluated in comparing the RNA expression of *ITGA2* between lung adenocarcinoma and normal tissue. Values above the average were considered *ITGA2* over-expression (red)

A

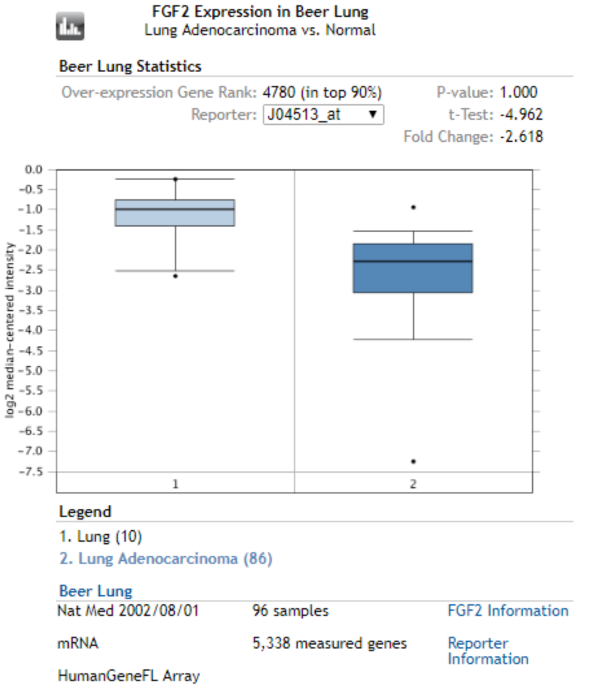

B

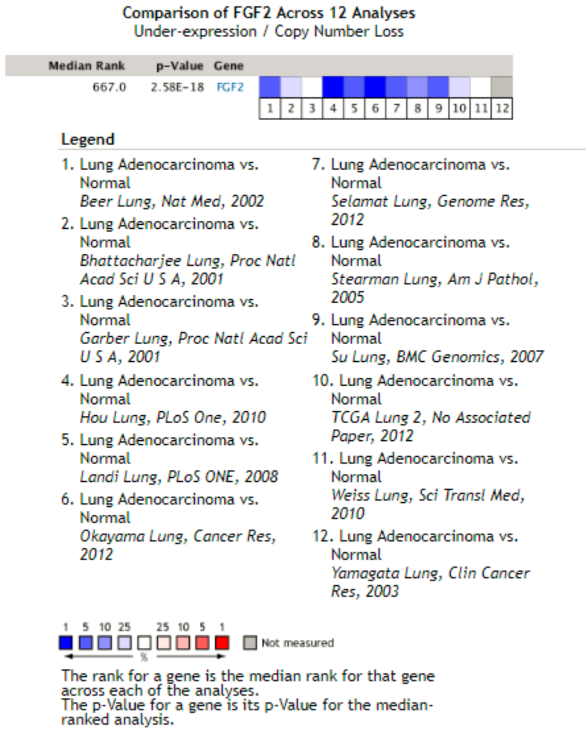

Figure S29 The *FGF2* expressed in lung cancer by Oncomine. **A** Comparison of the expression of *FGF2* between lung and lung adenocarcinoma samples in the Selamat database using Oncomine. **B** Twelve analyses were evaluated in comparing the RNA expression of *FGF2* between lung adenocarcinoma and normal tissue. Values below the average were considered *FGF2* lower-expression (blue).

A

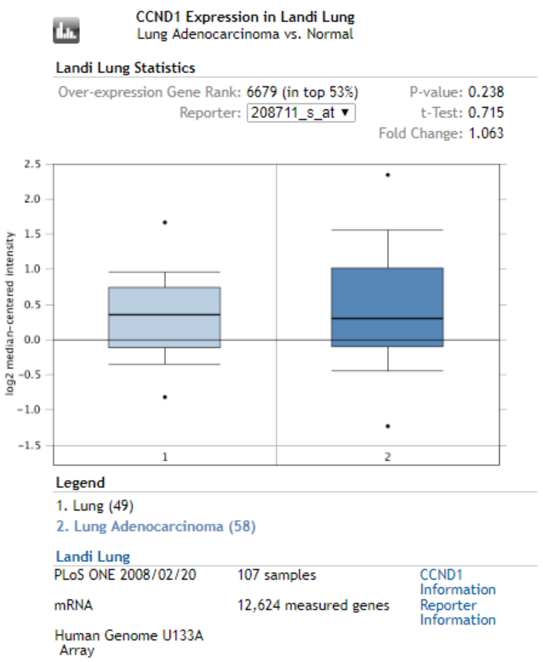

B

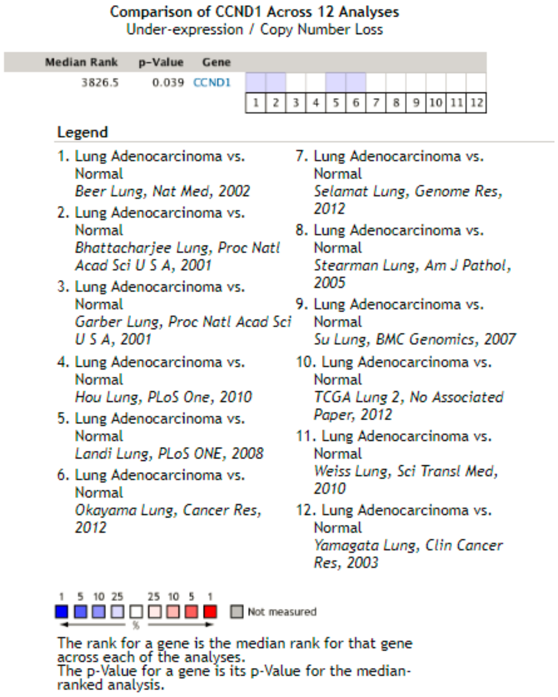

Figure S30 The *CCND1* expressed in lung cancer by Oncomine. **a** Comparison of the expression of *CCND1* between lung and lung adenocarcinoma samples in the Selamat database using Oncomine. **b** Twelve analyses were evaluated in comparing the RNA expression of *CCND1* between lung adenocarcinoma and normal tissue. Values below the average were considered *CCND1* lower-expression (blue).

A

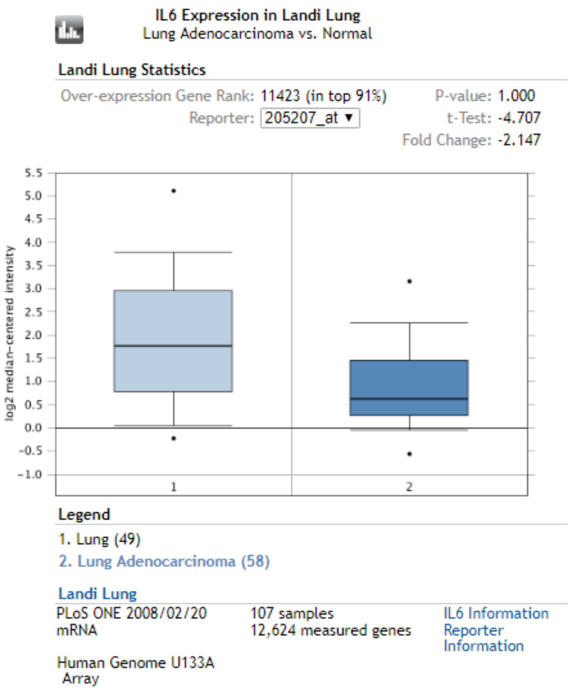

B

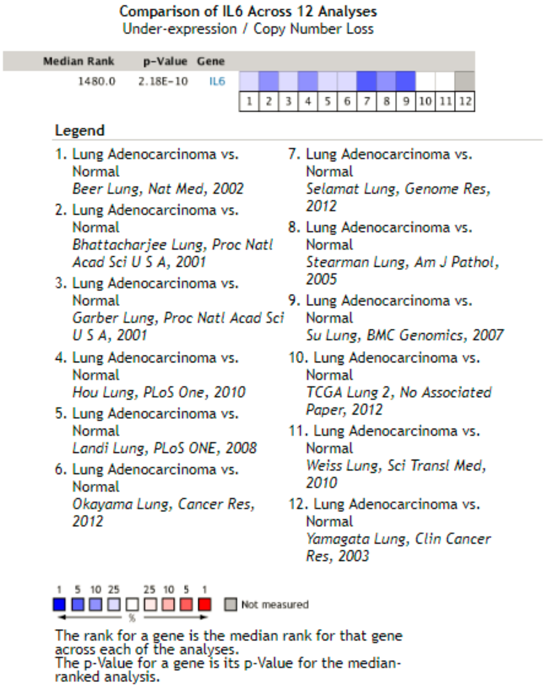

Figure S31 The *IL6* expressed in lung cancer by Oncomine. **a** Comparison of the expression of *IL6* between lung and lung adenocarcinoma samples in the Selamat database using Oncomine. **b** Twelve analyses were evaluated in comparing the RNA expression of *IL6* between lung adenocarcinoma and normal tissue. Values below the average were considered IL6 lower-expression (blue).

A

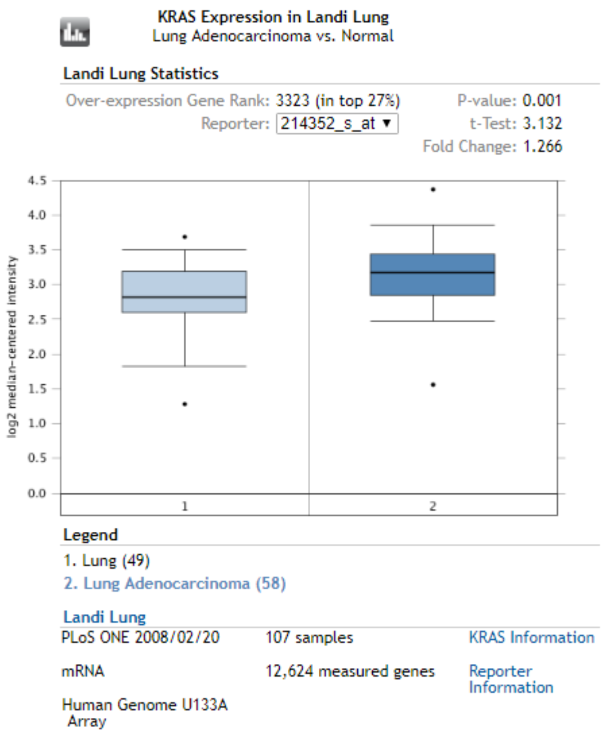

b

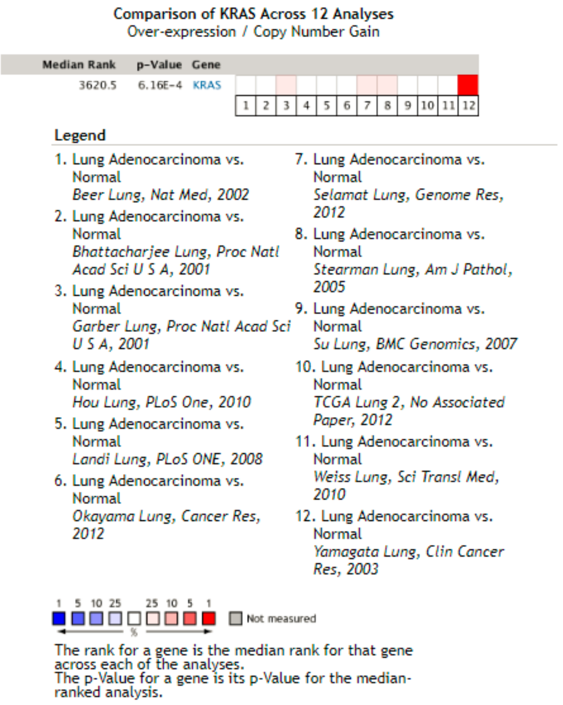

Figure S32 The *KRAS* expressed in lung cancer by Oncomine. **A** Comparison of the expression of *KRAS* between lung and lung adenocarcinoma samples in the Selamat database using Oncomine. **B** Twelve analyses were evaluated in comparing the RNA expression of *KRAS* between lung adenocarcinoma and normal tissue. Values above the average were considered *KRAS* over-expression (red).

A

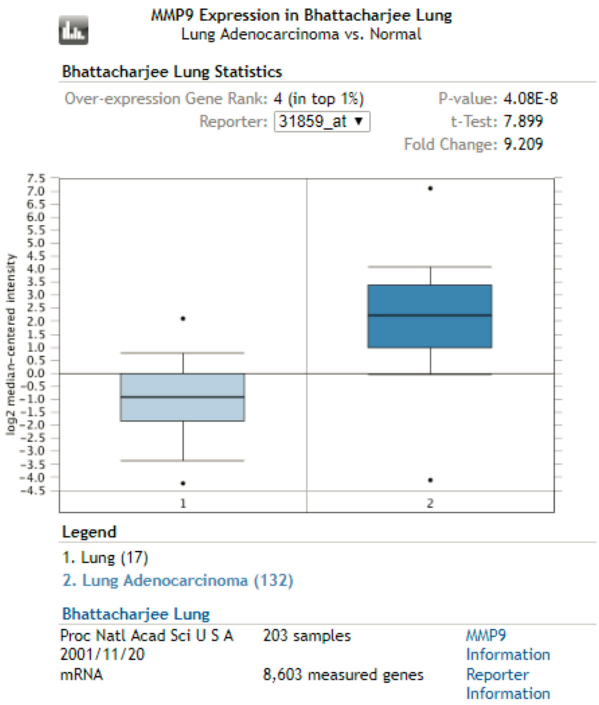

B

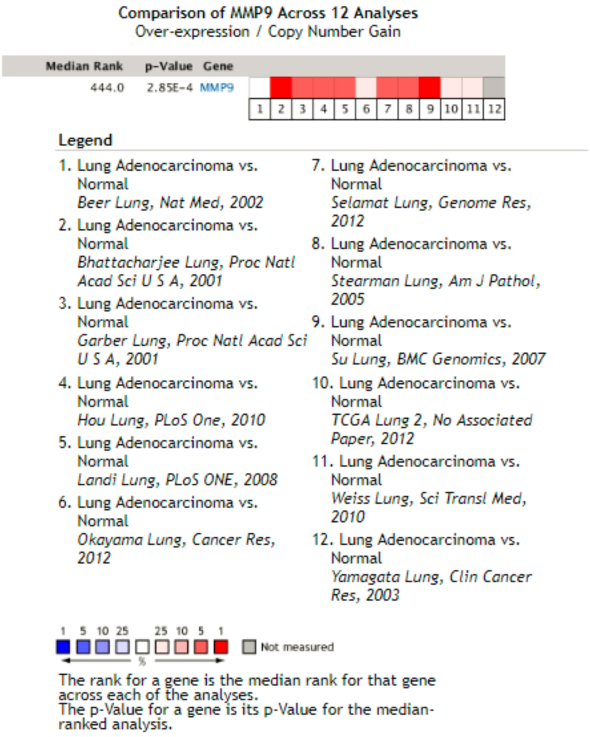

Figure S33 The *MMP9* expressed in lung cancer by Oncomine. **A** Comparison of the expression of *MMP9* between lung and lung adenocarcinoma samples in the Selamat database using Oncomine. **B** Twelve analyses were evaluated in comparing the RNA expression of *MMP9* between lung adenocarcinoma and normal tissue. Values above the average were considered *MMP9* over-expression (red).

A

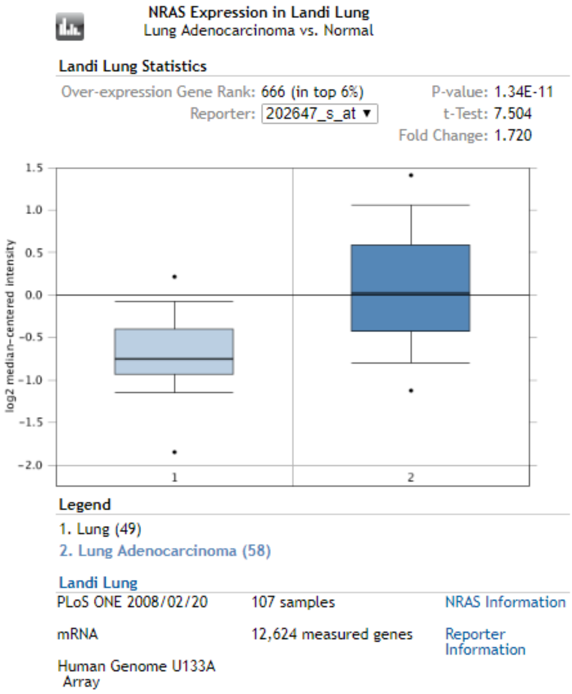

B

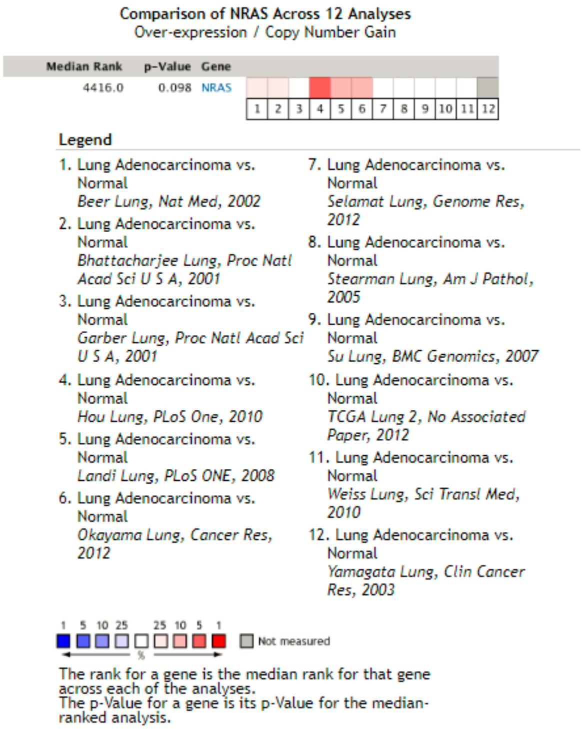

Figure S34 The *NRAS* expressed in lung cancer by Oncomine. **A** Comparison of the expression of *NRAS* between lung and lung adenocarcinoma samples in the Selamat database using Oncomine. **B** Twelve analyses were evaluated in comparing the RNA expression of *NRAS* between lung adenocarcinoma and normal tissue. Values above the average were considered *NRAS* over-expression (red).

A

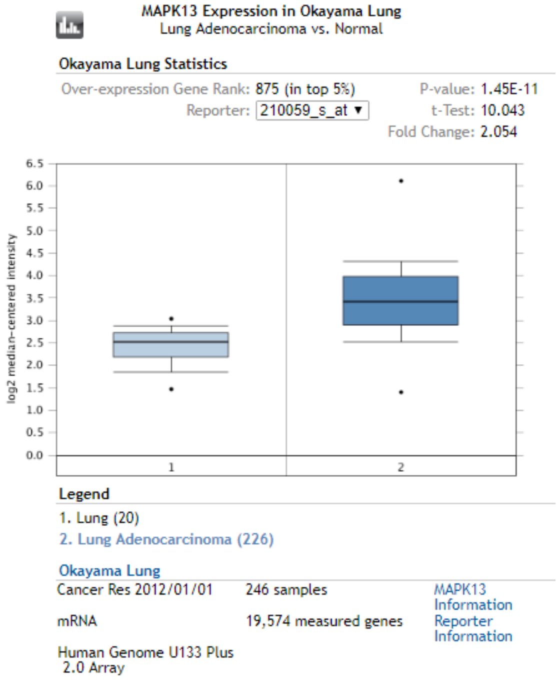

B

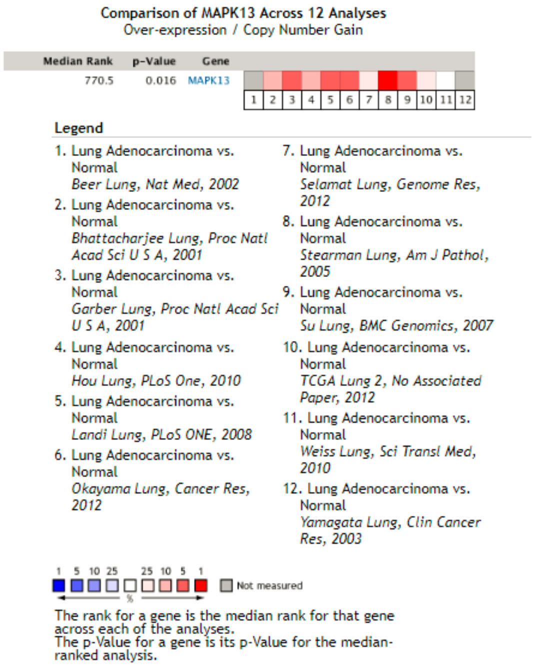

Figure S35 The *MAPK13* expressed in lung cancer by Oncomine. **A** Comparison of the expression of *MAPK13* between lung and lung adenocarcinoma samples in the Selamat database using Oncomine. **B** Twelve analyses were evaluated in comparing the RNA expression of *MAPK13* between lung adenocarcinoma and normal tissue. Values above the average were considered *MAPK13* over-expression (red).

Table S1 Hub proteins modelling

Hub proteins	Species	Protein length (aa)	Model templates (query cover, identify)
EGFR	Homo sapiens	1157	1ivo_A (40%, 99%)<br>3qwq_A (39%, 100%)<br>5xwd_A (37%, 99%)
JUN	Homo sapiens	331	*de novo*
ITGA2	Homo sapiens	1181	4NEH_A (75%, 30%)<br>3K6S_A (75%, 30%)<br>5ES4_A (75%, 30%)
FGF2	Homo sapiens	288	1II4_A (53%, 99%)<br>1BAS_A (53%, 99%)<br>1BLA_A (53%, 97%)
CCND1	Homo sapiens	295	2W96_A (91%, 100%)<br>2W9Z_A (86%, 100%)<br>3G33_B (98%, 52%)
IL6	Homo sapiens	212	1IL6_A (87%, 100%)<br>1ALU_A (87%, 100%)<br>1P9M_B (86%, 100%)
KRAS	Homo sapiens	189	5UFE_A (87%, 98%)

			5XCO_A (89%, 96%)
			4TQA_A (88%, 96%)
			1L6J_A (60%, 99%)
MMP9	Homo sapiens	707	1CK7_A (96%, 46%)
			1EAK_A (59%, 58%)
			3CON_A (91%, 100%)
NRAS	Homo sapiens	189	5UHV_A (87%, 100%)
			5XCO_A (89%, 91%)
			4EYJ_A (96%, 100%)
MAPK13	Homo sapiens	365	3COI_A (96%, 99%)
			4MYG_A (96%, 99%)

Table S2 KEGG pathway analysis of up-regulated and down-regulated DEGs

Expression	Pathway ID	Name	Gene count	%	Genes
Up-regulated	hsa05200	Pathways in cancer	10	8.62	*FGF1, ITGA2, LAMB3, LAMC2, MMP1, LEF1, PDGFB, CCND1, RET, TGFBR2*
	hsa01100	Metabolic pathways	9	7.76	*ME3, CYP11A1, HSD11B1, ITPKB, NT5E, OLAH, ALDH8A1, B3GNT5, B3GALNT1*
	hsa04621	NOD-like receptor signaling pathway	7	6.03	*TXNIP, GBP1, GBP3, IFI16, MAPK13, CCL5, ANTXR1*
	hsa04151	PI3K-Akt signaling pathway	6	5.17	*FGF1, ANGPT2, ITGA2, LAMB3, LAMC2, PDGFB, CCND1*
	hsa04510	Focal adhesion	6	5.17	*ITGA2, LAMB3, LAMC2, PDGFB, PAK6, CCND1*
Down-regulated	hsa05164	Influenza A	8	10.00	*ICAM1, IL1A, CXCL8, MX1, OAS2, PRSS1, PRSS2, CCL5*
	hsa05200	Pathways in cancer	7	8.75	*FGFR2, CXCL8, JUN, PDGFRA, RAC2, CXCL12, TCF7L2*
	hsa04010	MAPK signaling pathway	6	7.50	*FGFR2, CXCL8, JUN, PDGFRA, RAC2, CXCL12, TCF7L2*
	hsa05323	Rheumatoid arthritis	6	7.50	*ICAM1, IL1A, CXCL8, JUN, CCL5, CXCL12*
	hsa04060	Cytokine-cytokine receptor interaction	6	7.50	*IL1A, CXCL8, INHBA, NGF, CCL5, CXCL12*

Table S3 The distribution of the hub genes in cells.

Gene name	Cell lines	Main location
EGFR	A-431, U-251	plasma membrane
JUN	A-431, U-2 OS, U-251	nucleoplasm
ITGA2	HaCaT, U-2 OS	nucleus, cytosol
FGF2	A-431, U-2 OS, U-251MG	nucleoplasm, nuclear bodies
CCND1	BJ, U-2 OS	nucleoplasm
IL6	MCF7, U-2 OS	vesicles
MMP9	A-431, U-2 OS, U-251MG	mitochondria
MAPK13	A-431, RT4, U-2 OS	nucleus, nucleoli
KRAS	NA	NA
NRAS	NA	NA

NA: none available

Table S4 Radius of Gyration (R*g*) analysis

**Hub proteins**	**R*g* value (Minimum)**	**R*g* value (Max)**	**R*g* value (Average)**
EGFR	3.37	3.49	3.42
JUN	2.15	2.45	2.24
ITGA2	4.74	6.25	5.53
FGF2	1.44	1.60	1.47
CCND1	2.01	2.19	2.06
IL6	1.60	1.69	1.64
KRAS	1.60	1.70	1.63
MMP9	2.77	3.03	2.84
NRAS	1.64	1.74	1.69
MAPK13	2.22	2.36	2.27

Table S5 Ramachandran plot analysis

Hub proteins	Number of residues in favoured region	Number of residues in allowed region	Number of residues in outlier region
EGFR	869 (76.2%)	220 (19.3%)	52 (4.6%)
JUN	302 (92.1%)	22 (6.7%)	4 (1.2%)
ITGA2	957 (81.2%)	180 (15.3%)	41 (3.3%)
FGF2	132 (85.7%)	19 (12.3%)	3 (1.9%)
CCND1	272 (93.2%)	20 (6.8%)	0 (0.0%)
IL6	148 (90.8%)	13 (8.0%)	2 (1.2%)
KRAS	171 (91.9%)	13 (7.0%)	2 (1.1%)
MMP9	610 (86.6%)	74 (10.5%)	20 (2.8%)
NRAS	167 (88.2%)	14 (7.5%)	5 (2.7%)
MAPK13	324 (89.8%)	29 (8.0%)	9 (2.5%)

Supplementary References

1 Thul, P. J. *et al.* A subcellular map of the human proteome. *Science* **356** (2017).
